# Modular synthetic routes to biologically active indoles from lignin†

**DOI:** 10.1039/d5gc01003a

**Published:** 2025-06-10

**Authors:** Antonio A. Castillo-Garcia, Jörg Haupenthal, Anna K. H. Hirsch, Katalin Barta

**Affiliations:** a Institute of Chemistry, University of Graz Heinrichstrasse 28 A-8010 Graz Austria katalin.barta@uni-graz.at; b Stratingh Institute for Chemistry, University of Groningen Nijenborgh 4 9747 AG Groningen The Netherlands; c Helmholtz Institute for Pharmaceutical Research Saarland (HIPS) − Helmholtz Centre for Infection Research (HZI) Campus Building E8.1 66123 Saarbrücken Germany; d PharmScienceHub Campus Building A2.3 66123 Saarbrücken Germany; e Saarland University, Department of Pharmacy Campus Building E8.1 66123 Saarbrücken Germany

## Abstract

Diol-assisted fractionation has emerged as an important ‘lignin-first’ processing method that delivers aromatic C2-acetals with high selectivity. This contribution describes the development of an unexpectedly straightforward synthetic route to biologically active indoles from this aromatic platform chemical, boosting the scope of this unique biorefinery approach. The novel method utilizes the functionalization of C2-acetal *via* phenol alkylation and mild halogenation reactions, enabling catalytic C–N coupling with anilines and benzylamines and forging *ortho*-aminoacetal intermediates. Such derivatives are suitable for *in situ* Schiff base formation/intramolecular cyclization by acetal deprotection in a mixture of MeOH/H_2_O and PTSA as the catalyst, resulting in a novel library of lignin-based indoles. Evaluation of the biological activity in terms of anticancer activity using human Hep G2 cells shows promising early results.

## Abstract 2

Modular functionalization of a lignin-derived C2-acetal enables the synthesis of bio-based indoles *via* the formation of *ortho*-amino acetal derivatives and the *in situ* Schiff-base formation/intramolecular cyclization under mild reaction conditions.

## Graphical abstract

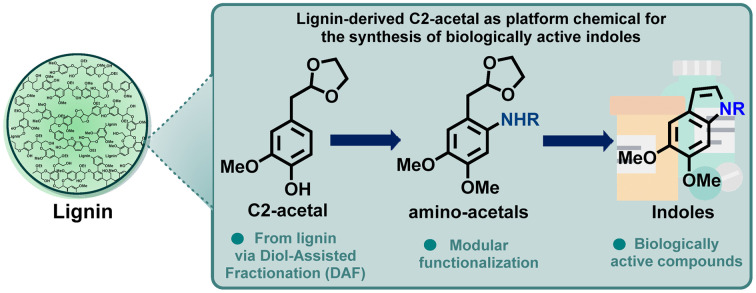

**Green foundation**

The ubiquity of indoles as building blocks in a plethora of pharmaceuticals demands much greener and more sustainable synthetic strategies incorporating two fundamental aspects:

1. The valorization of renewable aromatic platform chemicals such as lignin with the full incorporation of intrinsic functionalities in the final products ensures high-atom economical processes and, at the same time, alleviates the high dependence on fossil-based resources.

2. The use of catalytic strategies and mild reaction conditions in combination with more environmentally friendly solvents and reagents is highly desired, perfectly in line with the principles of Green Chemistry, and addresses the UN SDGs-2030, **GOAL-3** (Good Health and Well-being) and **GOAL-12** (Responsible Consumption and Production).

## Introduction

**Fig. 1:**
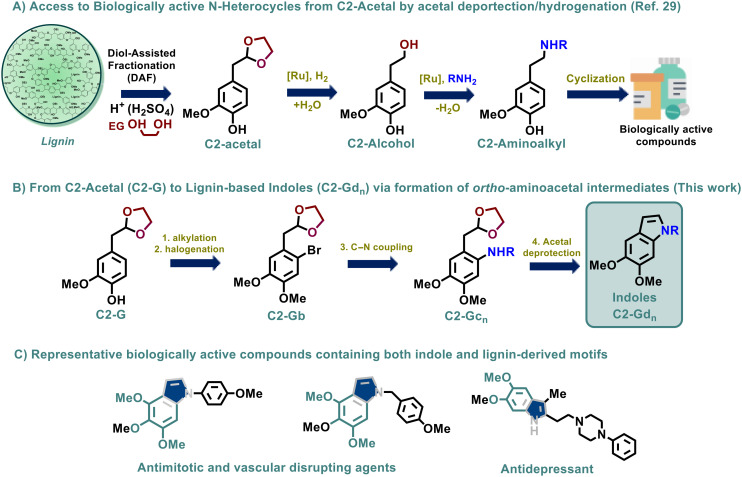
General strategy for the synthesis of bio-active indoles from lignin and relevant biologically active compounds bearing lignin-derived functionalities.

Naturally occurring in tryptophan and several alkaloids, indole motifs^1,2^ are privileged moieties given their omnipresence in multiple biologically active compounds^3–6^ and versatility towards further transformations.^7,8^ Throughout the years, traditional strategies such as the Fischer, Bischler and Baeyer–Emmerling indole syntheses, among others, have been explored in depth, enriching the diversity of these derivatives;^9–11^ however, the design of greener pathways in combination with the use of renewable building blocks would still be highly advantageous.^12,13^ In that sense, several approaches for the sustainable synthesis of indoles have been recently described in the literature, including the use of benign solvents, such as mixtures of water and ethanol, deep eutectic solvents (DESs), bio-based ethyl lactate or biodegradable polyethylene glycol, and the use of green techniques such as microwave/UV-visible light irradiation or ultrasound-assisted synthesis,^14^ however, the use of renewable starting materials in this arena remains scarcely explored.^15^ We have previously described the utilization of lignin-derived building blocks for the synthesis of N-heterocycles providing green access to a handful of pharmaceutically relevant scaffolds^16^ conventionally obtained from petrochemicals, paving the way for the diversification of high-value chemicals from biomass streams.^17,18^ To the best of our knowledge, however, only one example has been reported employing lignin model compounds for the synthesis of indoles. In that work, lignin models bearing α-hydroxyacetophenones were reacted with pyrroles *via* [4 + 2] annulation.^19^ Furthermore, the valorization of lignin-derived mono-aromatics as platform chemicals^20,21^ has emerged as a sustainable alternative where the intrinsic moieties from parent lignin can be conveniently modified towards the production of nitrogen-containing compounds.^22–24^ Following this principle, our group has recently reported the atom-efficient valorisation of the lignin-derived acetal C2-G, selectively obtained *via* acidolysis of softwood lignin in conjunction with ethylene glycol (EG) stabilization,^25–28^ into a novel series of biologically active N-chemicals such as dopamine derivatives, tetrahydroisoquinolines, and quinazolinones as well as the natural product tetrahydropapaveroline in clean synthetic pathways, principally relying on the amination of the aliphatic alcohol side chain (Fig. 1A).^29^

Herein, we aimed to develop a different methodology, aligning with the inherent structural features of C2-G, but manipulating the aromatic core first to target the formation of a new series of lignin-based N-heterocycles, whereby ring formation is promoted by the presence of the ‘masked’ aldehyde, inherently present in the C2-G platform chemical.

In this regard, the formation of indoles, employing acetal derivatives, has previously been attempted. For instance, the commonly named Nordlander synthesis^30^ involves the use of acetals in an intramolecular cyclization with anilines and sulfonamides.^31,32^ Moreover, we expected that the inherent acetal moiety might facilitate the direct aromatic halogenation of C2-G and the subsequent nitrogen incorporation by C–N coupling, avoiding undesired side reactions.

A similar approach has indeed been reported for the synthesis of petrol-based isoquinolines, employing aryl halides containing acetal moieties in the *ortho*-position as convenient starting building blocks.^33^ Based on these antecedents, our new methodology for the synthesis of a series of lignin-based indoles contemplates (a) the efficient functionalization of C2-G by phenol alkylation/SEAr reactions, followed by (b) the Pd-mediated C–N coupling of C2-Gb with anilines and benzylamines and (c) acid-catalyzed acetal deprotection leading to *in situ* Schiff base formation and intramolecular cyclization towards the formation of indoles (C-2Gd_*n*_) (Fig. 1B). Interestingly, numerous indoles containing intrinsic lignin-like functionalities have shown promising biological activity, for instance as anticancer^34,35^ or antidepressive^36^ agents (Fig. 1C); therefore, the pharmaceutical relevance of the new lignin-derived indoles C2-Gd_*n*_ was evaluated in terms of their anticancer activity on human Hep G2 cells, displaying promising activity for several of these derivatives.

## Results and discussion

### Establishing a catalytic strategy for the construction of lignin-based indoles (C2-Gd_*n*_)

**Scheme 1:**
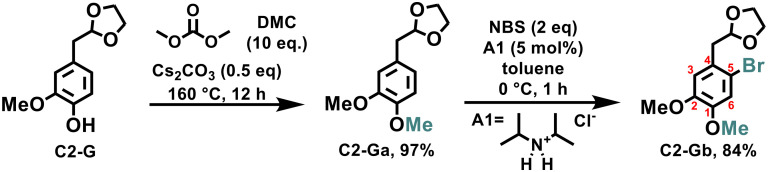
Synthesis of intermediate C2-Gb (for experimental details, see the ESI†).

We began our study with the synthesis of C2-Gb through a convenient two-step method. First, the reaction of C2-G with dimethyl carbonate (DMC) as a non-toxic alkylating agent^37^ led to the formation of C2-Ga in an almost quantitative yield (97%). Then, the formation of C2-Ga allowed selective bromination in the *ortho* position (C5) to the aliphatic chain, employing NBS and di-isopropyl ammonium chloride (A1) as additives under mild conditions, affording an isolated yield of 84% (Scheme 1). Next, the catalytic C–N coupling of C2-Gb with aniline was investigated.

For this purpose, well-established Pd-protocols^38^ were tested, observing that the combination of Pd_2_dba_3_ (1 mol%) with Xantphos^39^ (2 mol%) as the ligand and Cs_2_CO_3_ as the base provided the best results, affording full conversion and good isolated yield (80%) of the target product (Table 1, entry 1). Similar results were obtained when Pd(dba)_2_^40^ or Pd(MeCN)Cl_2_ was employed as the catalyst; however, a significant loss of activity was observed when different phosphines were utilized instead of Xantphos (Table 1, entries 4–6). Additionally, Ni-catalysts Ni(cod)_2_ and Ni(dme)Cl_2_ were also tested under the same reaction conditions without achieving conversion; therefore, we decided to continue our study using the aforementioned Pd_2_dba_3_/Xantphos system. The influence of temperature and reaction time was evaluated when the reaction was performed either at 100 °C or for 8 h (Table 1, entries 11 and 13), observing lower conversion in both cases. Finally, we found that the use of a small excess of aniline was beneficial since a loss of yield was detected when the reaction was performed using a stoichiometric ratio (1 eq.) of aniline (entry 9).

**Table 1:** Pd-catalyzed C–N coupling of C2-Gb with anilines and benzylamines^a^

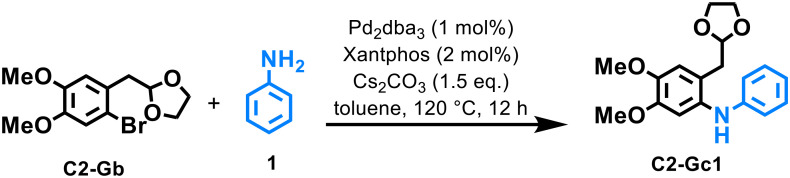		
Entry	Deviation from standard conditions	C2-Gc1 b (%)
1	—	**91 (80)** c
2	Pd(dba)_2_ as precatalyst	86
3	Pd(MeCN)Cl_2_ as precatalyst	89
4	Dppp as ligand	0
5	Dppf as ligand	23
6	DPEPhos as ligand	35
7	Ni(cod)_2_ as precatalyst	0
8	Ni(dme)Cl_2_ as precatalyst	0
9	1 eq. of 1	76
10	*T* = 100 °C	56
11	NaOtBu instead of Cs_2_CO_3_	86
12	*t* = 8 h	64

a General reaction conditions: C2-Gb (0.28 mmol), 1 (0.30 mmol), Pd_2_dba_3_ (2.8 × 10^−3^ mmol), Xantphos (5.6 × 10^−3^ mmol), Cs_2_CO_3_ (0.42 mmol), 120 °C, 12 h.

b Yields were determined by GC-FID.

c Isolated yield.

With the optimized conditions in hand, we turned our attention to evaluate the scope of the C–N coupling between C2-Gb with diverse aromatic amines. A host of differently substituted anilines were tested obtaining moderate to good yields in most of the cases, including those anilines bearing electron-donating groups such as –OMe (C2-Gc3) and –SMe (C2-Gc7), as well as those featuring electron-withdrawing groups like –CN (C2-Gc2), –CF_3_ (C2-Gc5 and C2-Gc6), –F (C2-Gc9 and C2-Gc10) and –NO_2_ (C2-Gc11). Moreover, anilines bearing carbonyl groups such as –COMe (C2-Gc4) and –COOMe (C2-Gc8) were also subjected to C–N coupling with C2-Gb affording good yields in both cases (Table 2). In addition, the coupling with potentially lignin-based benzylamines^41^ was efficiently performed achieving acceptable yields in the cases of C2-Gc13 and C2-Gc14. Interestingly, the influence of the steric hindrance from different functional groups was noted especially in the case of those derivatives containing benzylamines or anilines containing substituents such as –COMe and –SMe. Although the deprotection of acetals mediated by Pd-catalysis has been previously reported,^42,43^ the reaction nonetheless has to be performed in aqueous media and preferably under acidic conditions; therefore, the C–N coupling of C2-Gb was smoothly achieved under the above-mentioned anhydrous conditions without acetal deprotection being observed; therefore, the C–N coupling of C2-Gb was smoothly achieved under the abovementioned anhydrous conditions.

**Table 2:** Pd-catalyzed C–N coupling of C2-Gb with anilines and benzylamines^a^

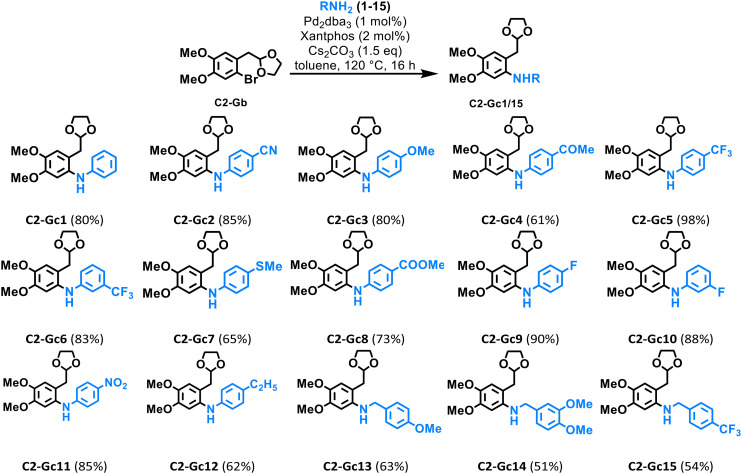

a General reaction conditions: C2-Gb (0.28 mmol), 1–15 (0.3 mmol), Pd_2_dba_3_ (2.8 × 10^−3^ mmol), Xantphos (5.6 × 10^−3^ mmol), Cs_2_CO_3_ (0.42 mmol), 120 °C, 16 h. Isolated yields are shown.

In order to establish the reaction conditions for the acetal deprotection of C2-Gc1 and *in situ* Schiff-base formation, a mixture of MeOH/H_2_O was employed as the reaction medium given the low solubility of these derivatives in water, which was imperative to perform this reaction (Table S2†). Previously, the deprotection of 1,3-dioxolanes containing sensitive functional groups has been carried out using aqueous methanol under reflux and InCl_3_ as the Lewis acid catalyst.^44^ Indeed, the use of an acidic additive is crucial for both acetal deprotection and indole formation, where Brønsted acids such as HCl or *p*-toluene sulfonic acid (PTSA) are commonly applied;^45^ therefore, we decided to use PTSA as the additive for our study. As expected, the formation of the indole C2-Gd1 was achieved almost quantitatively when the reaction was performed in MeOH/H_2_O (1 : 1) at 140 °C for 1 h using 0.1 eq. of PTSA, affording an isolated yield of 80% (Table S2,† entry 1). A significant loss of conversion was observed when only MeOH or H_2_O was used as the solvent (Table S2,† entries 2 and 3). Moreover, the acetal deprotection was found to be temperature dependent, since only 69% conversion was observed when performing the reaction at 120 °C (Table S2,† entry 5). The effect of the additive was also tested by using oxalic acid instead of PTSA (entry 6), albeit leading to diminished conversion. After establishing the reaction conditions for the synthesis of C2-Gd1, the scope of this method was evaluated for several *ortho*-aminoacetal derivatives C2-Gc_*n*_ (Table 3). Overall, compounds bearing aniline scaffolds delivered good to excellent yields in comparison with substrates containing benzylamines, which can be attributed to the higher basicity of benzylamines and to the plausible Nordlander-indole synthesis mechanism for intramolecular cyclization that requires strong acidic conditions.^9^ In fact, the formation of indoles starting from α-anilinoacetal intermediates typically requires the use of harsh chemicals such as trifluoroacetic acid and halogenated solvents; therefore, our method provides a more sustainable pathway for the synthesis of these moieties by using a greener reaction medium such as MeOH/H_2_O in combination with catalytic amounts of acid.

**Table 3:** Lignin-based indoles C2-Gd_*n*_*via ortho*-aminoacetal deprotection: scope of the reaction^a^

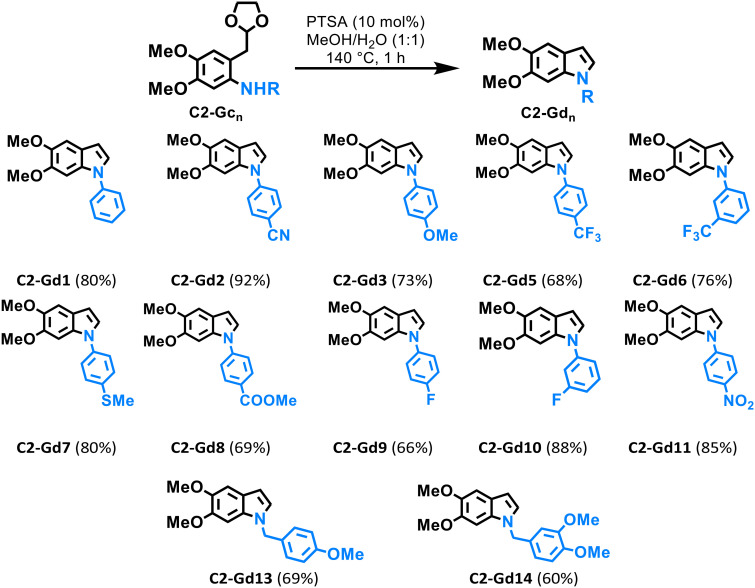

a General reaction conditions: C2-Gc_*n*_ (0.15 mmol), PTSA (0.015 mmol), MeOH/H_2_O (1 : 1, 2 mL) 140 °C, 1 h. Isolated yields are shown.

### Evaluation of biological activity of indoles C2-Gd_*n*_

**Fig. 2:**
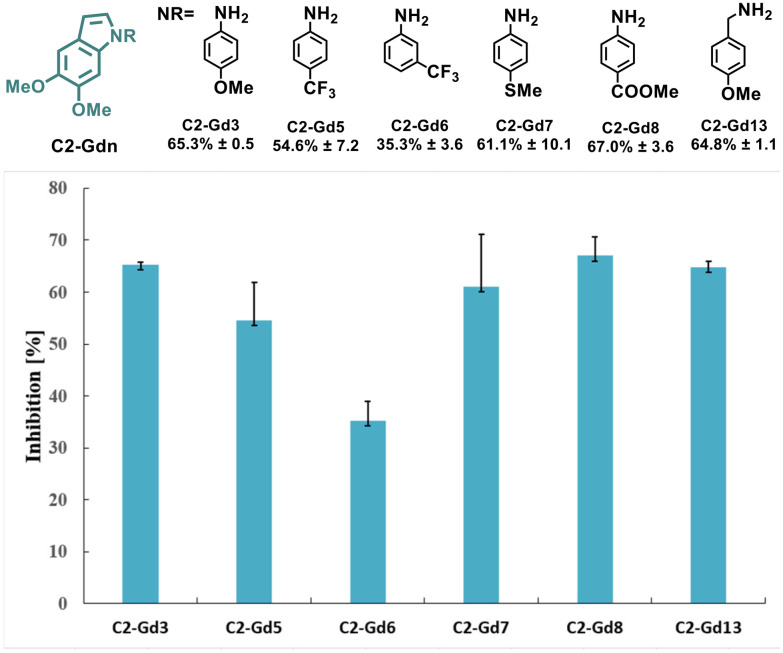
Inhibitory effects of selected examples of lignin-based indoles C2-Gd_*n*_ on the viability of HepG2 cells treated with the indicated compounds at 100 μM. Percentage (%) inhibition values and standard deviations from at least two independent experiments are indicated.

The intrinsic functionalities of the lignin-derived C2-acetal C2-G not only allow sustainable access to indoles but might also increase the biological activity given their occurrence in multiple natural products. In fact, the impact of methoxy substituents on the structural rigidity of indoles and their antiproliferative activity in various human cancer cell lines has been previously identified.^46^ In order to determine the bioactivity of the new series of lignin-based indoles, the effects on a human hepatoma cell line (Hep G2) as an early indication of anticancer activity of selected C2-Gd_*n*_ derivatives was explored (Fig. 2).

Overall, a common structure−activity trend was observed. Indoles with electron-donating substituents such as –OMe (C2-Gd3 and C2-Gd13), –SMe (C2-Gd7) or electron-withdrawing –COOMe (C2-Gd8) displayed higher inhibition in comparison with those with strongly deactivating substituents such as –CF_3_ (C2-Gd5 and C2-Gd6) or –F (C2-Gd9), and led to a lower activity. These results reveal the potential application of these novel scaffolds as anticancer agents. However, further optimization and more rigorous drug design are nonetheless required in order to enhance the biological activity of these molecules.

### Qualitative assessment of green credentials for the synthesis of C2-Gd_*n*_

Evaluation of the synthetic methodology was performed in terms of the CHEM21^47^ green metrics toolkit at the first pass level, and the results are summarized in Table 4. The first enhancement of the greenness of this method is observed during the phenol alkylation with dimethyl carbonate, allowing the formation of C2-G under neat conditions with a practical work-up procedure and displaying high atom-economy (AE) and remarkable overall economy (OE). Nonetheless, the halogenation step underperforms in terms of process mass intensity due to the usage of stoichiometric amounts of brominating agent (NBS) and solvent, as well as in health/safety aspects and work-up procedures where flash chromatography is required to isolate the desired product. The amination step towards the formation of *ortho*-aminoacetals C2-Gc_*n*_ proceeds under catalytic conditions employing 1 mol% of Pd_2_(dba)_3_, which displays a significantly higher activity compared to earth-abundant Ni catalysts, although the stoichiometric use of Cs_2_CO_3_ is nonetheless required, increasing the process mass intensity (PMI) of this step.^48^ Finally, a successful acetal deprotection/cyclization step can be afforded by using environmentally benign solvent mixtures such as MeOH/H_2_O with the use of catalytic amounts of PTSA, affording satisfactory values in terms of atom economy (AE) and overall economy (OE).

**Table 4:** Qualitative assessment of solvent use, inherent hazards of used chemicals, catalyst or reagent use, energy and workup methods for the synthesis of indoles C2-Gd_*n*_ a

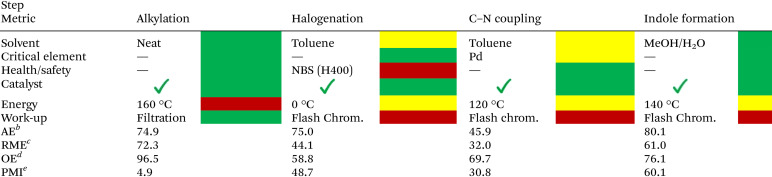

a Values calculated for the synthesis of 5,6-dimethoxy-1-(4-methoxyphenyl)-1*H*-indole (C2-Gd3) from C2-G.

b AE (atom economy) = (molecular weight of product/total molecular weight of reactants) × 100.

c RME (reaction mass efficiency) = (mass of isolated products/total mass of reactants) × 100.

d OE (overall economy) = RME/AE × 100.

e PMI (process mass intensity) = total mass process step (reagents + reactants + catalyst + solvent)/total mass product.

## Conclusions

The efficient incorporation of intrinsic functionalities of the lignin-derived mono-aromatic C2-acetal (C2-G) for the construction of bio-derived indoles has been successfully accomplished following a modular procedure involving the use of mild reaction conditions, safe solvents and catalytic methods. Since we have previously identified C2-acetal as a central lignin-derived platform chemical, this work represents a unique and meaningful pathway towards N-chemicals *via* ‘lignin-first’ biorefining, with particular relevance for the pharmaceutical industry. Evaluation of the biological activity as anticancer agents has shown the potential of the novel lignin-derived indoles; nevertheless, further structural modification, likely *via* the indole moiety, could enhance the activity of these derivatives, leading to the design and optimization of promising pharmaceuticals from renewable resources.

## Author contributions

AC: conceptualization, investigation, original draft writing and editing; JH: investigation and writing; AKHH: supervision, review,editing and funding acquisition; KB: conceptualization, writing, review and editing, funding acquisition and supervision.

## Conflicts of interest

There authors declare no conflict of interest.

## Supplementary Material

GC-027-D5GC01003A-s001

## Data Availability

The data supporting this article have been included as part of the ESI.†
