# Supplementary material for: Modular synthetic routes to biologically active indoles from lignin
Source: Green Chem. 2025 Jun 10;27(25):7506–12. doi: 10.1039/d5gc01003a (PMC12151140; doi:10.1039/d5gc01003a)
Supplement: GC-027-D5GC01003A-s001 [file GC-027-D5GC01003A-s001.pdf]

Supplementary Information (SI) for Green Chemistry.
This journal is © The Royal Society of Chemistry 2025

Supplementary information
Modular synthetic routes to biologically active indoles from lignin

Antonio A. Castillo-Garcia, Jörg Haupenthal, Anna K. H. Hirsch and Katalin Barta*

*Correspondence to katalin.barta@uni-graz.at

1. General methods

Commercially available materials were purchased from Sigma-Aldrich or TCI Chemicals and were used as received without further purification.

Column chromatography was performed using Merck silica gel type 9385 230-400 mesh and typically pentane and ethyl acetate as eluent.

Thin layer chromatography (TLC): Merck silica gel 60, 0.25 mm. The components were visualized by UV or $KMnO_4$ staining.

Analytical methods. Product identification was performed by GC-MS (Shimadzu QP2010 Ultra) with an HP-1MS column, and helium as carrier gas. GC-MS method: The temperature program started at 50 °C for 5 min, heated by 30 °C/minute to 250 °C and held for 15 min. Conversions and product selectivity were determined by GC-FID (Agilent Technologies 6890) with an HP-5MS column using nitrogen as carrier gas. GC-FID analysis method: The temperature program started at 50 °C for 5 min, heated by 30 °C/min to 320 °C and held for 15 min. ^1H and ^{13}C NMR spectra were recorded on a Varian Mercury Plus 400 and Agilent MR 400 (400 and 101 MHz, respectively), Varian Inova 500 (500 and 126 MHz, respectively) using $CDCl_3$ as a solvent. ^1H and ^{13}C NMR spectra were recorded at room temperature. Chemical shift values are reported in ppm with the solvent resonance as the internal standard ($CDCl_3$: 7.26 for 1H, 77.00 for ^{13}C). Data are reported as follows: chemical shifts, multiplicity (s = singlet, d = doublet, t = triplet, q = quartet, br. = broad, m = multiplet), coupling constants (Hz), and integration.

1.1 Synthesis of 4-((1,3-dioxolan-2-yl)methyl)-2-methoxyphenol (**C2-G**)

C2-G was synthetized using a modified literature procedure.[1] 3.25 g N-Bromosuccinimide (0.0185 mol, NBS) and 4.75 g silver trifluoromethanesulfonate (0.0185 mol) were dispersed in 30 mL DCM and 15 mL ethylene glycol under nitrogen atmosphere. Then, 2.5 mL (0.0185 mol) of 4-vinyl guaiacol were added dropping to the mixture and let it stir for 30 minutes. The reaction was quenched adding 10 mL of water, 10 mL of a saturated $NaHCO_3$ solution and 40 mL of saturated $Na_2S_2O_3$ solution. The mixture was then extracted with DCM (20 mL x 3) and the crude mixture was purified by column chromatography (Ethyl acetate/pentane 5-30%, **G2-G** eluted at 30% Ethyl acetate/pentane). 2.1 g of **C2-G** were obtained as yellow oil (0.01 mol, 54% yield, ^1H-NMR purity: 95%).

Supplementary Note 1: The direct synthesis of protected aryl acetaldehydes has been earlier reported starting from readily available diols and vinyl arenes. These methods include the use of oxidants such as ozone and sodium peroxymonosulfate in the presence of I2 (Narender et.al Chem. Commun., 2013, 49, 1711), cupric salts and Pd catalysts (Tsuji, J. and Sridharan, V. (2004). Palladium(II) Chloride. In Encyclopedia of Reagents for Organic Synthesis, or with the use Pd-based catalysts and benzoquinone t as reported by Kataoka and coworkers (Chem. Commun., 2012, 48, 1165–1167). Nevertheless, none of the above-mentioned methods have been applied to substrates containing phenolic groups, which can be oxidized under these methods. On the other hand, the use of NBS in combination with AgOTf enables the stereoselective Anti-Markonikov dioxygenation of 4-vinyl guaiacol with ethylene glycol high selectivity and tolerance to phenolic groups, therefore, we decided to conduct the synthesis of **C2-G via** this method.

1.2 Synthesis of 2-(3,4-dimethoxybenzyl)-1,3-dioxolane (**C2-Ga**)[2]

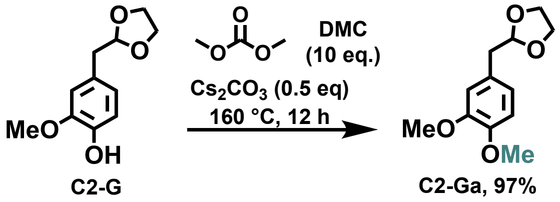

A 20 mL oven-dried microwave vial equipped with stirring bar was charged with **C2-G** (424 mg, 2.0 mmol), Cs_2CO_3 (325 mg, 0.5 eq) and dimethylcarbonate (1.8 mL, 10 eq). Then, the vial was capped and placed into a heating block at 160 °C during 16 h under continuous stirring. After reaction completion, the mixture was diluted with EtOAc (10 mL) and washed with H_2O (2x5mL). The organic phase was dried over anhydrous Na_2SO_4 and concentrated under vacuo obtaining **C2-Ga** a light-yellow solid (437 mg, 97% yield). C2-Ga was then used for the synthesis of **C2-Gb** without further purification. Characterization data are in accordance with those previously reported[2]

1.3 Synthesis of 2-(2-bromo-4,5-dimethoxybenzyl)-1,3-dioxolane (**C2-Gb**)

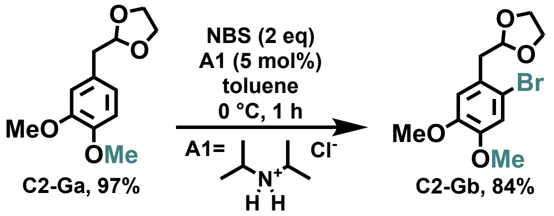

An oven-dried 10 mL glass vial was charged with **C2-Ga** (112 mg, 0.5 mmol), NBS (265 mg, 1.5 mmol), **A1** (3.5 mg, 0.025 mmol) and toluene (5 mL). Then, the mixture was stirred at 0 °C for 1 h. After reaction completion, the mixture was diluted with EtOAc (5 mL), washed with $NaHCO_3$ (2x10 mL) and saturated brine (2x10 mL). The organic phase was dried over anhydrous Na_2SO_4 and concentrated under vacuo. The residue was then purified via flash chromatography using silica gel and ethyl acetate/cyclohexane (1:1) as eluent, obtaining **C2-Gb** as a light-yellow solid (127 mg, 84% yield).

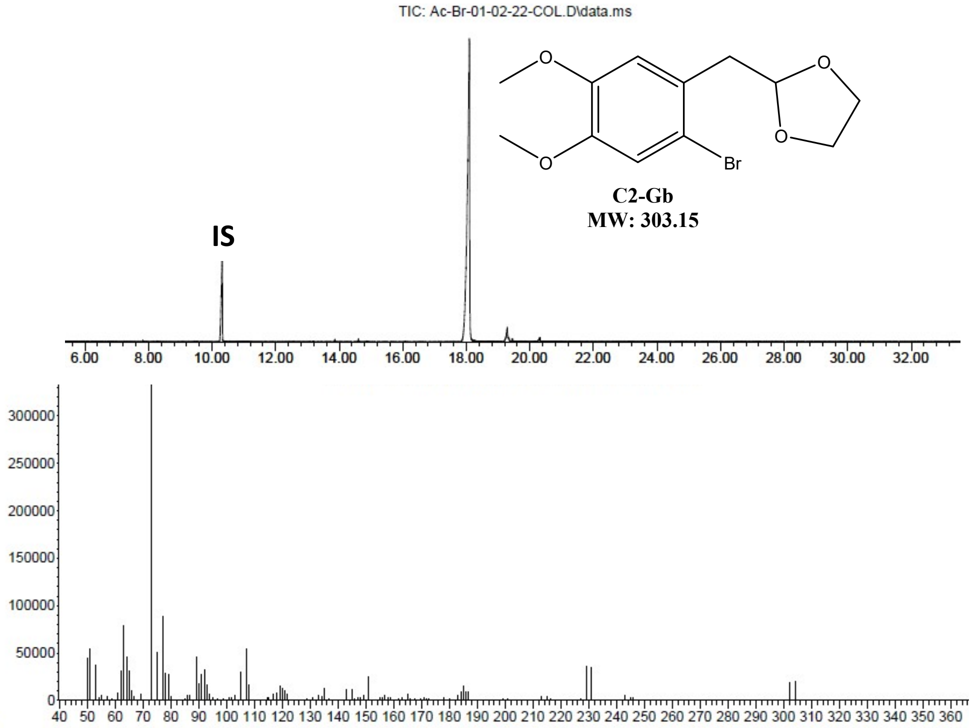

Supplementary Figure 1. GC-MS chromatogram and MS spectrum of **C2-Gb** after isolation

2. General experimental procedures

2.1 General procedure for C-N coupling of **C2-Gb** with various anilines

An oven-dried Schlenk tube equipped with stirring bar was charged with **C2-Gb** (85 mg, 0.28 mmol), amine **1** (0.3 mmol), $Pd_2(dba)_3$ ($2.8x10^{-3}$ mmol), Xantphos (5.6 x 10^{-3}mmol) and Cs_2CO_3 (0.42 mmol). Then, the Schlenk tube was subsequently connected to an argon line, toluene was added under argon stream and vacuum-argon exchange was performed three times. The Schlenk tube was capped and the mixture was rapidly stirred at room temperature for 1 min, then was placed into a pre-heated oil bath at 120 °C and stirred for 16 h. Finally, the reaction mixture was cooled down to room temperature. Conversion and yield were measured by GC-FID and GC-MS.

Table S1. C-N coupling of **C2-Gb** with aniline **(1)**: Establishing optimal reaction conditions[a]

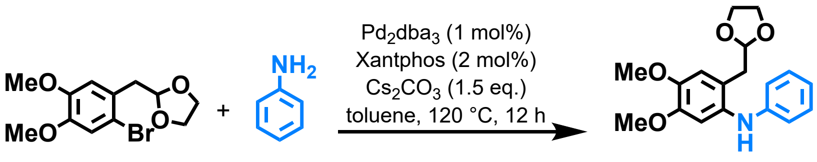

Entry	Deviation from standard conditions	C2-Gc1 (%)[b]
**1**	-	**91 (80)**[c]
**2**	$Pd(dba)_2$ as precatalyst	86
**3**	$Pd(MeCN)Cl_2$ as precatalyst	89
**4**	Dppp as ligand	0
**5**	Dppf as ligand	23
**6**	DPEPhos as ligand	35
**7**	$Ni(cod)_2$ as precatalyst	0
**8**	$Ni(dme)Cl_2$ as precatalyst	0
**9**	1 eq. of 1	76
**10**	T= 100 ˚C	56
**11**	NaOtBu instead of Cs_2CO_3	86
**12**	t= 8 h	64

[a] General reaction conditions: **C2-Gb** (0.28 mmol), **1** (0.30 mmol), Pd_2dba_3 ($2.8x10^{-3}$ mmol), Xantphos ($5.6x10^{-3}$ mmol), Cs_2CO_3 (0.42 mmol), 120 °C, 1h h. [b] Yields were determined by GC-FID. [c] Isolated yield.

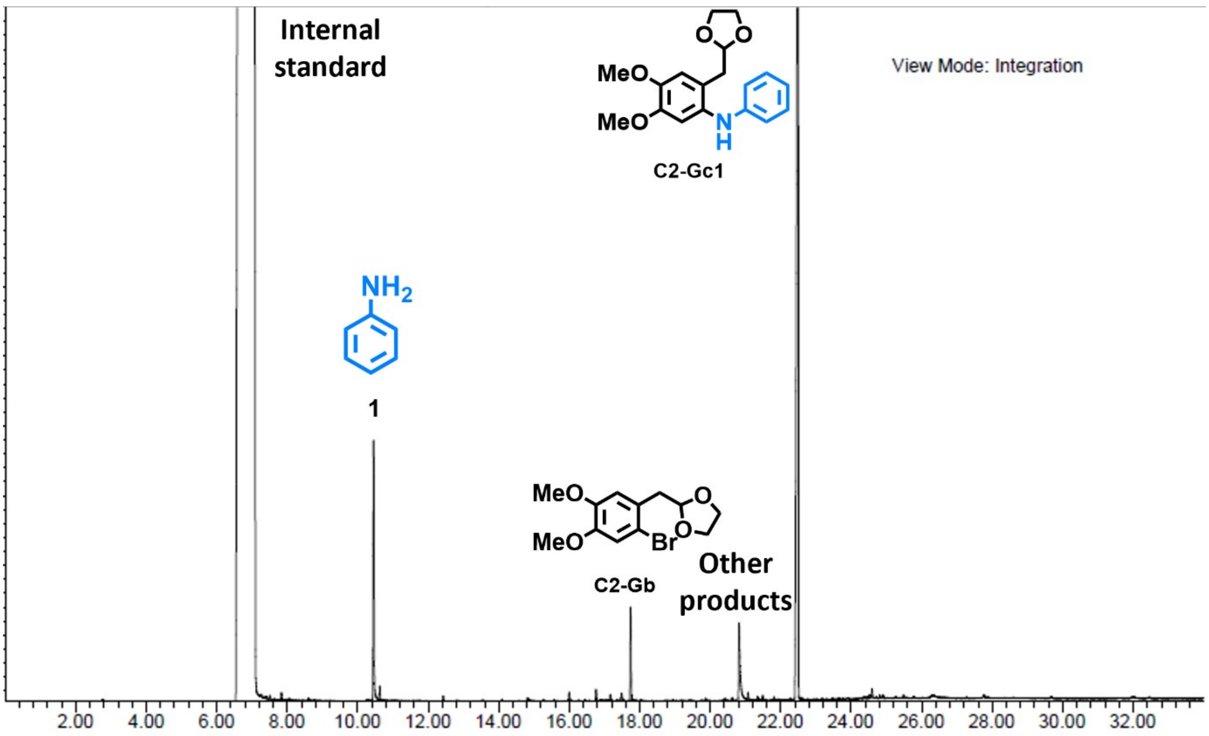

Supplementary Figure 2. GC-FID traces **Entry 1**

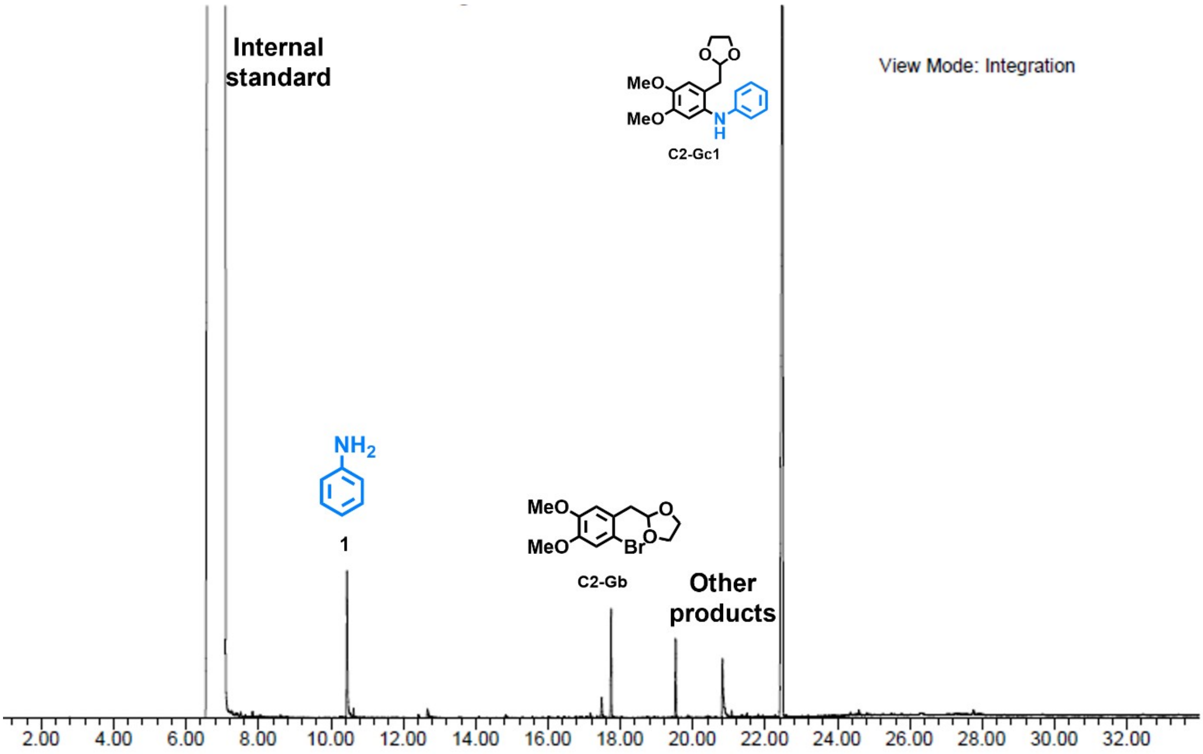

Supplementary Figure 3. GC-FID traces **Entry 3**

2.2 General procedure for the synthesis of indoles *via* acetal deprotection

A 20 mL oven-dried microwave vial equipped with stirring bar was charged with **C2-Gc$_n$** (0.15 mmol) and *p*-toluene sulfonic acid (PTSA) (0.015 mmol) in a mixture of MeOH/H_2O (1:1, 1 mL). Then, the vial was capped and placed into a heating block at 140 °C during 1 h under continuous stirring. After reaction completion, the mixture was washed with $NaHCO_3$ (2x2mL), H_2O (2 mL) and extracted with EtOAc (5 mL). The organic phase was dried over anhydrous Na_2SO_4 and concentrated under reduced pressure. The residue was purified by flash column chromatography to provide the pure indole product.

Table S2. Synthesis of **2C-Gd1** via acetal deprotection[a]

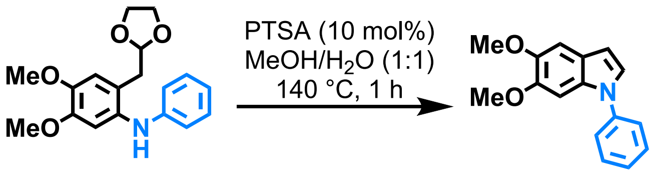

Entry	Deviation of standard conditions	Conversion (%)[a]	**C2-Gd1** (%)[b]
1	-	99	99(80)[c]
2	Only H_2O	64	58
3	Only MeOH	49	42
4	PTSA= 0.05 eq	90	85
5	T= 120 °C	69	66
6	Oxalic acid (10 mol%) as additive	41	40

[a] General reaction conditions: **C2-Gc1** (48 mg, 0.15 mmol), additive (0.05-0.1 eq), MeOH/H_2O (2 mL), 120-140 °C, 1 h. [b] Yields were determined by GC-FID. [c] Isolated yield

2.3 General procedure for the Cytotoxicity assay [3]

To obtain information regarding the cytotoxicity of our compounds, their impact on the viability of human cells was investigated. HepG2 (2 × 105 cells per well) were seeded in 24-well, flat-bottomed culture plates. Twenty-four hours after seeding the cells the incubation was started by the addition of compounds in a final DMSO concentration of 1%. Duplicates were prepared for each compound concentration. Epirubicin and doxorubicin were used as positive controls (each at 1 µM), and rifampicin was used as a negative control (at 100 µM). The living cell mass was determined 48 h after treatment with compounds by adding 0.1 volumes of 3- (4,5-dimethylthiazol-2-yl)-2,5-diphenyltetrazolium bromide (MTT) solution (5 mg per mL sterile PBS) (Sigma, St. Louis, MO) to the wells. After incubating the cells for 30 min at 37 °C (atmosphere containing 5% CO2), MTT crystals were dissolved in a solution containing 10% SDS and 0.5% acetic acid in DMSO. The optical density (OD) of the samples was determined photometrically at 570 nm in a FLUOstar Omega plate reader (BMG labtech, Ortenberg, Germany). To obtain percent inhibition values for each sample, their ODs were related to those of DMSO controls. At least two independent measurements were performed for each compound.

3. Characterization data of isolated compounds

2-(3,4-dimethoxybenzyl)-1,3-dioxolane (**C2-Ga**)

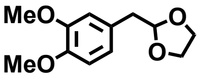

Isolated yield: 97%. **^{1}H NMR** (300 MHz, $CDCl_3$) δ 6.81 (s, 3H), 5.04 (t, J = 4.8 Hz, 1H), 4.04 – 3.90 (m, 4H), 3.88 (s, 3H), 3.85 (s, 3H), 2.91 (d, J = 4.8 Hz, 2H). **^{13}C NMR** (75 MHz, CDCl3) δ 148.83, 147.88, 128.78, 121.75, 112.94, 111.22, 104.88, 65.11, 55.96 (d, J = 2.8 Hz), 40.44. **HRMS** (ESI+ m/z) Calculated for $C_{12}H_{16}O_4$ $[M+H]^+$: 225.11177, found: 225.112135.

2-(2-bromo-4,5-dimethoxybenzyl)-1,3-dioxolane (**C2-Gb**)

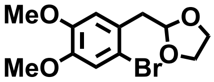

Isolated yield: 84% **^{1}H NMR** (300 MHz, $CDCl_3$) δ 7.01 (s, 1H), 6.85 (s, 1H), 5.10 (d, *J* = 4.9 Hz, 1H), 4.01 – 3.95 (m, 2H), 3.90 – 3.80 (m, 8H), 3.06 (d, *J* = 4.9 Hz, 2H). **^{13}C NMR** (75 MHz, $CDCl_3$) δ 148.59, 148.39, 127.85, 115.50, 114.92, 114.49, 103.66, 65.11, 56.23 (d, *J* = 4.9 Hz). **HRMS** (ESI^+ m/z) Calculated for $[M+H]^+$: 303.02151 , found:303.062477

2-((1,3-dioxolan-2-yl)methyl)-4,5-dimethoxy-*N*-phenylaniline (**C2Gc1**)

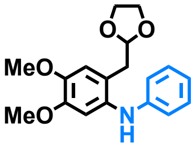

Isolated yield: 80%. **^{1}H NMR** (300 MHz, $CDCl_3$) δ 7.24 – 7.12 (m, 2H), 6.93 (s, 1H), 6.91 – 6.63 (m, 4H), 5.09 (t, *J* = 4.0 Hz, 1H), 3.95 – 3.81 (m, 7H), 3.79 (s, 3H), 2.95 (d, *J* = 4.0 Hz, 2H). **^{13}C NMR** (75 MHz, $CDCl_3$) 148.38, 145.78, 144.89, 134.99, 129.43, 120.36, 118.76, 115.17 (d, *J* = 1.6 Hz), 106.78, 105.32, 65.24, 56.36, 56.06, 36.39. **HRMS** (ESI^+ m/z) Calculated for $[M+H]^+$:315.15235 , found: 315.181464

4-((2-((1,3-dioxolan-2-yl)methyl)-4,5-dimethoxyphenyl)amino)benzonitrile (**C2-Gc2**)

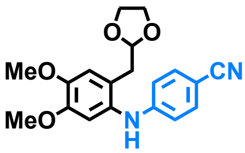

Isolated yield: 85%. **^{1}H NMR** (400 MHz, $CDCl_3$) δ ^{1}H NMR (300 MHz, $CDCl_3$) δ 7.42 (dd, *J* = 8.6, 3.4 Hz, 2H), 7.06 (s, 1H), 6.81 (d, *J* = 17.8 Hz, 1H), 6.81 – 6.71 (m, 3H), 5.08 (t, *J* = 3.8 Hz, 1H), 3.97 – 3.83 (m, 7H), 3.82 (s, 3H), 2.92 (d, *J* = 3.8 Hz, 2H). **^{13}C NMR** (75 MHz, $CDCl_3$) δ 149.79, 148.50, 146.39, 133.93 (d, *J* = 3.1 Hz), 132.21, 122.17, 120.40, 115.07, 114.56, 113.92, 108.21, 105.03, 99.92, 65.27, 56.29, 56.18, 36.19. **HRMS** (ESI^+ m/z) Calculated for $[M+H]^+$: 341.149584, found: 341.149568

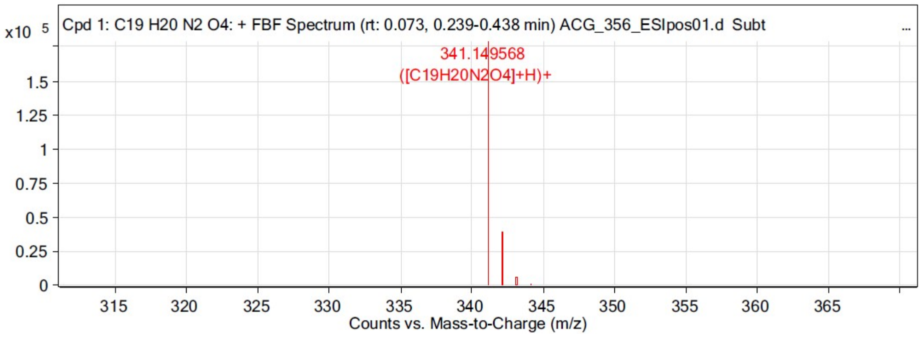

2-((1,3-dioxolan-2-yl)methyl)-4,5-dimethoxy-*N*-(4-methoxyphenyl)aniline (**C2-Gc3**)

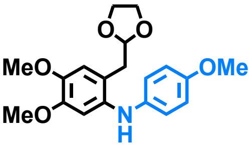

Isolated yield: 80%. **^{1}H NMR** (300 MHz, $CDCl_3$) δ 6.90 – 6.78 (m, 5H), 6.75 (s, 1H), 5.09 (t, *J* = 4.0 Hz, 1H), 3.95 – 3.88 (m, 2H), 3.85 (s, 5H), 3.76 (d, *J* = 3.2 Hz, 6H), 2.96 (d, *J* = 4.0 Hz, 2H). **^{13}C NMR** (75 MHz, $CDCl_3$) δ 153.37, 148.43, 143.94, 138.94, 136.78, 118.56, 117.99, 116.43, 115.42, 114.83, 105.31, 104.80, 65.15, 56.40, 55.93, 55.74 (d, *J* = 2.2 Hz), 36.36. **HRMS** (ESI^+ m/z) Calculated for $[M+H]^+$: 345.16121, found: 345.177187

1-(4-((2-((1,3-dioxolan-2-yl)methyl)-4,5-dimethoxyphenyl)amino)phenyl)ethan-1-one (**C2-Gc4**)

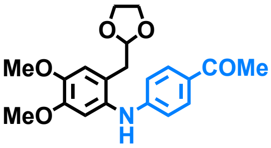

Isolated yield: 61%. **^{1}H NMR** (300 MHz, $CDCl_3$) δ 7.86 – 7.75 (m, 2H), 7.08 (s, 1H), 6.88 (s, 1H), 6.81 – 6.71 (m, 3H), 5.06 (t, *J* = 3.8 Hz, 1H), 3.90 – 3.76 (m, 10H), 2.92 (d, *J* = 3.8 Hz, 2H), 2.48 (s, 3H). **^{13}C NMR** (75 MHz, $CDCl_3$) δ 196.28, 150.29, 148.30, 145.98, 132.66, 130.79, 127.64, 121.77, 114.93, 113.14, 107.97, 104.97, 65.16, 56.19, 56.03, 36.12, 26.09. **HRMS** (ESI^+ m/z) Calculated for $[M+H]^+$: 357.16246 , found: 357.192116

2-((1,3-dioxolan-2-yl)methyl)-4,5-dimethoxy-*N*-(4-(trifluoromethyl)phenyl)aniline (**C2-Gc5**)

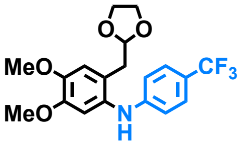

Isolated yield: 98%. **^{1}H NMR** (400 MHz, $CDCl_3$) δ ^{1}H NMR (300 MHz, $CDCl_3$) δ 7.41 (d, *J* = 8.4 Hz, 2H), 6.88 (s, 1H), 6.84 – 6.72 (m, 3H), 5.09 (t, *J* = 3.8 Hz, 1H) 3.89 (s, 7H), 3.81 (s, 3H), 2.94 (d, *J* = 3.8 Hz, 2H).^{13}C NMR (75 MHz, $CDCl_3$) δ **^{13}C NMR** (75 MHz, $CDCl_3$) δ 148.92, 148.47, 145.91, 133.25, 126.80 (d, *J* = 3.9 Hz), 121.68, 115.07, 113.72, 107.88, 105.15, 65.28, 56.31, 56.14. **HRMS** (ESI^+ m/z) Calculated for $[M+H]^+$: 384.140053 , found: 384.171533

2-((1,3-dioxolan-2-yl)methyl)-4,5-dimethoxy-*N*-(3-(trifluoromethyl)phenyl)aniline (**C2-Gc6**)

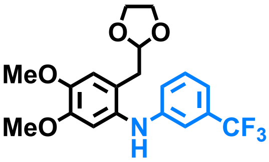

Isolated yield: 83%. **^{1}H NMR** (400 MHz, $CDCl_3$) δ 7.07 – 6.95 (m, 3H), 6.99 – 6.78 (m, 1H), 6.78 (s, 2H), 5.09 (d, *J* = 3.8 Hz, 1H), 3.88 (d, *J* = 3.8 Hz, 7H), 3.80 (s, 3H), 2.95 (d, *J* = 3.9 Hz, 2H). **^{13}C NMR** (75 MHz, $CDCl_3$) δ 148.49, 146.36, 145.57, 133.77, 129.85, 121.13, 117.70, 115.20, 114.91 (d, *J* = 4.0 Hz), 111.12 (d, *J* = 3.8 Hz), 107.14, 105.20, 65.28, 56.33, 56.08, 36.28. **HRMS** (ESI^+ m/z) Calculated for $[M+H]^+$: 384.140053, found: 384.162393

2-((1,3-dioxolan-2-yl)methyl)-4,5-dimethoxy-*N*-(4-(methylthio)phenyl)aniline (**C2-Gc7**)

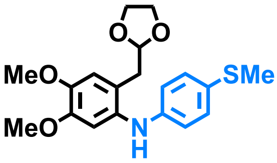

Isolated yield: 65%. **^{1}H NMR** (300 MHz, $CDCl_3$) δ 7.23 – 7.13 (m, 2H), 6.88 (s, 1H), 6.84 – 6.73 (m, 3H), 5.08 (d, *J* = 3.9 Hz, 1H), 3.96 – 3.82 (m, 7H), 3.79 (s, 3H), 2.94 (d, *J* = 4.0 Hz, 2H), 2.43 (s, 3H). **^{13}C NMR** (75 MHz, $CDCl_3$) δ 148.42, 145.03, 144.48, 134.73, 131.05, 125.97, 120.41, 115.82, 115.17, 106.73, 105.29, 65.25, 56.36, 56.09, 36.37, 18.82. **HRMS** (ESI^+ m/z) Calculated for $[M+H]^+$: 361.13284, found:361.164366

Methyl 4-((2-((1,3-dioxolan-2-yl)methyl)-4,5-dimethoxyphenyl)amino)benzoate (**C2-Gc8**)

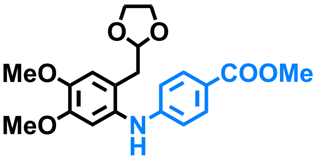

Isolated yield: 73%. **^{1}H NMR** (300 MHz, $CDCl_3$) δ 7.92 – 7.80 (m, 2H), 7.00 (s, 1H), 6.90 (s, 1H), 6.83 – 6.72 (m, 3H), 5.09 (t, *J* = 3.8 Hz, 1H), 3.90 – 3.83 (m, 7H), 3.81 (s, 3H), 2.94 (d, *J* = 3.8 Hz, 2H), 1.59 (s, 3H).**^{13}C NMR** (75 MHz, $CDCl_3$) δ 167.31, 150.12, 148.43, 145.95, 133.06, 131.69, 121.65, 119.56, 115.06, 113.38, 107.93, 105.16, 65.28, 56.32, 56.14, 51.76, 36.28. **HRMS** (ESI^+ m/z) Calculated for $[M+H]^+$: 374.159814, found: 374.159583

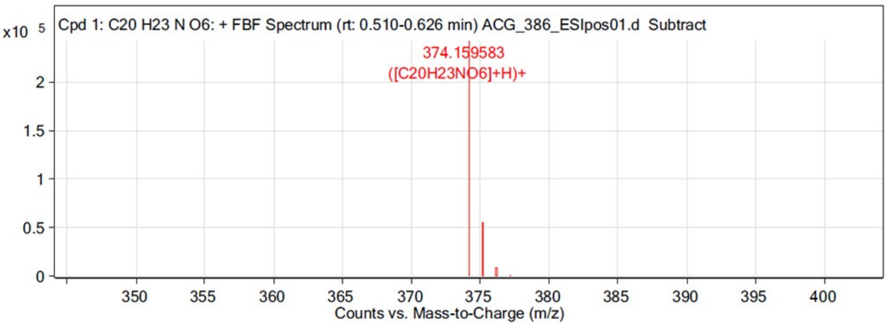

2-((1,3-dioxolan-2-yl)methyl)-*N*-(4-fluorophenyl)-4,5-dimethoxyaniline (**C2-Gc9**)

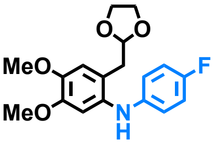

Isolated yield: 90%. **^{1}H NMR** (300 MHz, $CDCl_3$) δ 6.89 (d, *J* = 8.5 Hz, 2H), 6.85 – 6.69 (m, 4H), 5.08 (t, *J* = 4.0 Hz, 1H), 3.88 (d, *J* = 5.9 Hz, 7H), 3.78 (s, 3H), 2.94 (d, *J* = 4.0 Hz, 2H). **^{13}C NMR** (75 MHz, $CDCl_3$) δ 148.52, 144.75, 141.92, 135.71, 119.88, 116.80 (d, *J* = 7.5 Hz), 116.04, 115.75, 115.34, 106.07, 105.32, 65.26, 56.42, 56.06, 36.38. **HRMS** (ESI^+ m/z) Calculated for $[M+H]^+$: 334.144913, found: 334.145041.

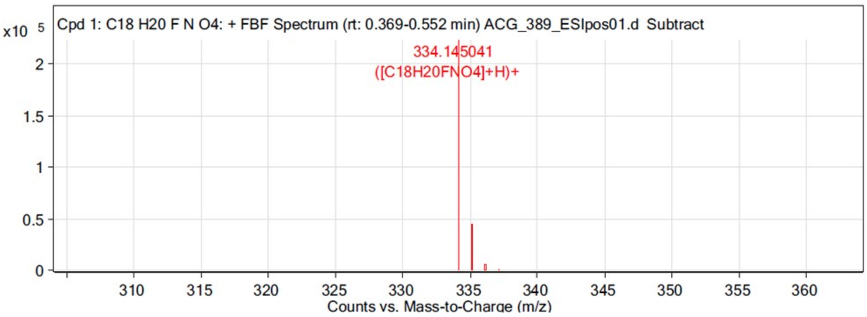

2-((1,3-dioxolan-2-yl)methyl)-*N*-(3-fluorophenyl)-4,5-dimethoxyaniline (**C2-Gc10**)

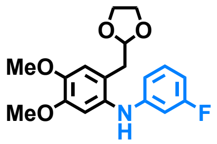

Isolated yield: 88%. ^{1}**H NMR** (300 MHz, $CDCl_3$) δ 7.11 (td, *J* = 8.1, 6.7 Hz, 1H), 6.89 (s, 1H), 6.77 (s, 1H), 6.61 – 6.38 (m, 3H), 5.08 (t, *J* = 3.9 Hz, 1H), 3.87 (d, *J* = 3.7 Hz, 7H), 3.81 (s, 3H), 2.93 (d, *J* = 3.9 Hz, 2H). ^{13}**C NMR** (75 MHz, $CDCl_3$) δ 148.45, 147.88, 145.53, 134.03, 130.50 (d, *J* = 10.2 Hz), 121.17, 115.12, 110.53 (d, *J* = 2.4 Hz), 107.63, 105.20 (d, *J* = 3.0 Hz), 104.89, 101.61, 101.28, 65.26, 56.34, 56.13, 36.32. **HRMS** (ESI^+ m/z) Calculated for $[M+H]^+$: 334.144913, found: 334.144903.

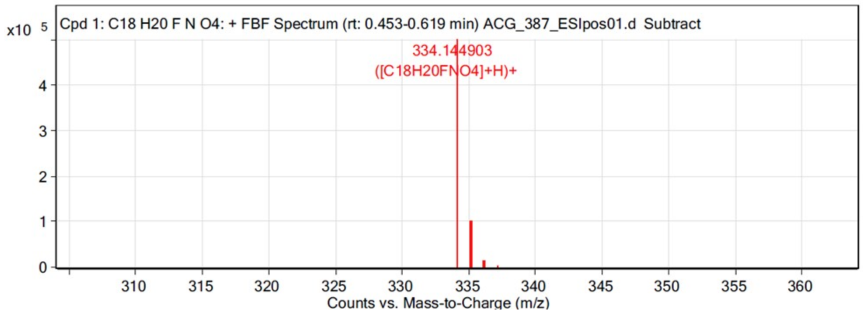

2-((1,3-dioxolan-2-yl)methyl)-4,5-dimethoxy-*N*-(4-nitrophenyl)aniline (**C2-Gc11**)

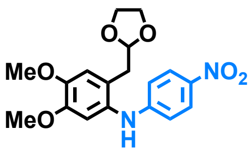

Isolated yield: 85%. ^{1}**H NMR** (300 MHz, $CDCl_3$) δ 8.14 – 8.02 (m, 2H), 7.35 (s, 1H), 6.86 (s, 1H), 6.82 – 6.69 (m, 3H), 5.09 (t, *J* = 3.7 Hz, 1H), 3.90 (s, 4H), 3.87 (s, 3H), 3.83 (s, 3H), 2.94 (d, *J* = 3.7 Hz, 2H). ^{13}**C NMR** (75 MHz, $CDCl_3$) δ 151.82, 148.57, 146.72, 138.87, 131.80, 126.56, 122.35, 115.11, 112.85, 108.31, 104.99, 65.31, 56.27 (d, *J* = 6.6 Hz), 36.18. **HRMS** (ESI^+ m/z) Calculated for $[M+H]^+$: 360.132136, found: 360.132182.

2-((1,3-dioxolan-2-yl)methyl)-*N*-(4-ethylphenyl)-4,5-dimethoxyaniline (**C2-Gc12**)

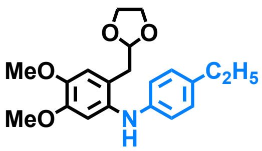

Isolated yield: 62%. ^{1}**H NMR** (300 MHz, $CDCl_3$) δ 7.31 – 7.27 (m, 1H), 6.91 (d, *J* = 3.5 Hz, 1H), 6.85 – 6.72 (m, 3H), 6.78 – 6.56 (m, 1H), 5.56 (dd, *J* = 17.6, 1.1 Hz, 1H), 5.13 – 4.92 (m, 2H), 3.87 (q, *J* = 3.2, 2.7 Hz, 7H), 3.79 (s, 3H), 2.94 (t, *J* = 5.0 Hz, 2H). ^{13}**C NMR** (75 MHz, $CDCl_3$) δ 148.40, 145.52, 145.02, 136.62, 134.70, 128.52, 127.48, 120.43, 115.10 (d, *J* = 9.8 Hz), 110.16, 106.84, 105.31, 65.26, 56.37, 56.08, 36.37. **HRMS** (ESI^+ m/z) Calculated for $[M+H]^+$: 374.159814, found: 374.159529

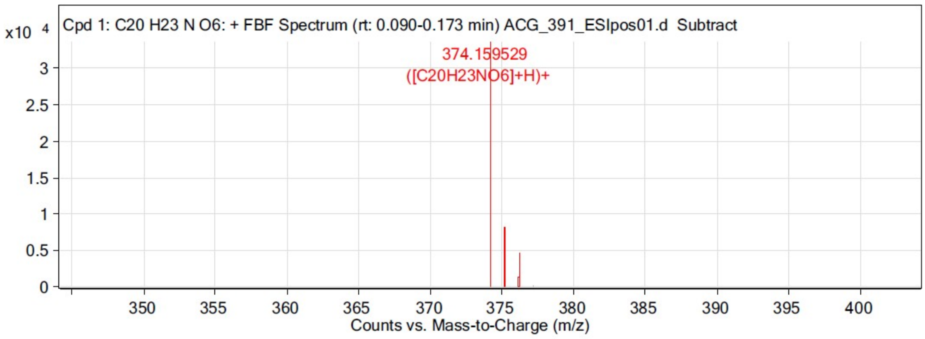

2-((1,3-dioxolan-2-yl)methyl)-4,5-dimethoxy-*N*-(4-methoxybenzyl)aniline (**C2-Gc13**)

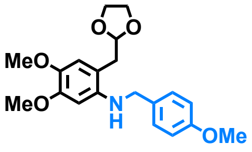

Isolated yield: 63%. **^{1}H NMR** (300 MHz, $CDCl_3$) δ 7.31 (d, *J* = 8.5 Hz, 2H), 6.87 (dd, *J* = 9.1, 2.6 Hz, 2H), 6.69 (s, 1H), 6.32 (s, 1H), 5.02 (t, *J* = 4.5 Hz, 1H), 4.24 (s, 2H), 3.87 (dddd, *J* = 11.0, 7.9, 5.9, 2.9 Hz, 4H), 3.80 (d, *J* = 1.0 Hz, 6H), 3.78 (s, 3H), 2.88 (d, *J* = 4.4 Hz, 2H). **^{13}C NMR** (75 MHz, $CDCl_3$) δ 158.82, 148.99, 142.06, 140.87, 131.94, 128.90, 116.78, 114.28, 114.05, 112.79, 105.27, 98.07, 65.12, 57.03, 55.99, 55.41, 48.67, 36.57. **HRMS** (ESI$^+$ m/z) Calculated for $[M+H]^+$: 360.180549, found: 360.180566.

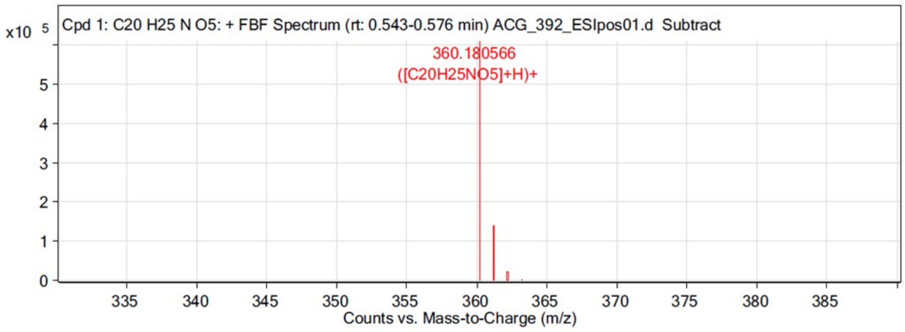

2-((1,3-dioxolan-2-yl)methyl)-*N*-(3,4-dimethoxybenzyl)-4,5-dimethoxyaniline (**C2-Gc14**)

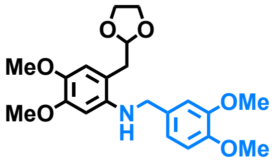

Isolated yield: 51%. **^{1}H NMR** (300 MHz, $CDCl_3$) δ 6.98 – 6.89 (m, 2H), 6.88 – 6.79 (m, 1H), 6.69 (s, 1H), 6.33 (s, 1H), 5.02 (t, *J* = 4.4 Hz, 1H), 4.24 (s, 2H), 3.87 (d, *J* = 3.2 Hz, 8H), 3.79 (d, *J* = 5.3 Hz, 8H), 2.89 (d, *J* = 4.4 Hz, 2H). **^{13}C NMR** (75 MHz, $CDCl_3$) δ 149.00, 148.21, 142.03, 140.97, 132.50, 119.82, 116.73, 112.85, 111.26, 111.01, 105.34, 98.14, 65.14, 57.02, 56.08 – 55.84 (m), 49.10, 36.60. **HRMS** (ESI$^+$ m/z) Calculated for $[M+H]^+$: 390.191114, found: 390.191109.

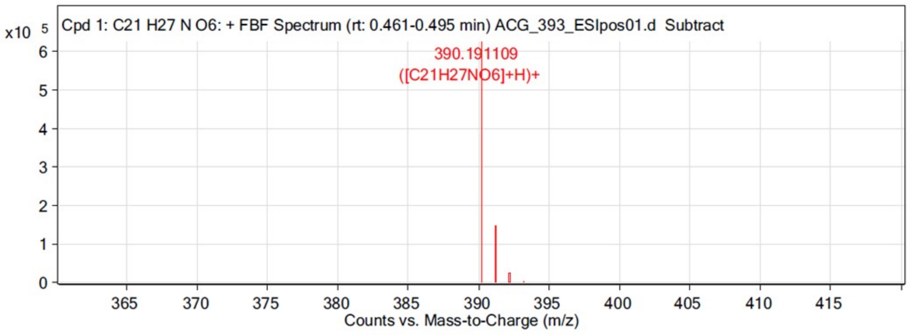

2-((1,3-dioxolan-2-yl)methyl)-4,5-dimethoxy-*N*-(4-(trifluoromethyl)benzyl)aniline (**C2-Gc15**)

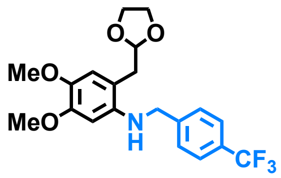

Isolated yield: 54%. **^{1}H NMR** (300 MHz, $CDCl_3$) δ 7.62 (d, *J* = 8.2 Hz, 1H), 7.53 (d, *J* = 8.1 Hz, 1H), 6.73 (s, 0H), 6.23 (s, 0H), 5.06 (t, *J* = 4.3 Hz, 0H), 4.42 (s, 1H), 4.02 – 3.81 (m, 4H), 3.81 (d, *J* = 2.1 Hz, 0H), 3.75 (s, 1H), 2.96 (d, *J* = 4.4 Hz, 1H). **^{13}C NMR** (75 MHz, $CDCl_3$) δ 148.99, 144.31, 141.42, 141.21, 127.69, 125.62 (t, *J* = 3.8 Hz), 116.93, 113.11, 105.35, 98.16, 65.14, 56.97, 55.98, 48.58, 36.59. **HRMS** (ESI$^+$ m/z) Calculated for $[M+H]^+$: 384.141719, found: 384.141560.

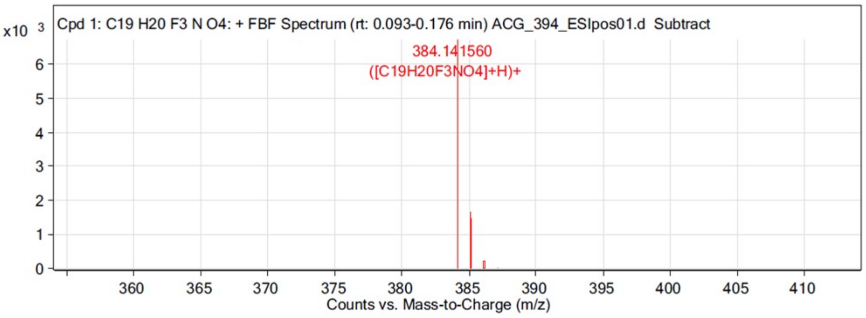

5,6-dimethoxy-1-phenyl-1*H*-indole (**C2-Gd1**)

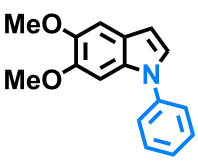

Isolated yield: 80%. **^{1}H NMR** (300 MHz, $CDCl_3$) δ 7.59 – 7.45 (m, 4H), 7.36 (tt, *J* = 5.8, 2.2 Hz, 1H), 7.21 (d, *J* = 3.2 Hz, 1H), 7.13 (s, 1H), 7.06 (s, 1H), 6.61 – 6.53 (m, 1H), 3.95 (s, 3H), 3.88 (s, 3H). **^{13}C NMR** (75 MHz, $CDCl_3$) δ 147.33, 145.62, 140.21, 130.34, 129.84, 126.61, 126.43, 124.21, 122.13, 103.30, 102.65, 94.12, 56.44, 56.40. **HRMS** (ESI$^+$ m/z) Calculated for $[M+H]^+$: 254.117555, found 254.118006.

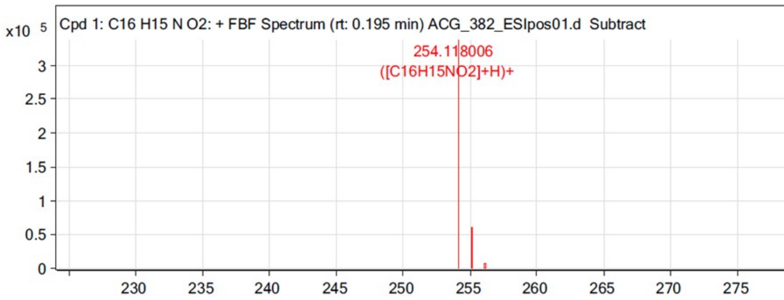

4-(5,6-dimethoxy-1*H*-indol-1-yl)benzonitrile (**C2-Gd2**)

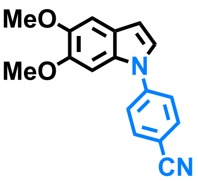

Isolated yield: 92%. A red solid was obtained after column chromatography (SiO_2, Pentane/EtOAc 70:30). **^{1}H NMR** (300 MHz, $CDCl_3$) δ 7.45 – 7.34 (m, 2H), 7.18 – 7.10 (m, 2H), 7.10 – 6.99 (m, 2H), 6.97 – 6.91 (m, 1H), 6.54 (dd, *J* = 3.2, 0.8 Hz, 1H), 3.95 (s, 3H), 3.88 (s, 3H), 3.87 (s, 3H). **^{13}C NMR** (75 MHz, $CDCl_3$) δ 158.27, 147.26, 145.45, 133.18, 130.87, 126.93,125.90, 121.67, 114.91, 102.56, 93.86, 56.44, 56.34, 55.70. **HRMS** (ESI$^+$ m/z). Calculated for $[M+H]^+$: 279.112947, found: 279.112947.

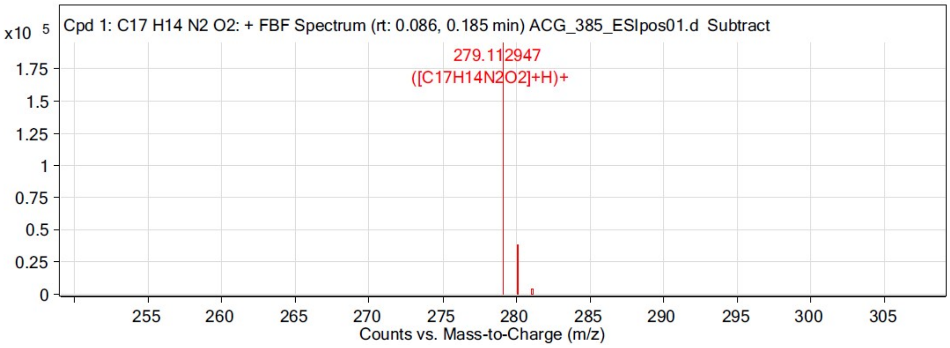

5,6-dimethoxy-1-(4-methoxyphenyl)-1*H*-indole (**C2-Gd3**)

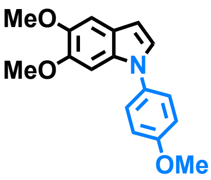

Isolated yield: 73%. **^{1}H NMR** δ 7.86 – 7.76 (m, 2H), 7.67 – 7.56 (m, 2H), 7.20 (d, *J* = 3.3 Hz, 1H), 7.10 (d, *J* = 9.9 Hz, 2H), 6.69 – 6.58 (m, 1H), 3.94 (s, 3H), 3.90 (s, 3H). **^{13}C NMR** (75 MHz, $CDCl_3$) δ 147.73, 146.16, 143.92, 133.96 (d, *J* = 7.2 Hz), 129.57, 125.73, 123.61, 122.90, 118.58, 114.52, 109.21, 105.54, 102.96, 94.13, 56.46, 56.37. **HRMS** (ESI$^+$ m/z). Calculated for $[M+H]^+$: 284.122135 , found: 284.114467.

5,6-dimethoxy-1-(4-(trifluoromethyl)phenyl)-1*H*-indole (**C2-Gd5**)

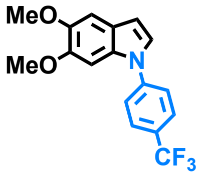

Isolated yield: 68%. **^{1}H NMR** (300 MHz, $CDCl_3$) δ 7.79 (d, *J* = 8.3 Hz, 2H), 7.62 (d, *J* = 8.3 Hz, 2H), 7.21 (d, *J* = 3.3 Hz, 1H), 7.10 (d, *J* = 15.4 Hz, 2H), 6.61 (d, *J* = 3.3 Hz, 1H), 3.95 (s, 3H), 3.90 (s, 3H). **^{13}C NMR** (75 MHz, $CDCl_3$) δ 147.52, 145.85, 143.06, 129.80, 127.76, 126.97 (d, *J* = 3.8 Hz), 125.95, 123.60, 122.46, 104.56, 102.73, 93.89, 56.30 (d, *J* = 1.4 Hz). **HRMS** (ESI$^+$ m/z). Calculated for $[M+H]^+$: 322.10494, found: 322.105008.

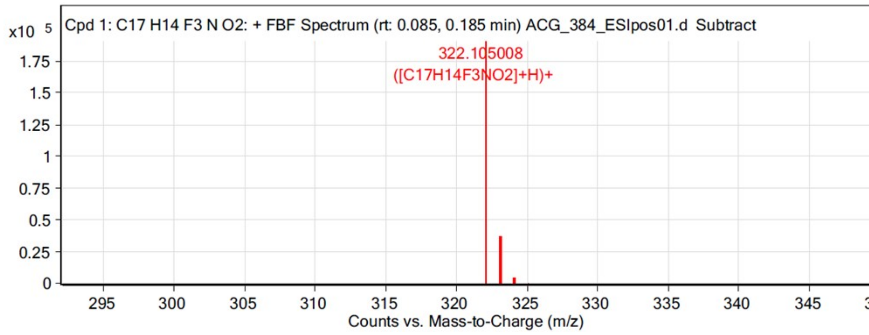

5,6-dimethoxy-1-(3-(trifluoromethyl)phenyl)-1*H*-indole (**C2-Gd6**)

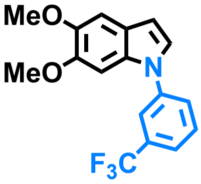

Isolated yield: 76%. **^{1}H NMR** (300 MHz, $CDCl_3$) δ 7.79 (d, *J* = 8.3 Hz, 2H), 7.62 (d, *J* = 8.3 Hz, 2H), 7.21 (d, *J* = 3.3 Hz, 1H), 7.10 (d, *J* = 15.4 Hz, 2H), 6.61 (d, *J* = 3.3 Hz, 1H), 3.95 (s, 3H), 3.90 (s, 3H). **^{13}C NMR** (75 MHz, $CDCl_3$) δ 147.52, 145.85, 143.06, 129.80, 127.76, 126.97 (d, *J* = 3.8 Hz), 125.95, 123.60, 122.46, 104.56, 102.73, 93.89, 56.30 (d, *J* = 1.4 Hz). **HRMS** (ESI$^+$ m/z). Calculated for $[M+H]^+$: 322.115758, found: 322.115838.

5,6-dimethoxy-1-(4-(methylthio)phenyl)-1*H*-indole (**C2-Gd7**)

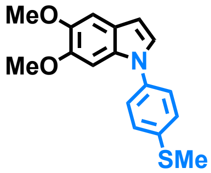

Isolated yield: 80%. **^{1}H NMR** (300 MHz, $CDCl_3$) δ 7.41 (s, 4H), 7.17 (d, *J* = 3.2 Hz, 1H), 7.12 (s, 1H), 7.00 (s, 1H), 6.59 – 6.52 (m, 1H), 3.95 (s, 3H), 3.88 (s, 3H), 2.55 (s, 3H).**^{13}C NMR** (75 MHz, $CDCl_3$) δ 147.35, 145.60, 137.40, 136.64, 130.39, 127.95, 126.49, 124.73, 122.01, 103.28, 102.66, 93.97, 56.40 (d, *J* = 4.7 Hz), 16.27. **HRMS** (ESI$^+$ m/z). Calculated for $[M+H]^+$: 300.105276, found: 300.104920.

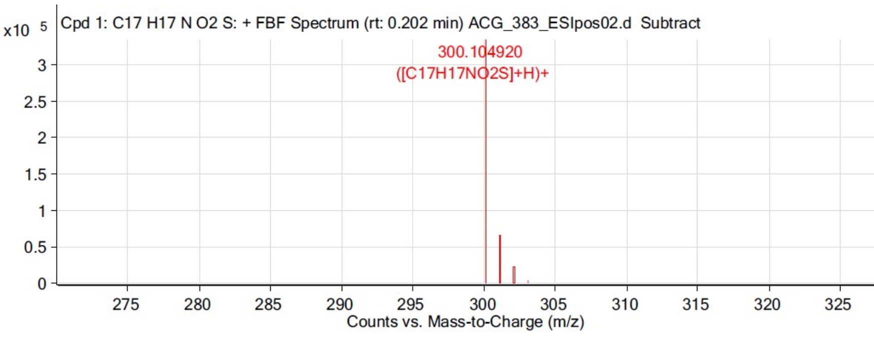

Methyl 4-(5,6-dimethoxy-1*H*-indol-1-yl)benzoate (**C2-Gd8**)

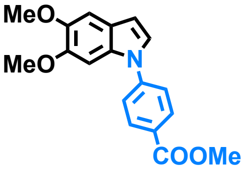

Isolated yield: 69%. **^{1}H NMR** δ 7.86 – 7.76 (m, 2H), 7.67 – 7.56 (m, 2H), 7.20 (d, *J* = 3.3 Hz, 1H), 7.10 (d, *J* = 9.9 Hz, 2H), 6.69 – 6.58 (m, 1H), 3.94 (s, 3H), 3.90 (s, 3H). **^{13}C NMR** (75 MHz, $CDCl_3$) δ 147.73, 146.16, 143.92, 133.96 (d, *J* = 7.2 Hz), 129.57, 125.73, 123.61, 122.90, 118.58, 114.52, 109.21, 105.54, 102.96, 94.13, 56.46, 56.37. **HRMS** (ESI$^+$ m/z). Calculated for $[M+H]^+$: 311.113313, found: 311.113684.

1-(4-fluorophenyl)-5,6-dimethoxy-1*H*-indole (**C2-Gd9**)

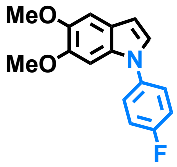

Isolated yield: 66%. ^{1}**H NMR** (300 MHz, $CDCl_3$ δ 7.53 – 7.40 (m, 2H), 7.31 – 7.18 (m, 2H), 7.19 – 7.09 (m, 2H), 6.96 (s, 1H), 6.58 (d, *J* = 3.0 Hz, 1H), 3.96 (s, 3H), 3.89 (s, 3H). ^{13}**C NMR** (75 MHz, $CDCl_3$) δ 162.75, 159.49, 147.44, 145.65, 136.26, 130.61, 126.67, 126.14, 121.92, 116.82, 116.52, 103.29, 102.67, 93.75, 56.41 (d, *J* = 3.7 Hz). **HRMS** (ESI^{+} m/z). Calculated for $[M+H]^{+}$:271.11161 , found:271.174021

1-(3-fluorophenyl)-5,6-dimethoxy-1*H*-indole (**C2-Gd10**)

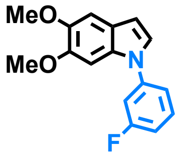

Isolated yield: 88%. ^{1}**H NMR** (300 MHz, $CDCl_3$) δ 7.48 (td, *J* = 8.2, 6.3 Hz, 1H), 7.30 (ddd, *J* = 7.9, 2.1, 1.0 Hz, 1H), 7.23 (dt, *J* = 9.7, 2.3 Hz, 1H), 7.19 (d, *J* = 3.3 Hz, 1H), 7.12 (s, 1H), 7.09 – 7.04 (m, 3H), 6.61 – 6.54 (m, 1H), 3.95 (s, 3H), 3.90 (s, 3H).^{13}**C NMR** (75 MHz, $CDCl_3$) δ 147.52, 145.82, 131.08 (d, *J* = 9.7 Hz), 130.07, 126.31, 122.34, 119.54, 113.37, 113.10, 111.49, 111.17, 104.04, 102.76, 94.07, 56.43. **HRMS** (ESI^{+} m/z). Calculated for $[M+H]^{+}$: 271.097663, found: 271.097845

5,6-dimethoxy-1-(4-nitrophenyl)-1*H*-indole (**C2-Gd11**)

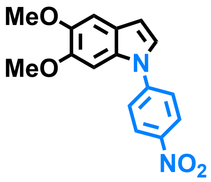

Isolated yield: 85%. ^{1}**H NMR** (300 MHz, $CDCl_3$) δ 8.48 – 8.37 (m, 2H), 7.74 – 7.61 (m, 2H), 7.31 – 7.23 (m, 2H), 7.14 (s, 1H), 6.67 (d, *J* = 3.3 Hz, 1H), 3.97 (s, 3H), 3.93 (s, 3H).^{13}**C NMR** (75 MHz, $CDCl_3$) δ 147.84, 146.30, 145.61, 145.00, 129.62, 125.74, 123.06, 106.02, 103.03, 94.26, 56.48, 56.38. **HRMS** (ESI^{+} m/z). Calculated for $[M+H]^{+}$: 298.10406 , found:298.312204

5,6-dimethoxy-1-(4-methoxybenzyl)-1*H*-indole (**C2-Gd13**)

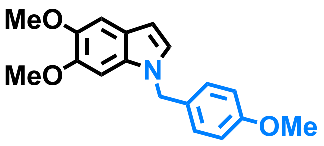

Isolated yield: 69% ^{1}**H NMR** (300 MHz, $CDCl_3$) δ 7.09 (s, 1H), 7.08 – 7.02 (m, 2H), 6.98 (d, *J* = 3.1 Hz, 1H), 6.93 – 6.76 (m, 2H), 6.74 (d, *J* = 0.8 Hz, 1H), 6.42 (dd, *J* = 3.1, 0.8 Hz, 1H), 5.20 (s, 2H), 3.91 (s, 3H), 3.86 (s, 3H), 3.78 (s, 4H). ^{13}**C NMR** (75 MHz, $CDCl_3$) δ 159.20, 146.97, 145.14, 129.68, 128.24, 126.73, 121.47, 114.27, 102.69, 101.18, 93.39, 56.43, 55.43, 49.89, 29.85. **HRMS** (ESI^{+} m/z). Calculated for $[M+H]^{+}$: 297.14631 , found: 297.191245

1-(3,4-dimethoxybenzyl)-5,6-dimethoxy-1H-indole (**C2-Gd14**)

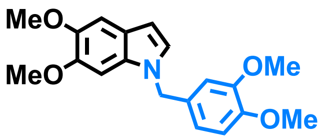

Isolated yield: 60%. ^{1}**H NMR** (300 MHz, $CDCl_3$) δ 7.09 (s, 1H), 6.98 (d, *J* = 3.1 Hz, 1H), 6.86 – 6.71 (m, 2H), 6.71 – 6.62 (m, 2H), 6.42 (dd, *J* = 3.1, 0.8 Hz, 1H), 5.20 (s, 2H), 3.92 (s, 3H), 3.86 (s, 4H), 3.85 (s, 4H), 3.78 (s, 3H). **HRMS** (ESI^{+} m/z). Calculated for $[M+H]^{+}$: 327.15485 , found: 327.202572

4. NMR Characterization

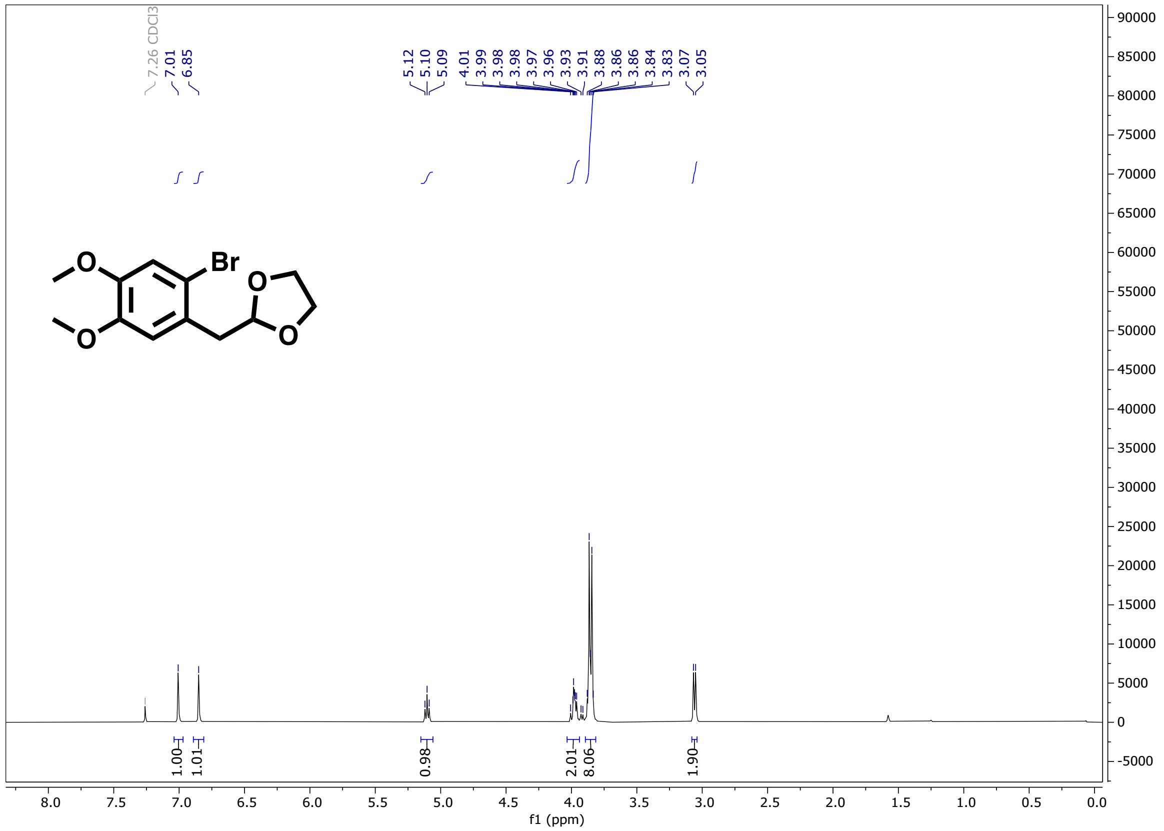

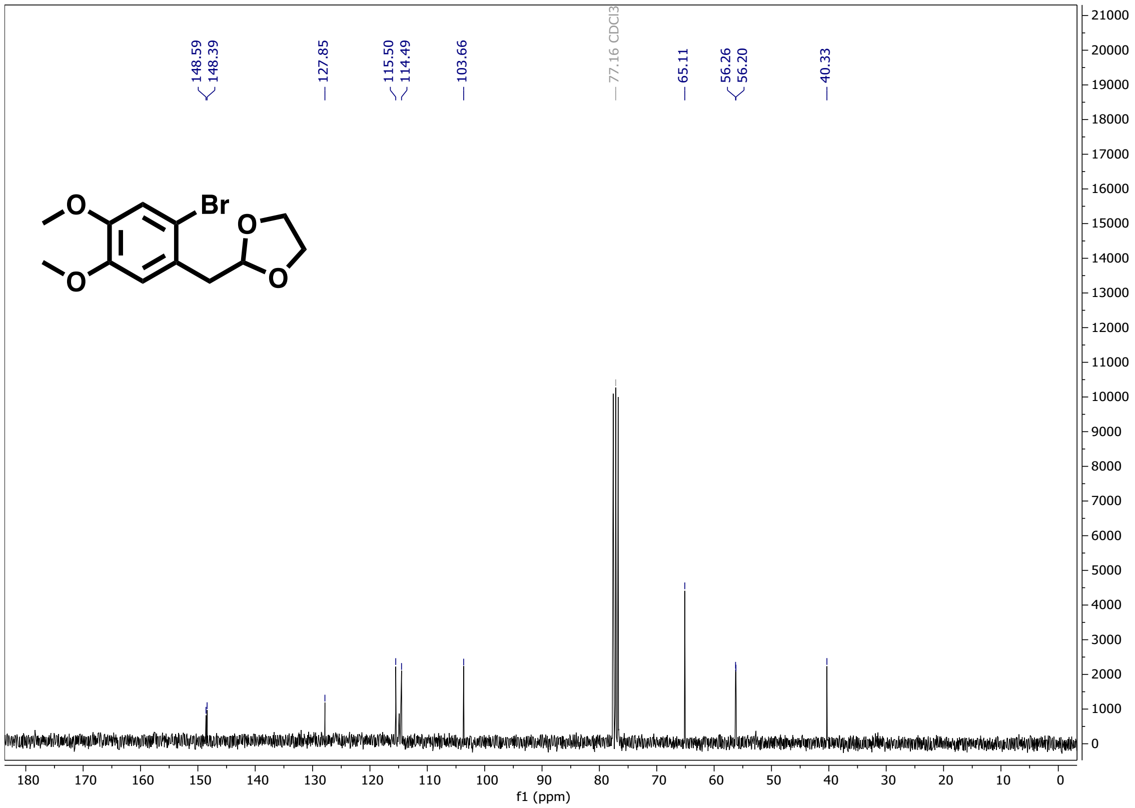

Supplementary Figure 4. ^{1}H and ^{13}C spectra of compound **C2-Gb**

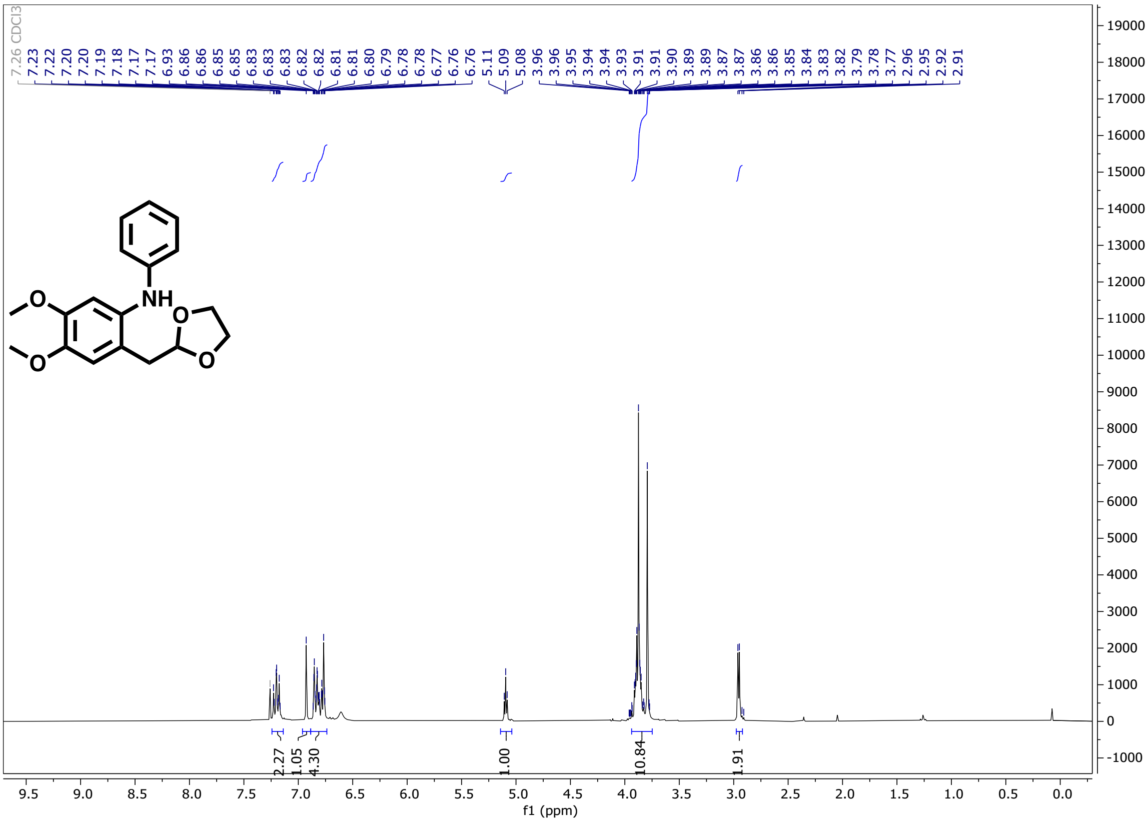

7.26 CDCl3
7.23 7.22 7.20 7.20 7.19 7.18 7.17 7.17 6.93 6.86 6.85 6.85 6.83 6.83 6.83 6.82 6.82 6.81 6.81 6.80 6.79 6.78 6.78 6.77 6.76 6.76
5.11 5.09 5.08
3.96 3.96 3.95 3.94 3.94 3.93 3.91 3.91 3.90 3.89 3.89 3.87 3.87 3.86 3.86 3.85 3.84 3.83 3.82 3.79 3.78 3.77 2.96 2.95 2.92 2.91
NH
O
2.27
1.05
4.30
1.00
10.84
1.91
f1 (ppm)

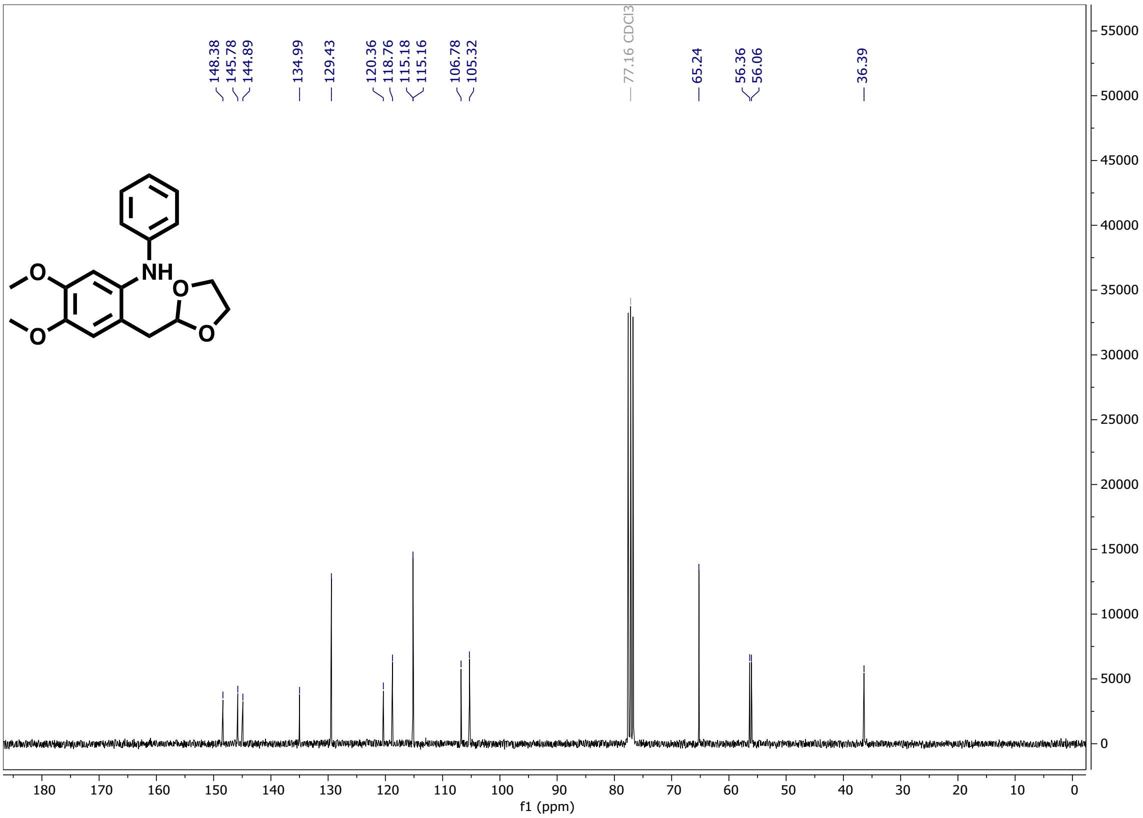

Supplementary Figure 5. ^{1}H and ^{13}C spectra of compound **C2-Gc1**

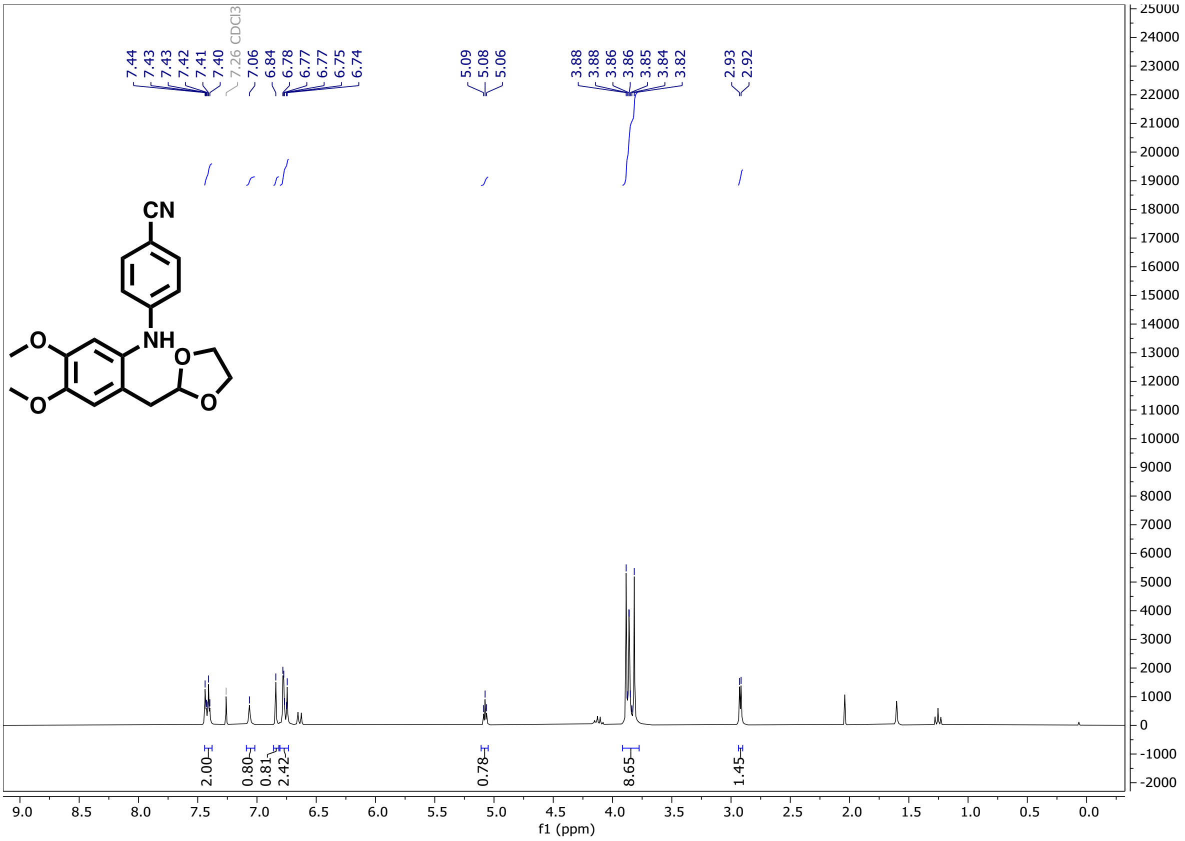

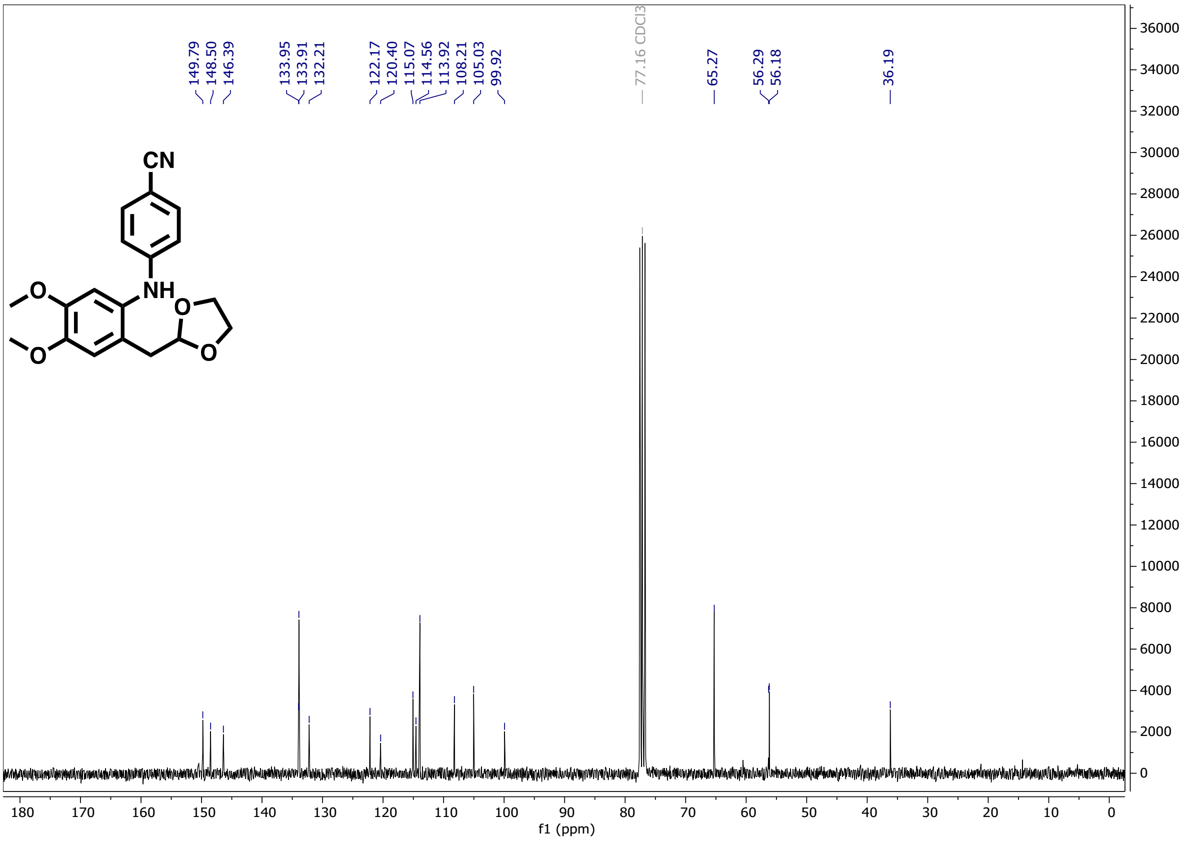

Supplementary Figure 6. ^{1}H and ^{13}C spectra of compound **C2-Gc2**

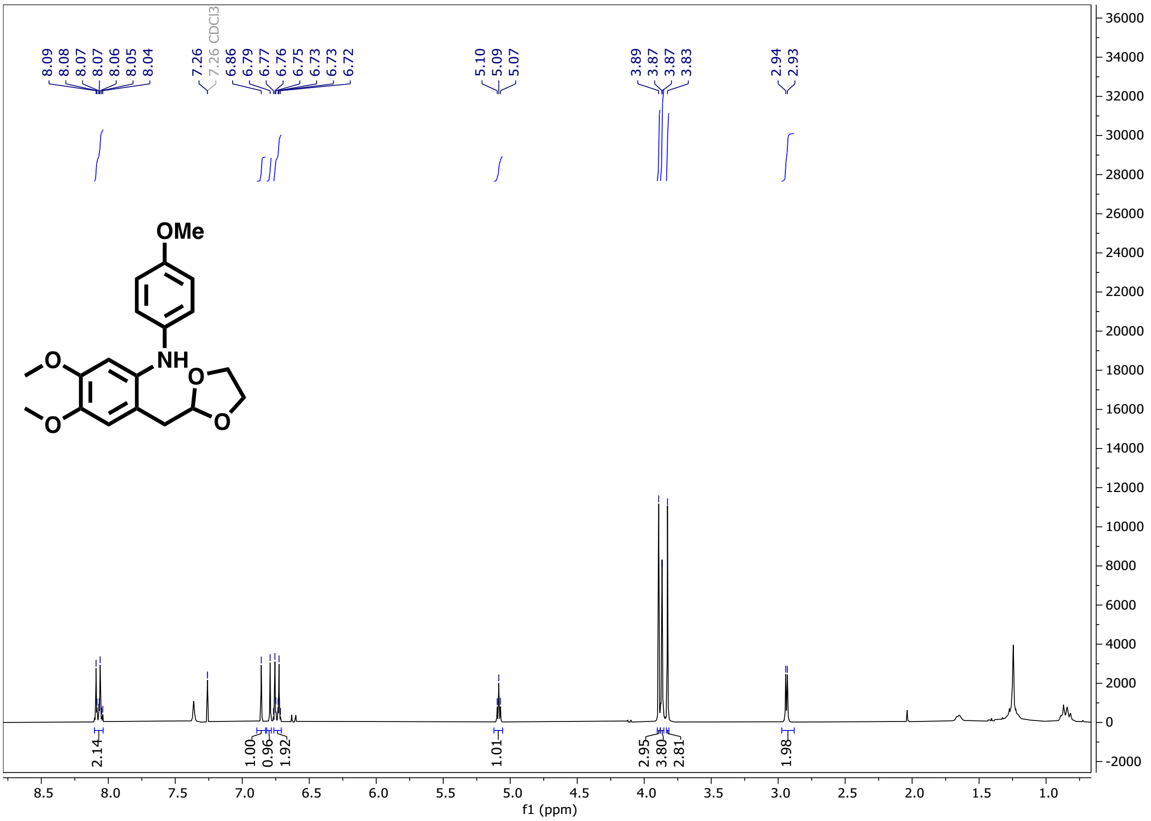

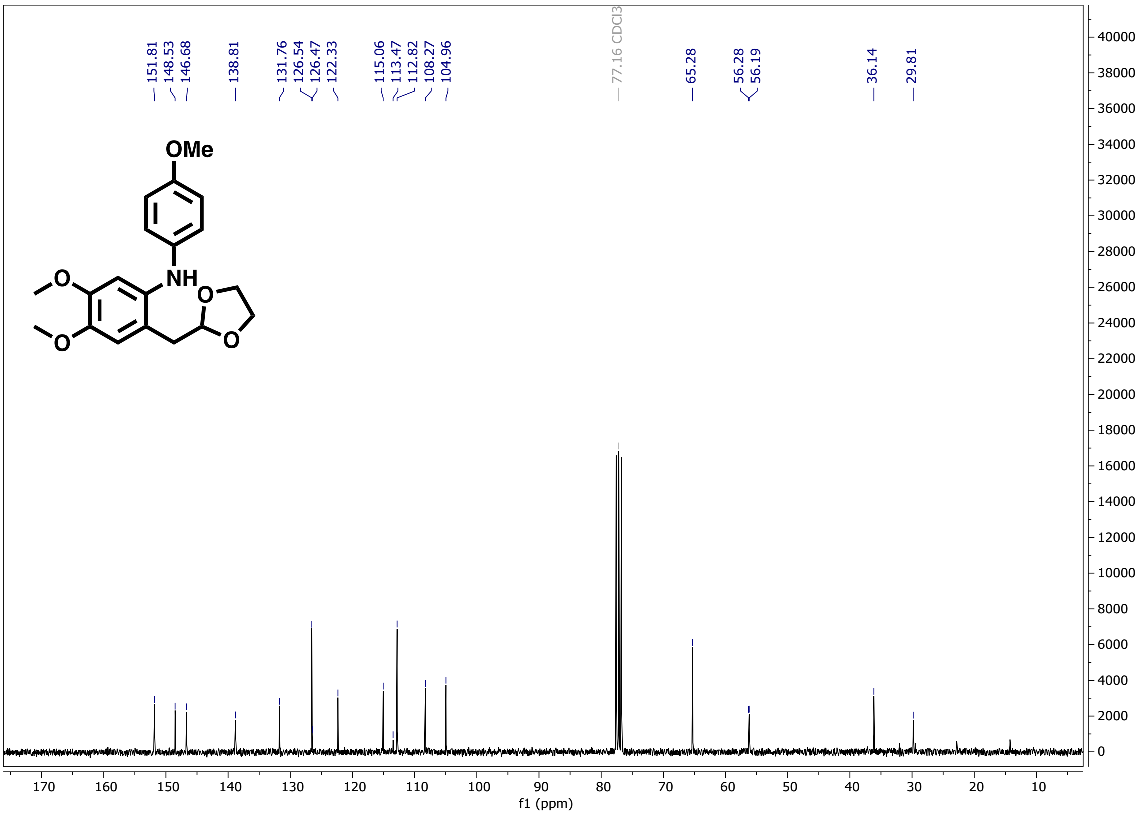

Supplementary Figure 7. ^1H and ^{13}C spectra of compound **C2-Gc3**

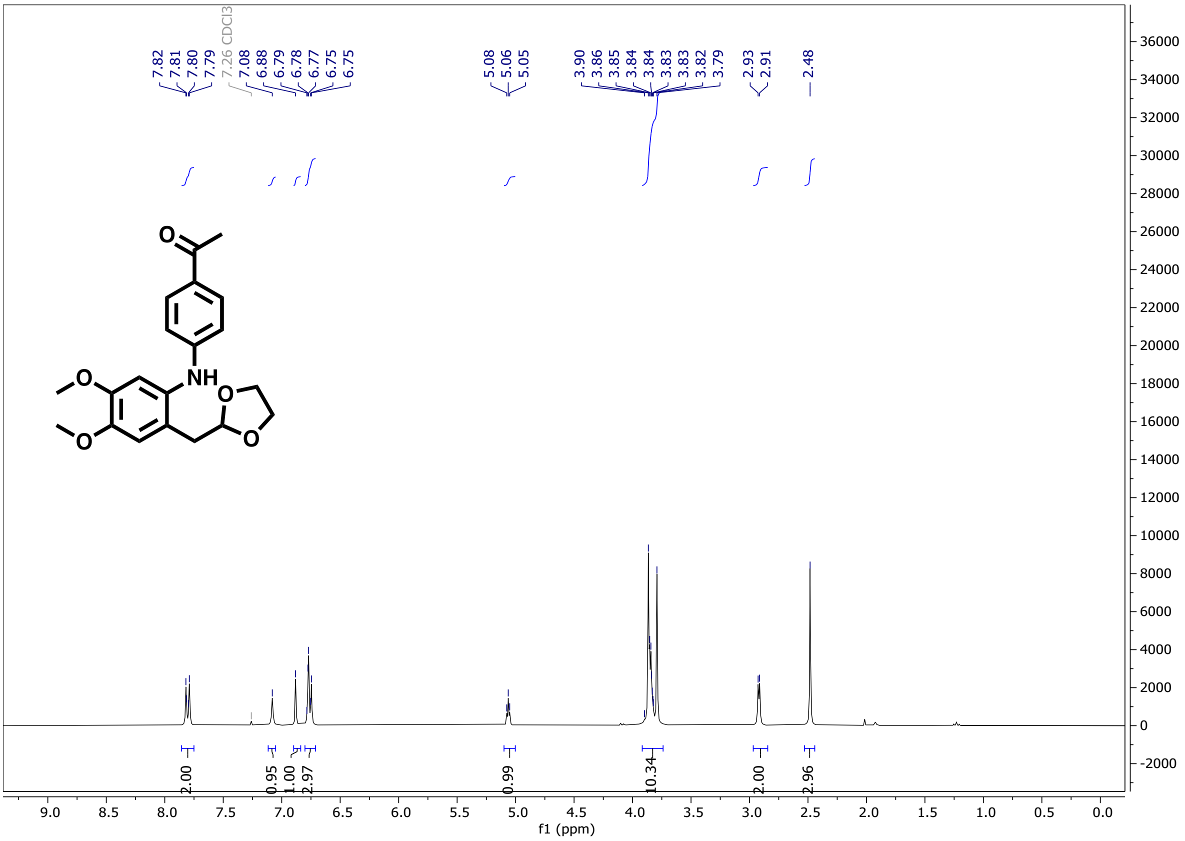
7.82
7.81
7.80
7.79
7.26 CDCl3
7.08
6.88
6.79
6.78
6.77
6.75
6.75
5.08
5.06
5.05
3.90
3.86
3.85
3.84
3.84
3.83
3.83
3.82
3.79
2.93
2.91
2.48
2.00
0.95
1.00
2.97
0.99
10.34
2.00
2.96
f1 (ppm)
NH
O
O
O
O
O

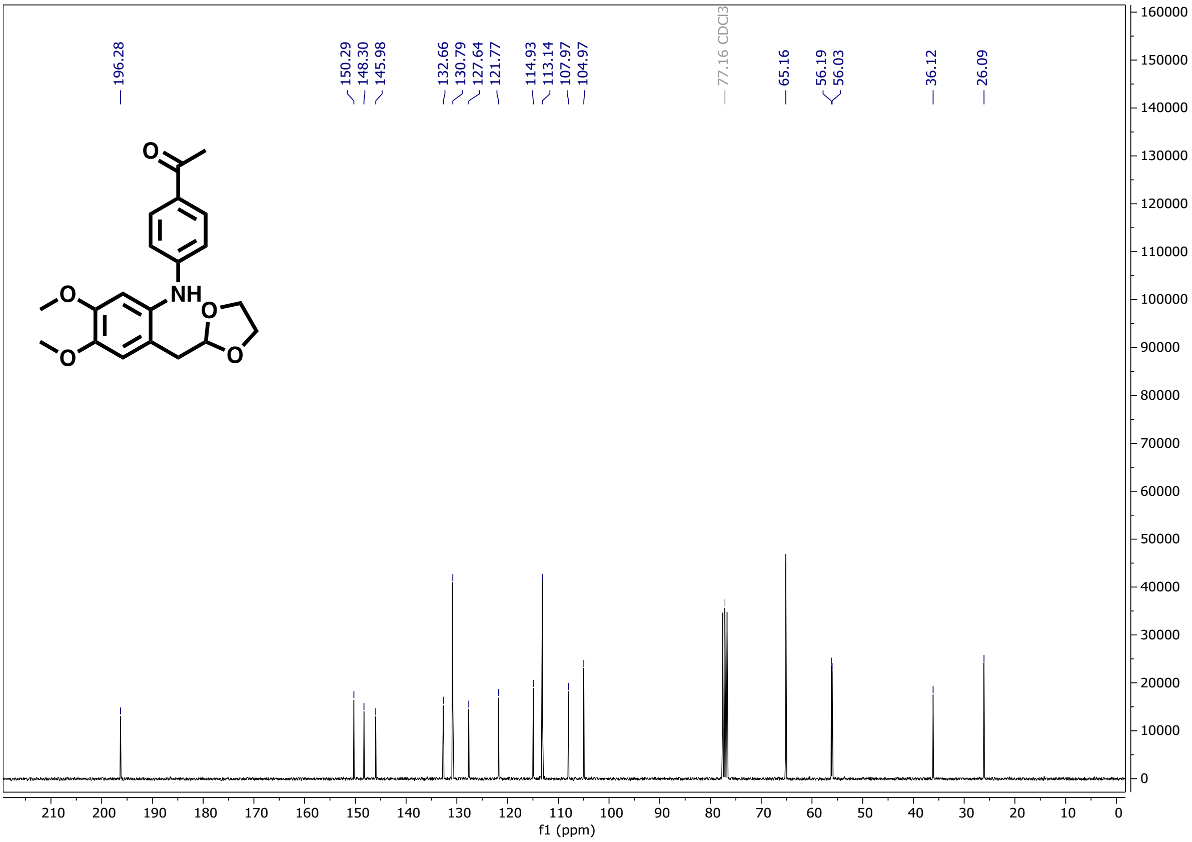
196.28
150.29
148.30
145.98
132.66
130.79
127.64
121.77
114.93
113.14
107.97
104.97
77.16 CDCl3
65.16
56.19
56.03
36.12
26.09
f1 (ppm)
NH
O
O
O
O
O

Supplementary Figure 8. ^{1}H and ^{13}C spectra of compound **C2-Gc4**

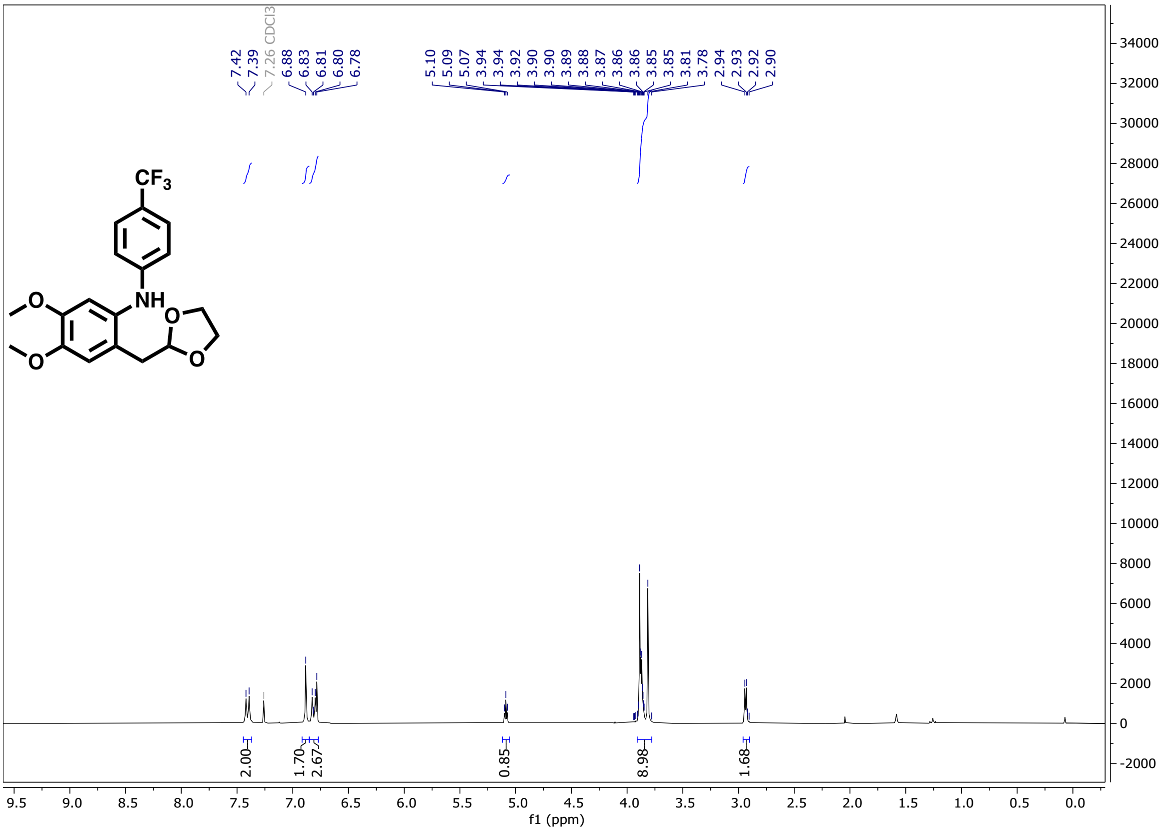

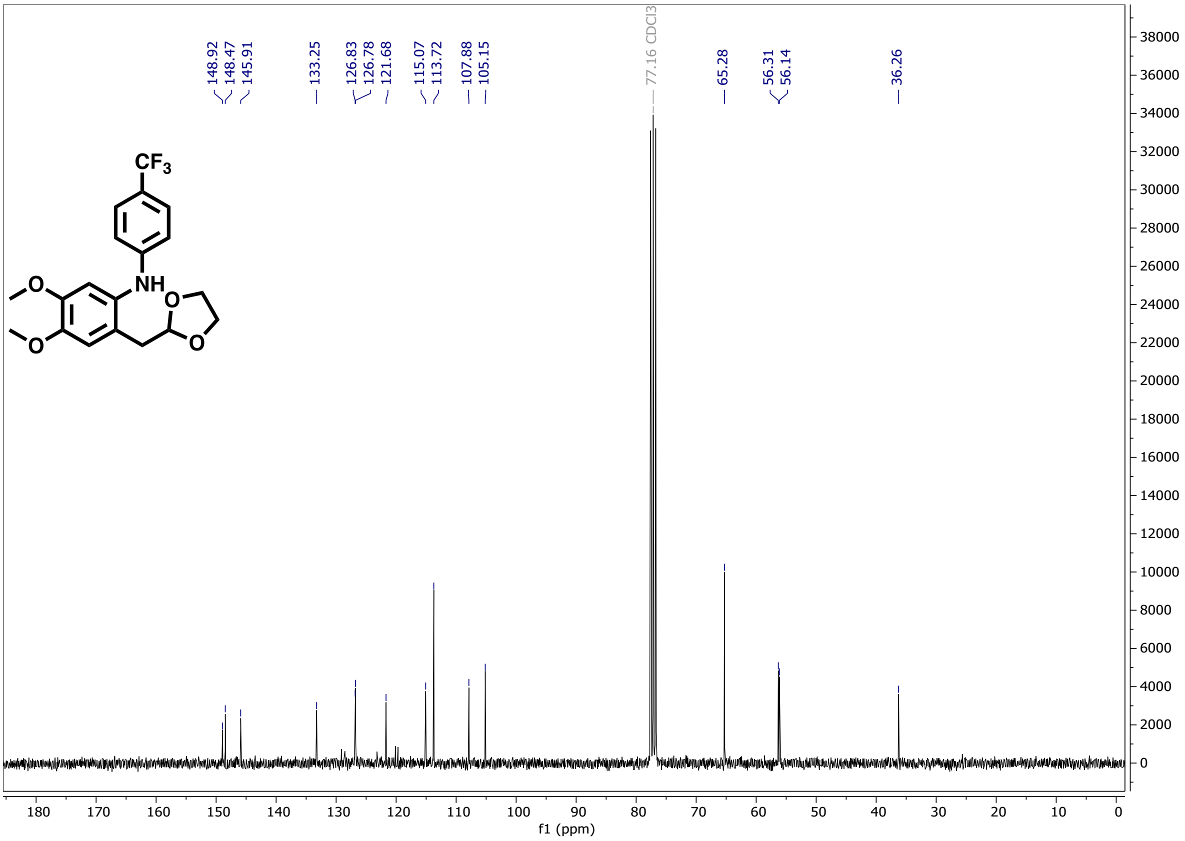

Supplementary Figure 9. ^{1}H and ^{13}C spectra of compound **C2-Gc5**

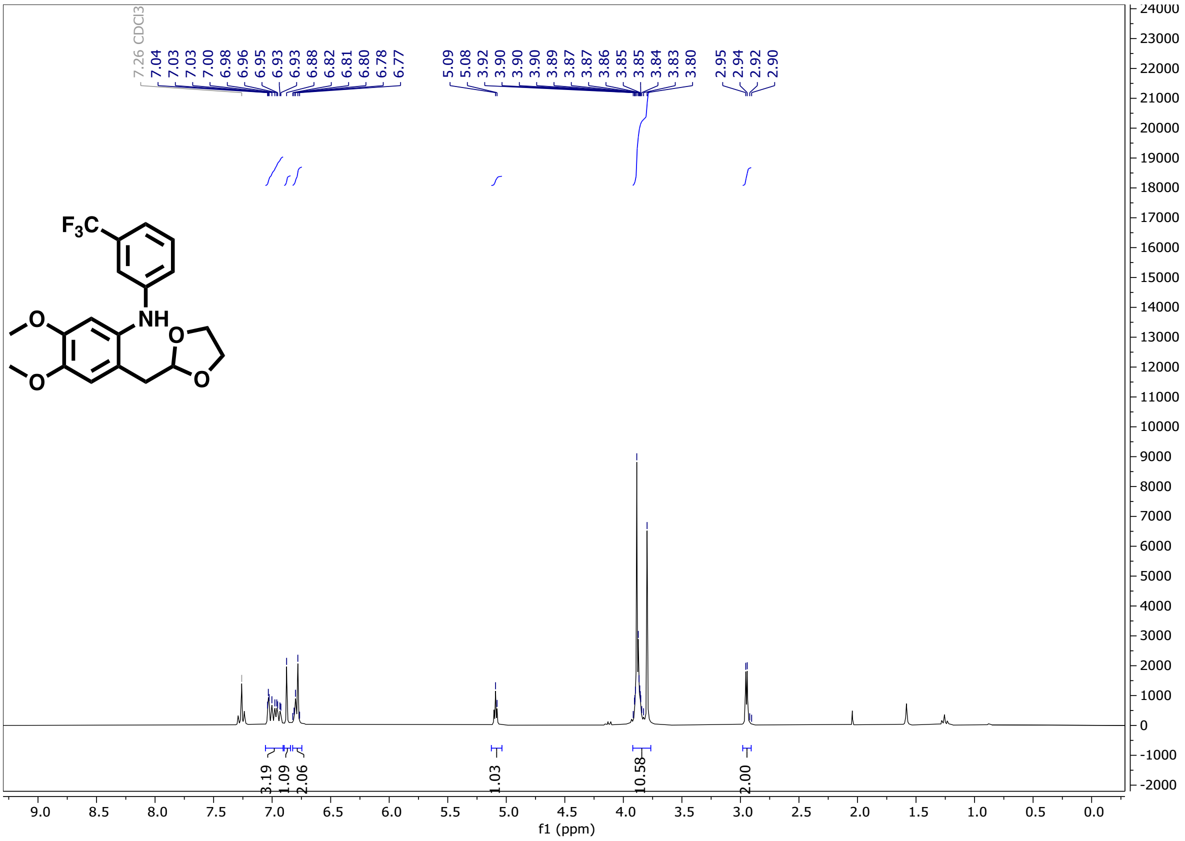

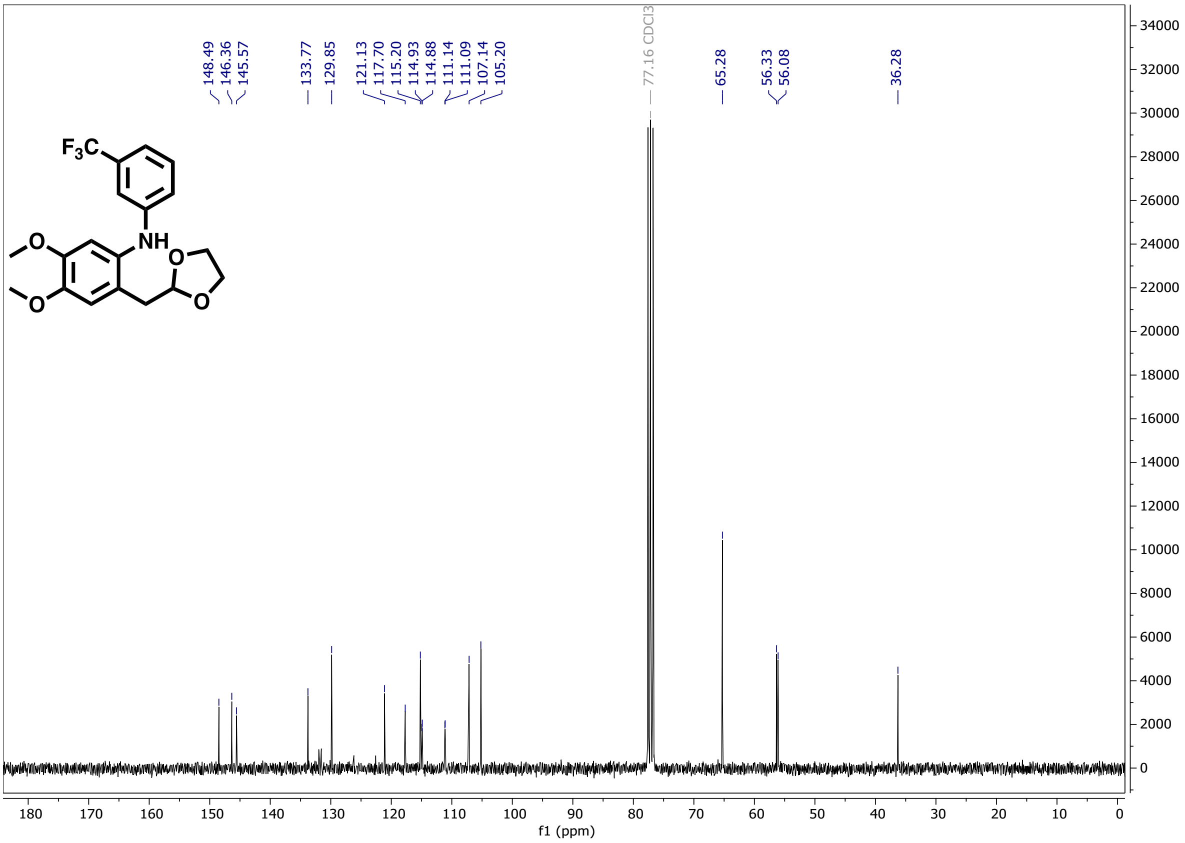

Supplementary Figure 10. ^{1}H and ^{13}C spectra of compound **C2-Gc6**

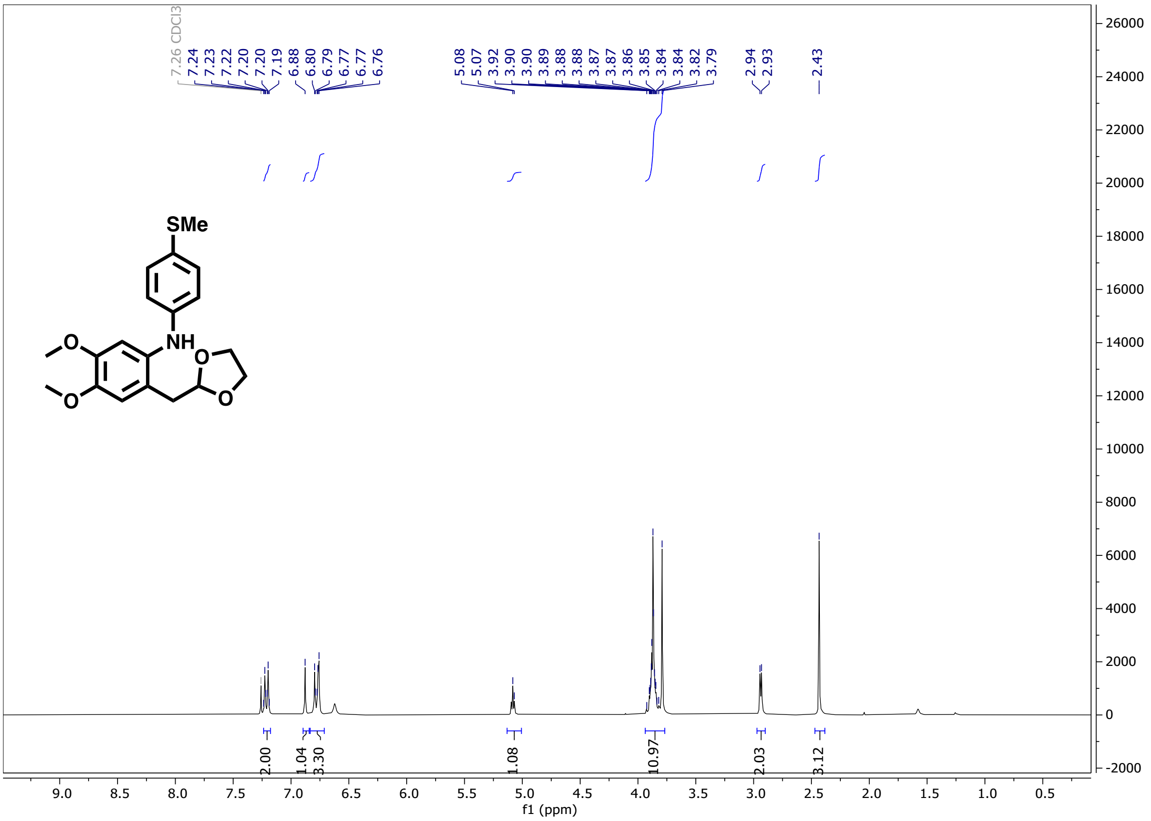

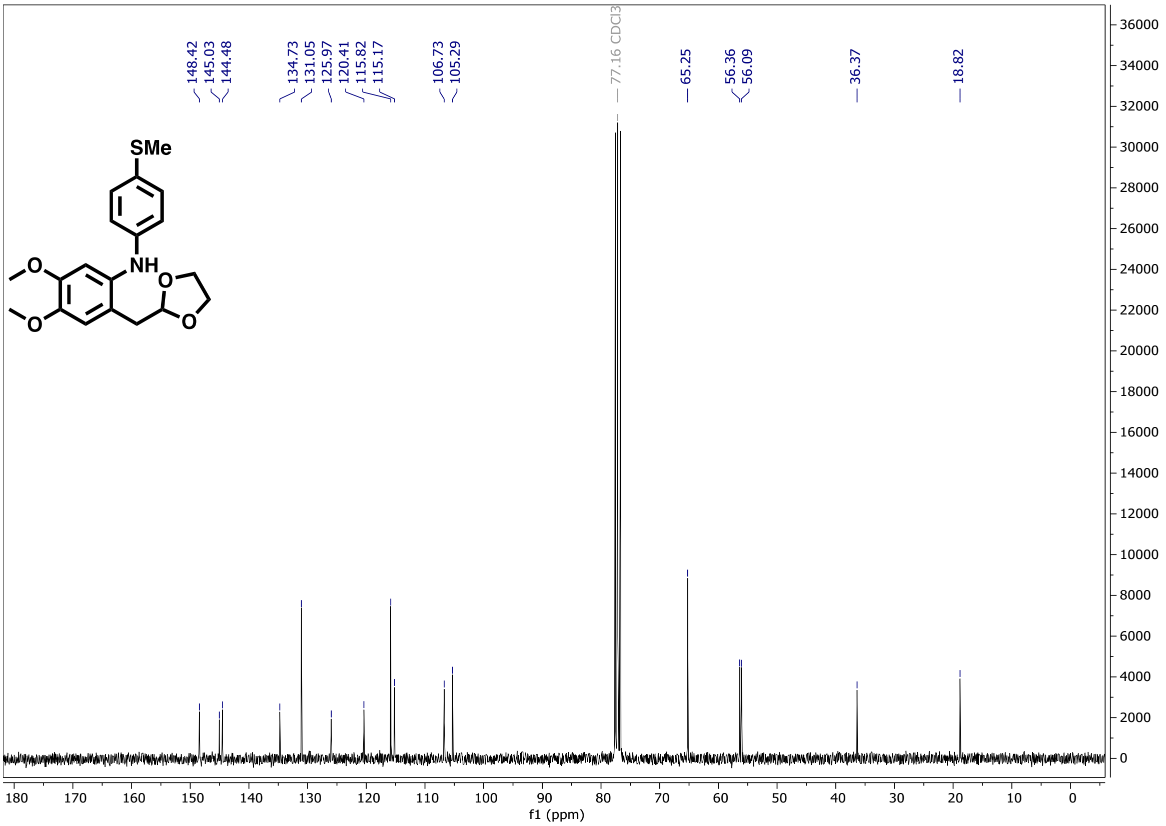

Supplementary Figure 11. ^{1}H and ^{13}C spectra of compound **C2-Gc7**

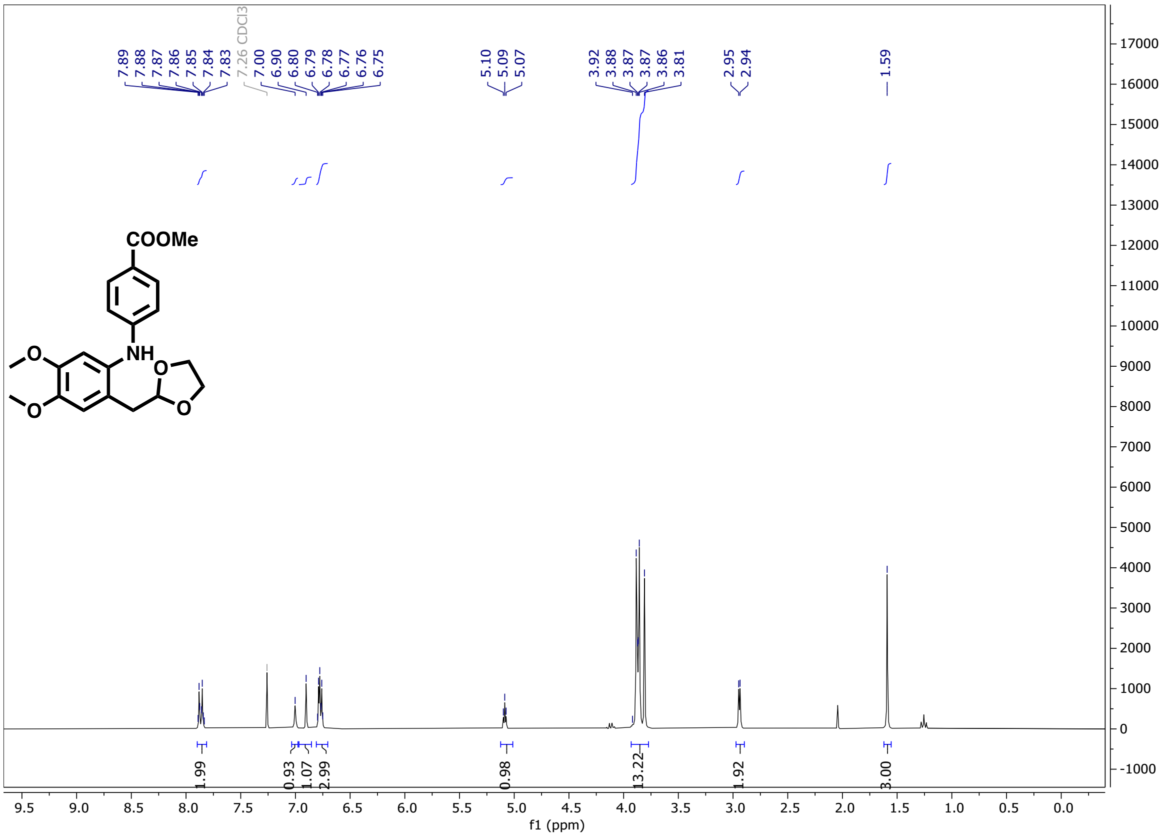

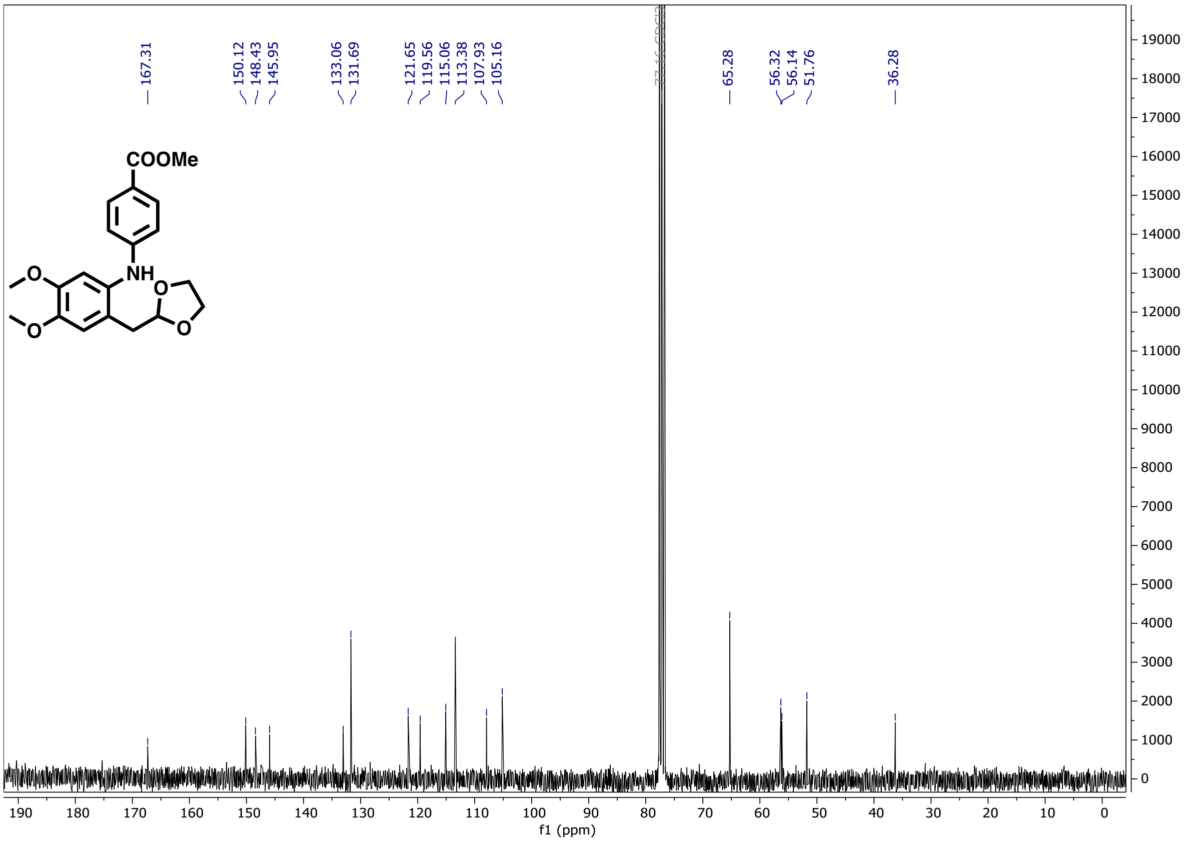

Supplementary Figure 12. ^{1}H and ^{13}C spectra of compound **C2-Gc8**

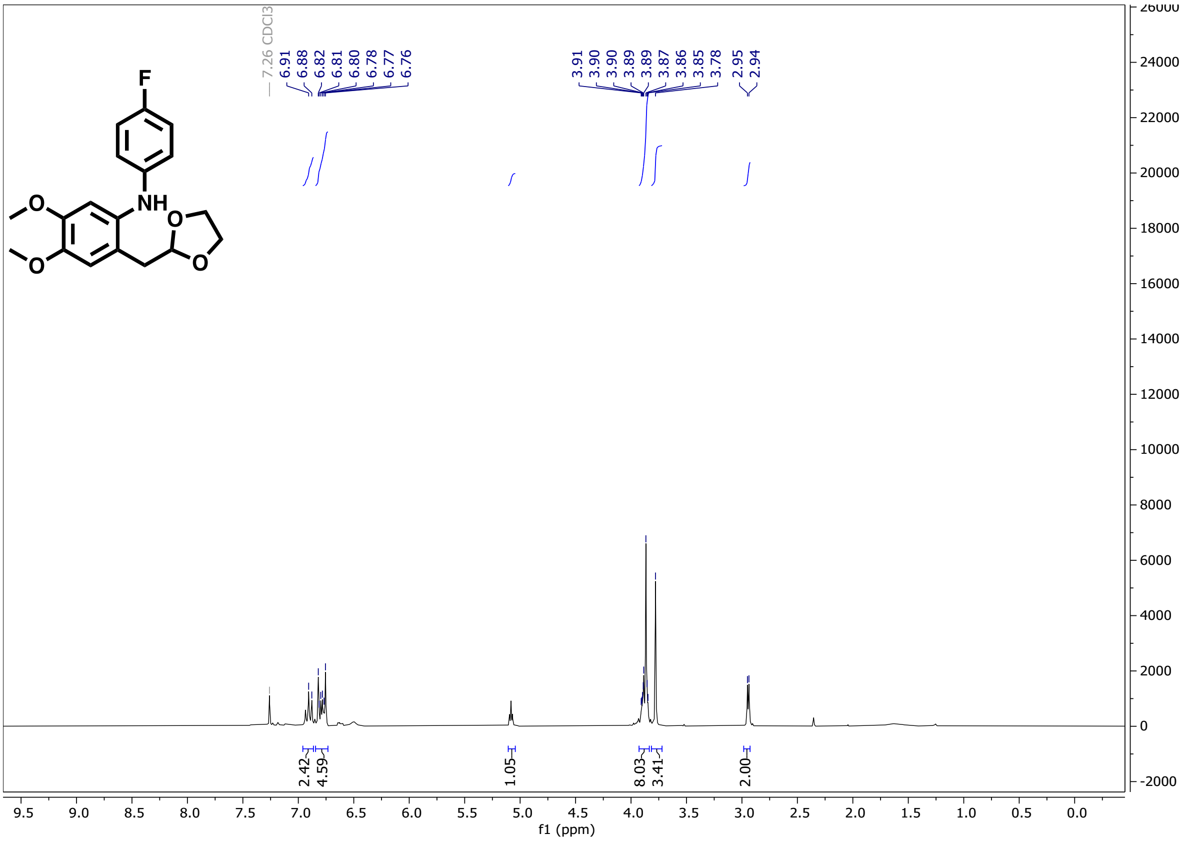

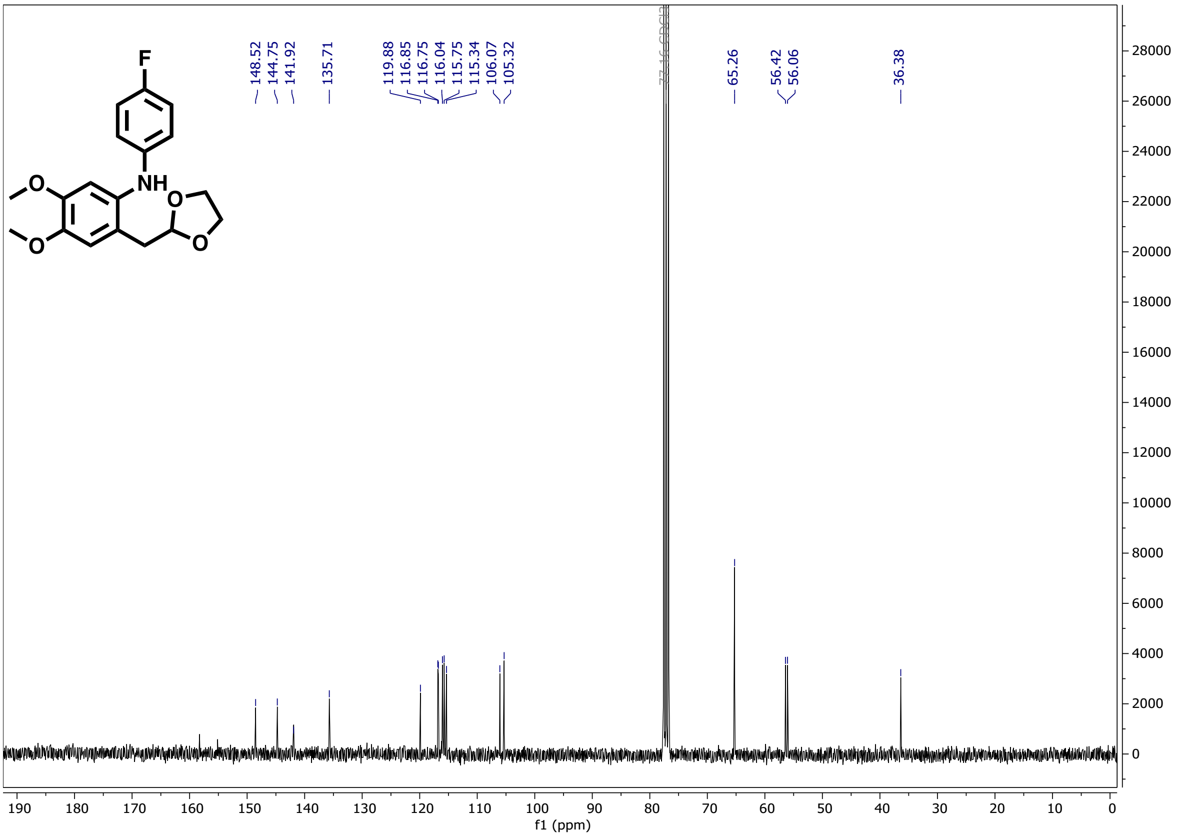

Supplementary Figure 13. ^{1}H and ^{13}C spectra of compound **C2-Gc9**

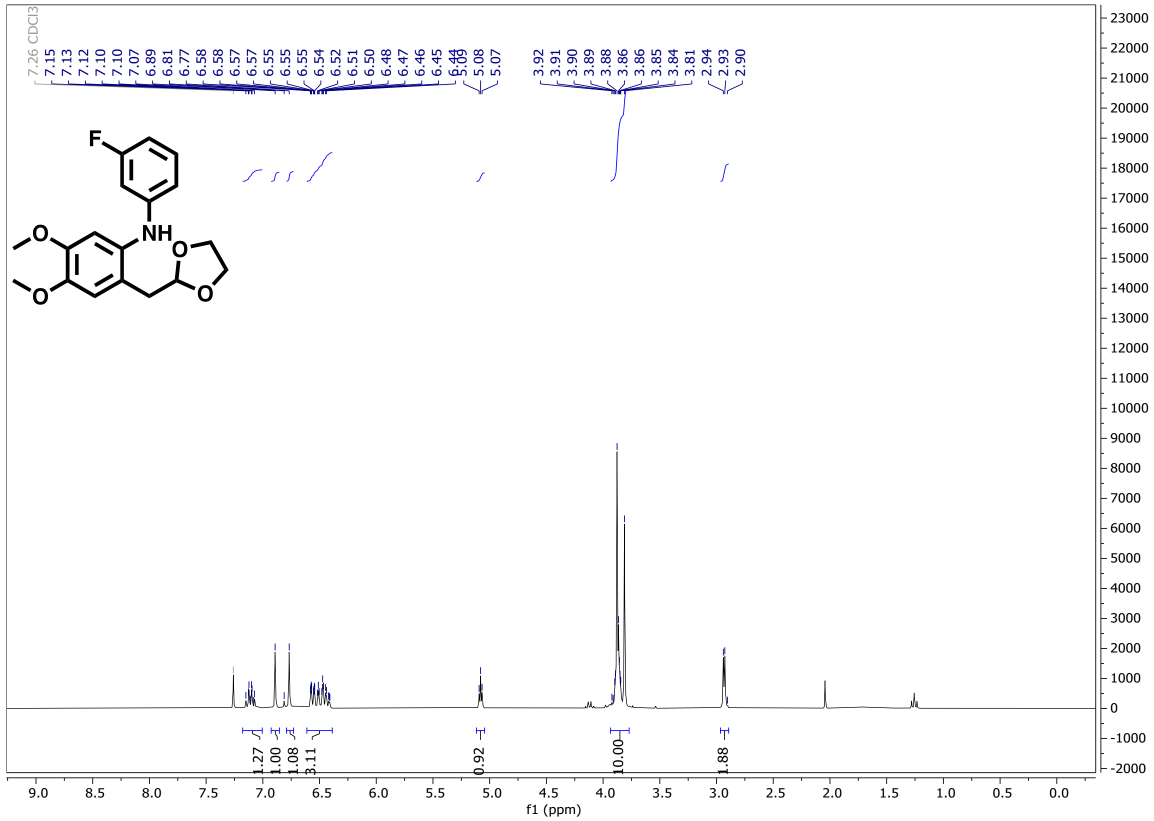

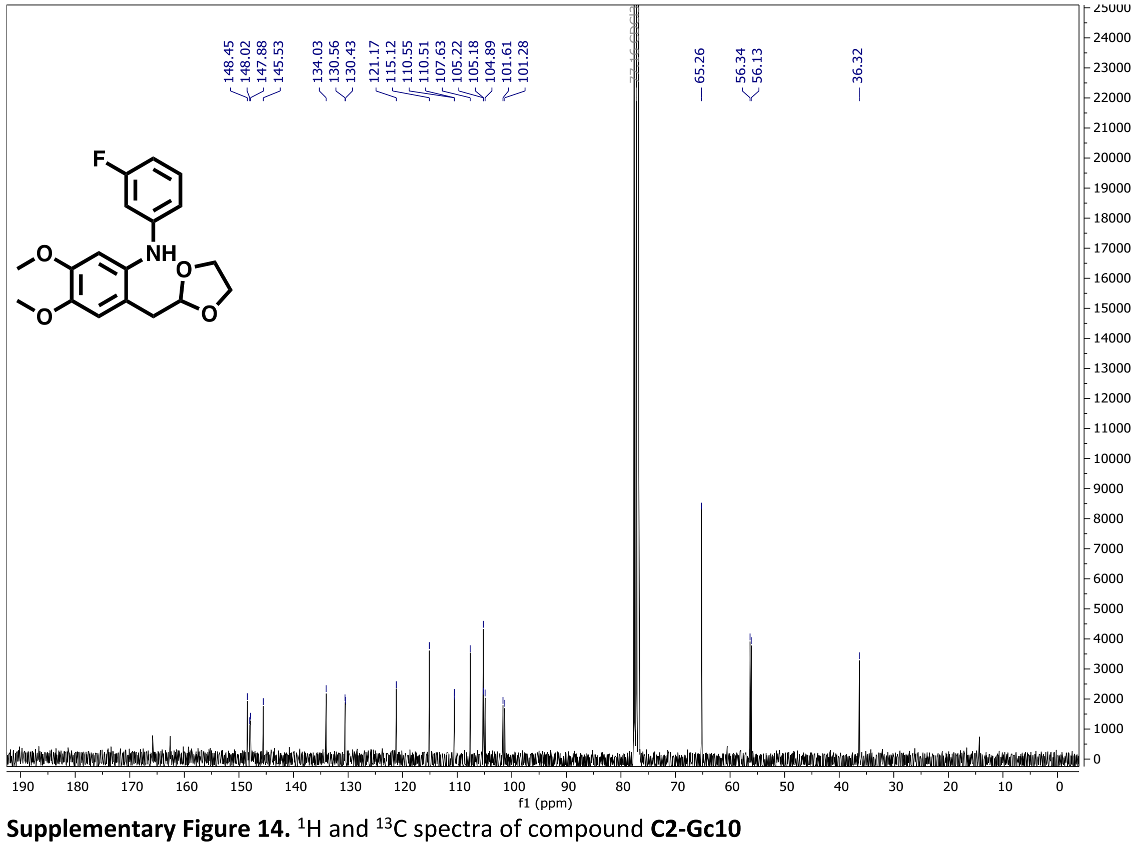

Supplementary Figure 14. ^{1}H and ^{13}C spectra of compound **C2-Gc10**

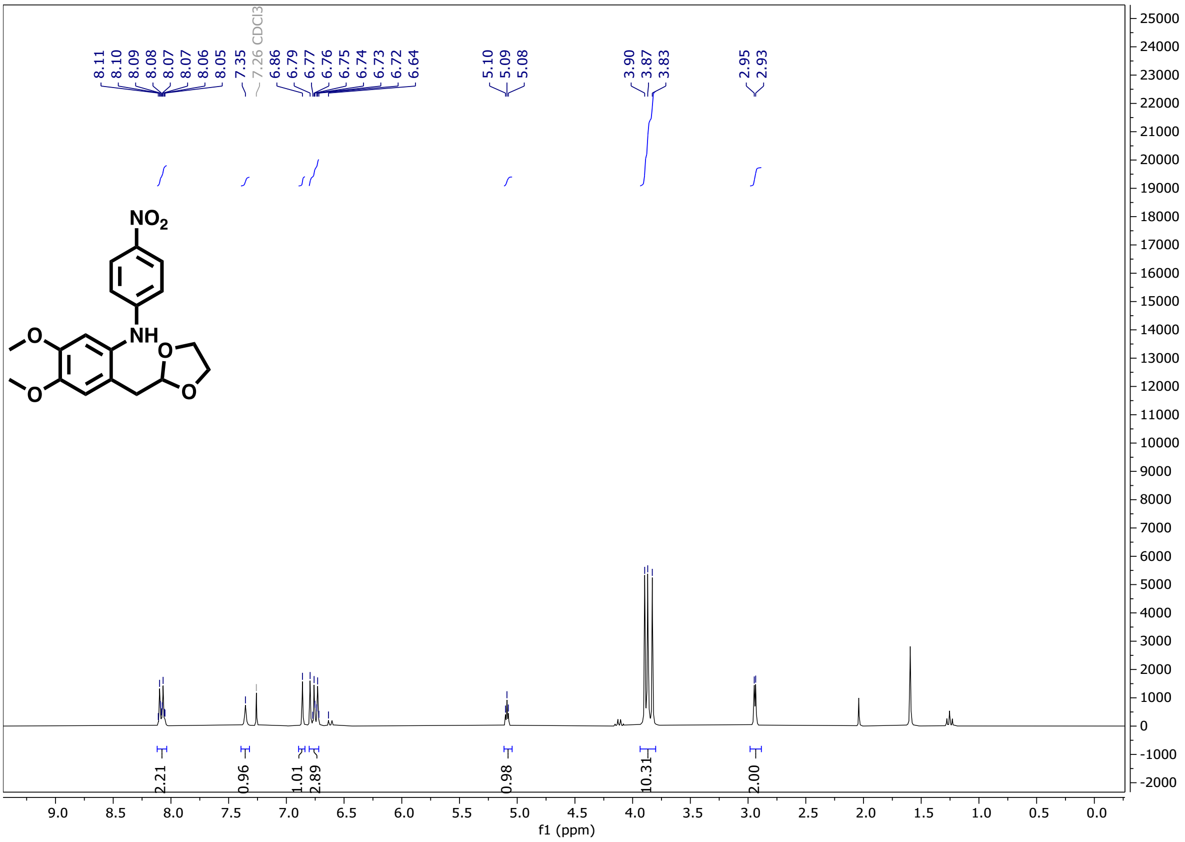

8.11
8.10
8.09
8.08
8.07
8.07
8.06
8.05
7.35
7.26 CDCl3
6.86
6.79
6.77
6.76
6.75
6.74
6.73
6.72
6.64
5.10
5.09
5.08
3.90
3.87
3.83
2.95
2.93
NO2
NH
O
2.21
0.96
1.01
2.89
0.98
10.31
2.00
f1 (ppm)

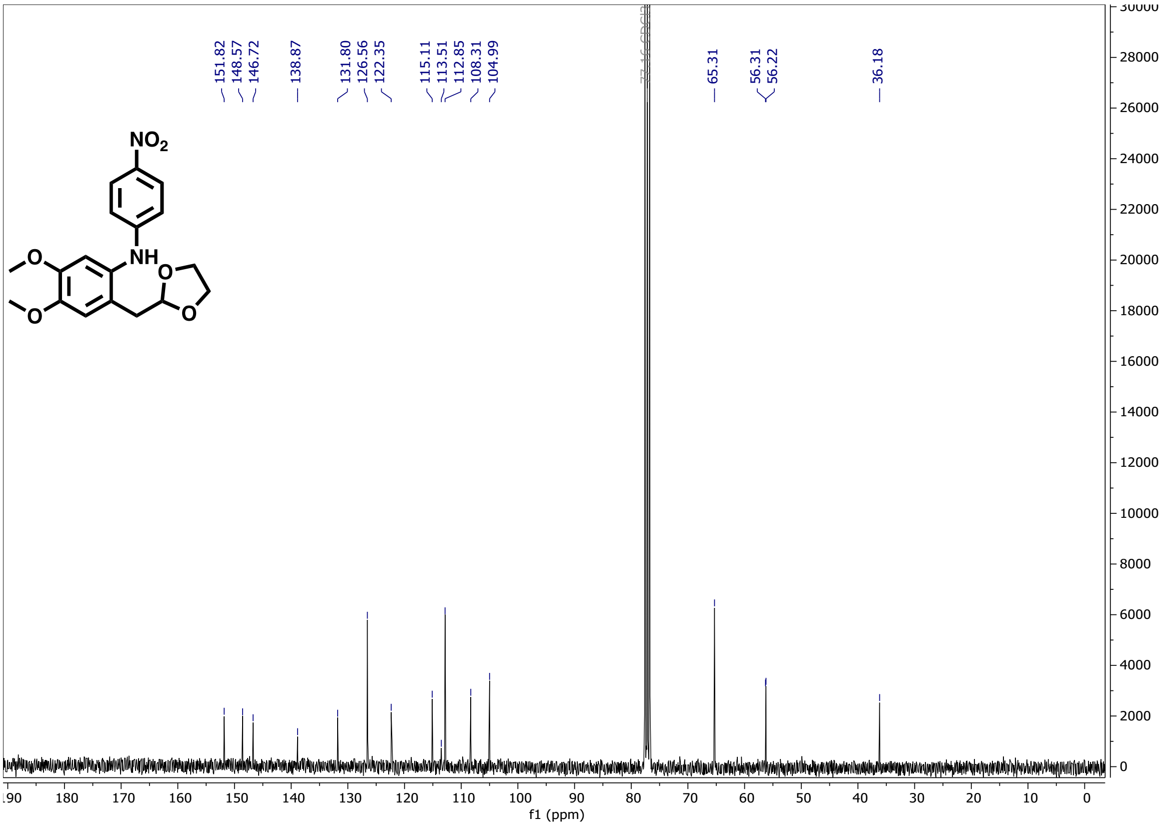

Supplementary Figure 15. ^{1}H and ^{13}C spectra of compound **C2-Gc11**

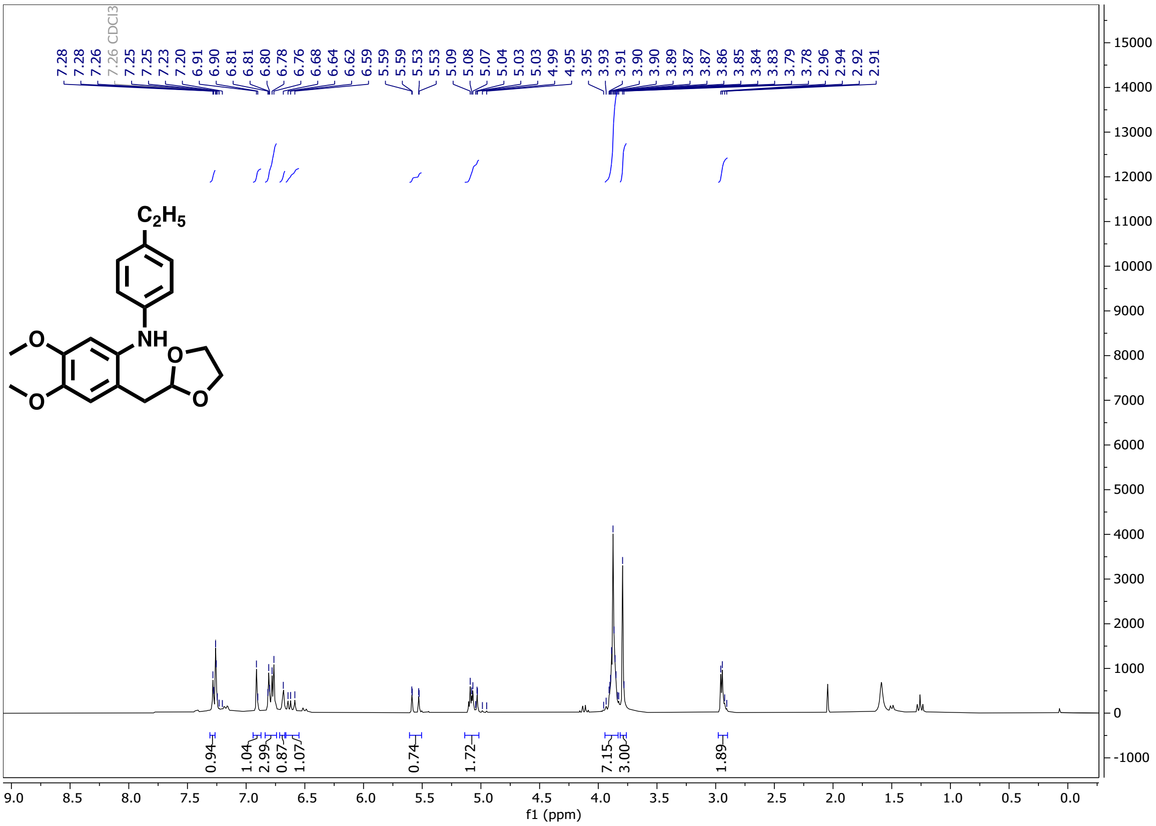

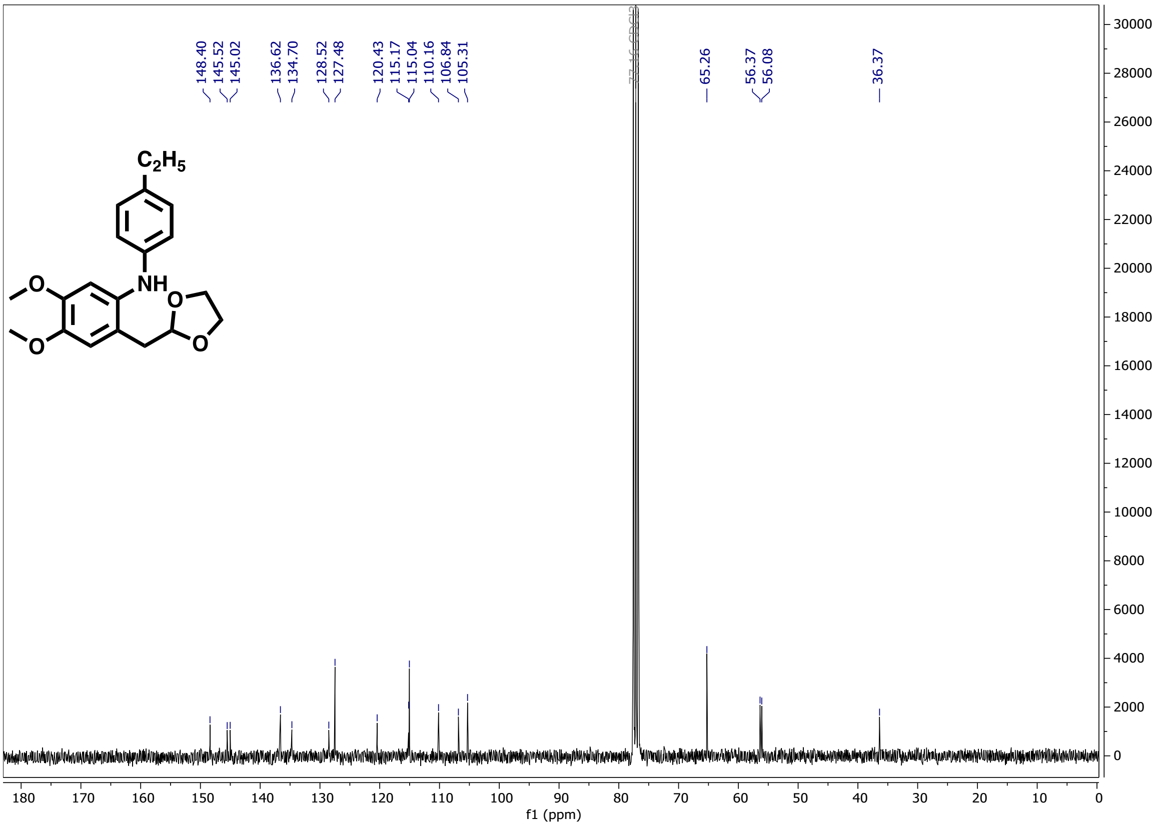

Supplementary Figure 16. ^{1}H and ^{13}C spectra of compound **C2-Gc12**

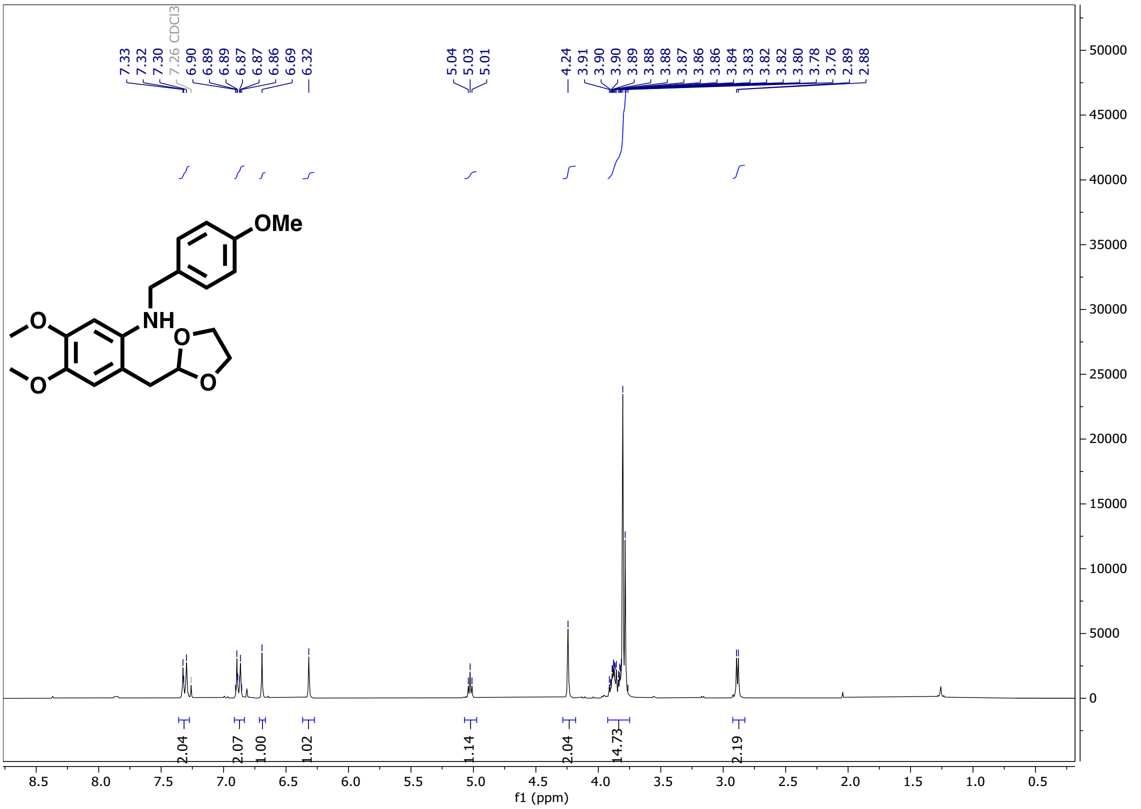

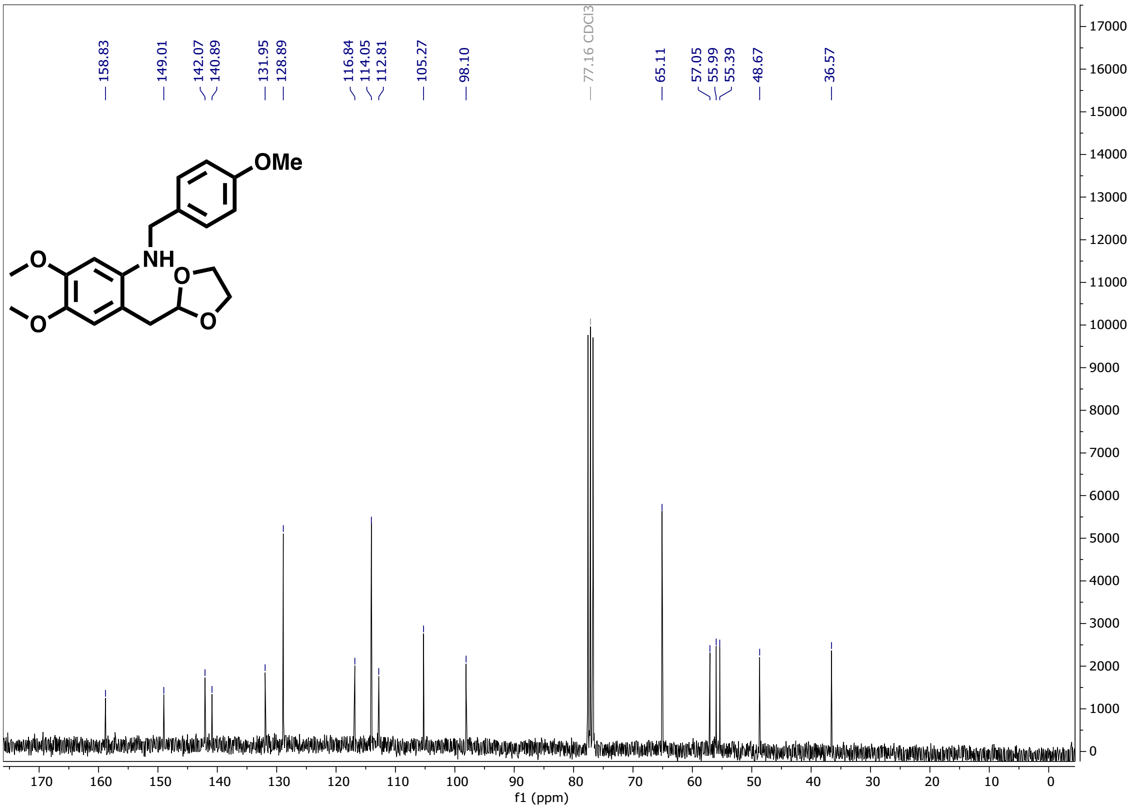

Supplementary Figure 17. ^{1}H and ^{13}C spectra of compound **C2-Gc13**

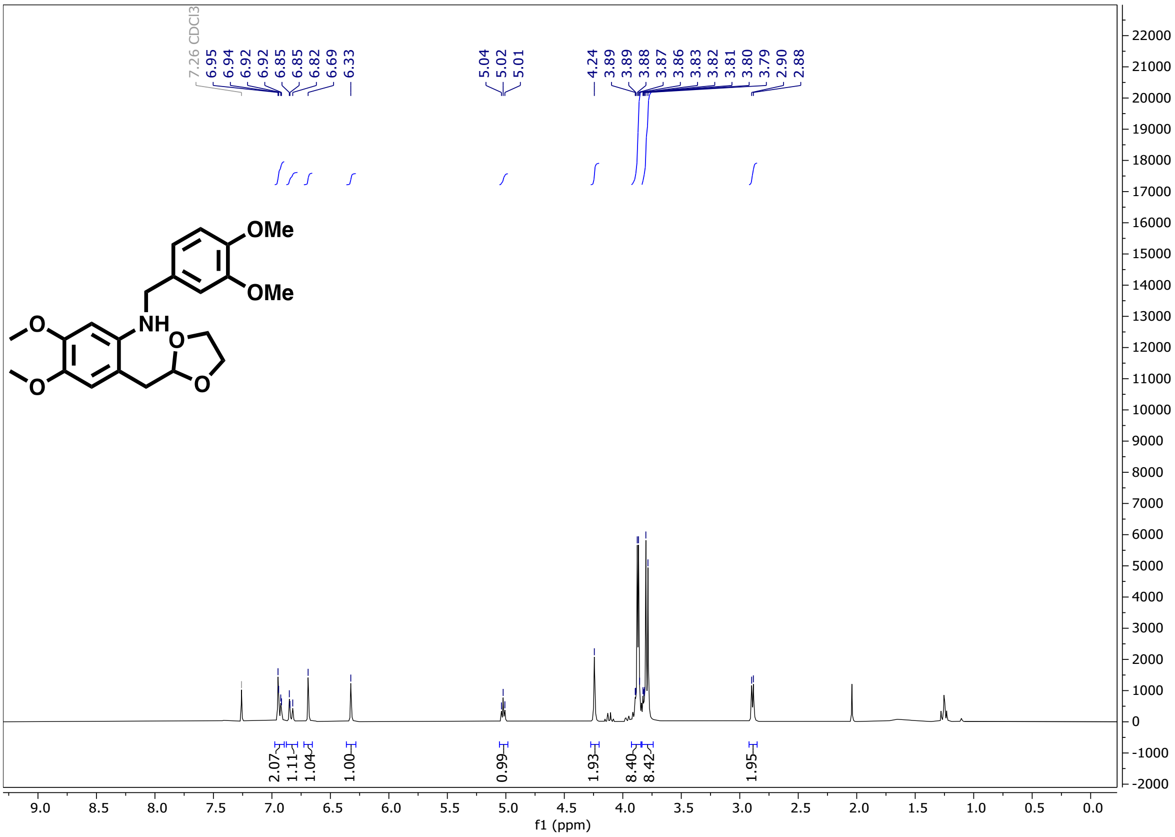

7.26 CDCl3
6.95
6.94
6.92
6.92
6.85
6.85
6.82
6.69
6.33
5.04
5.02
5.01
4.24
3.89
3.89
3.88
3.87
3.86
3.83
3.82
3.81
3.80
3.79
2.90
2.88
OMe
OMe
NH
O
O
O
O
2.07
1.11
1.04
1.00
0.99
1.93
8.40
8.42
1.95
f1 (ppm)

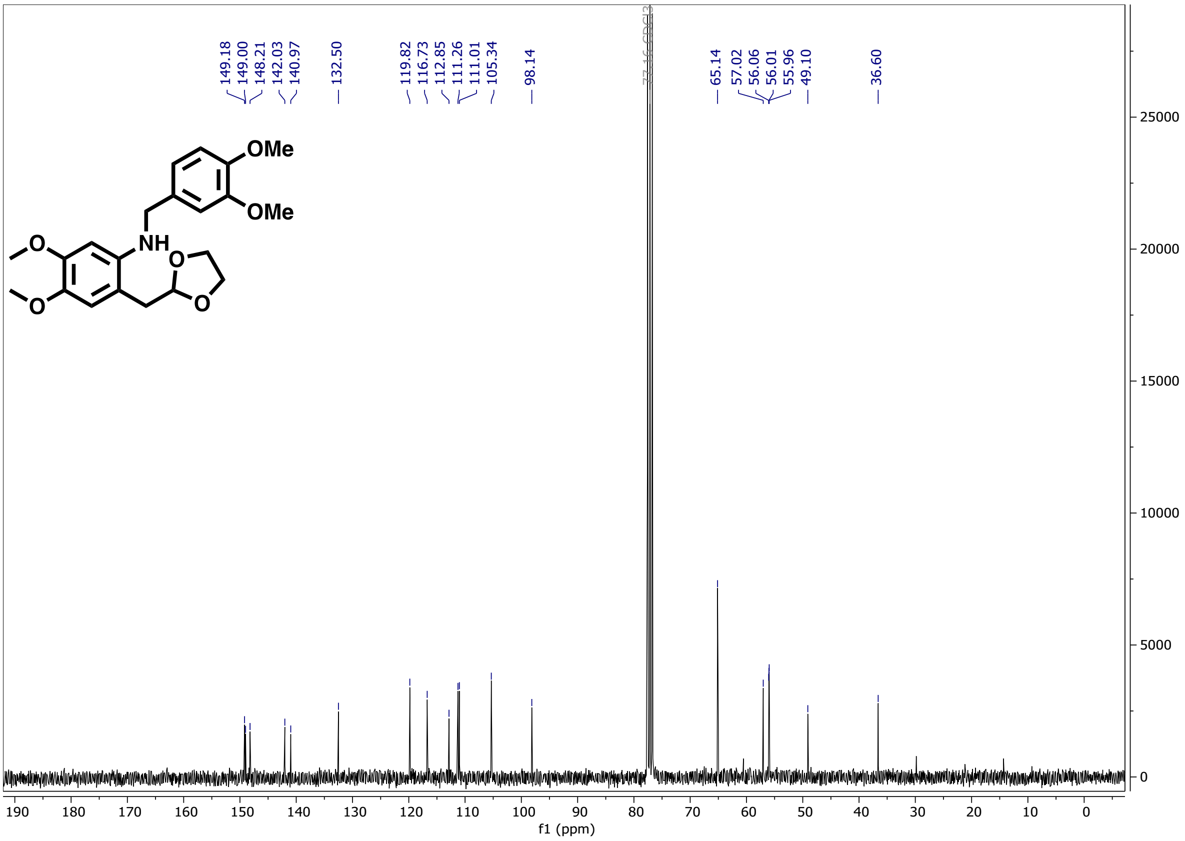

Supplementary Figure 18. ^{1}H and ^{13}C spectra of compound **C2-Gc14**

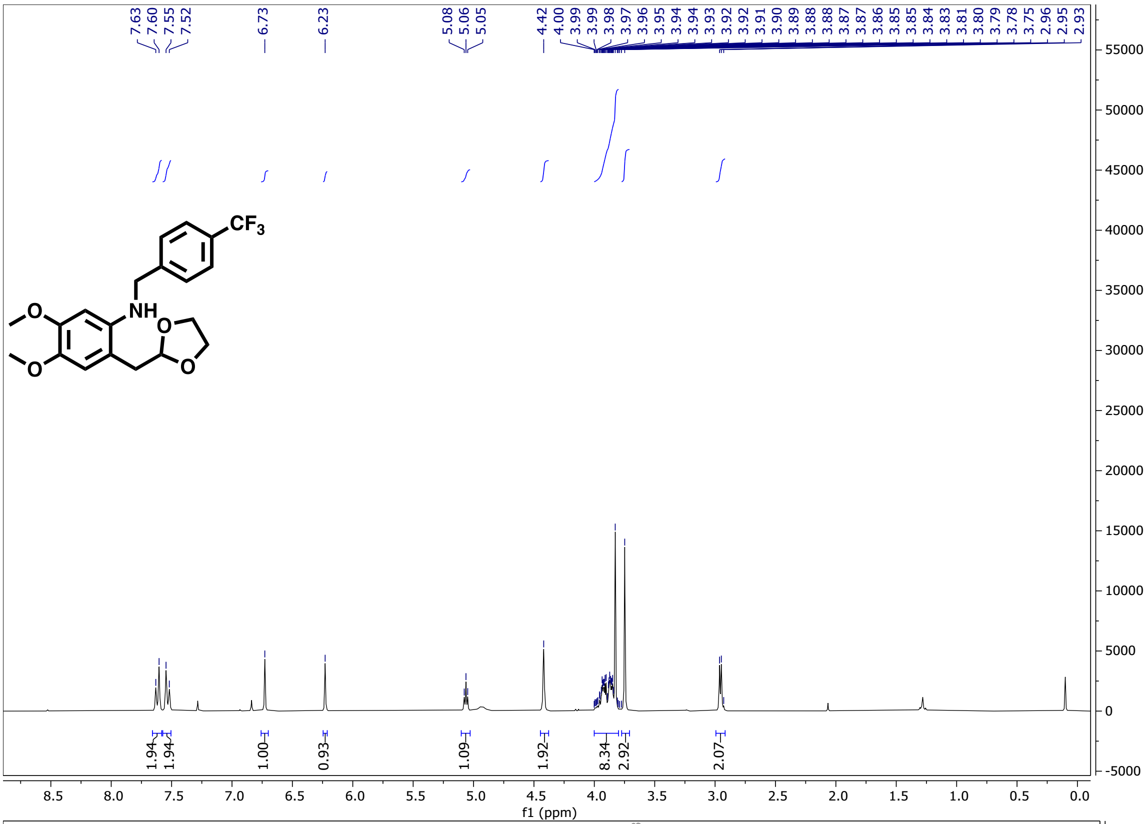

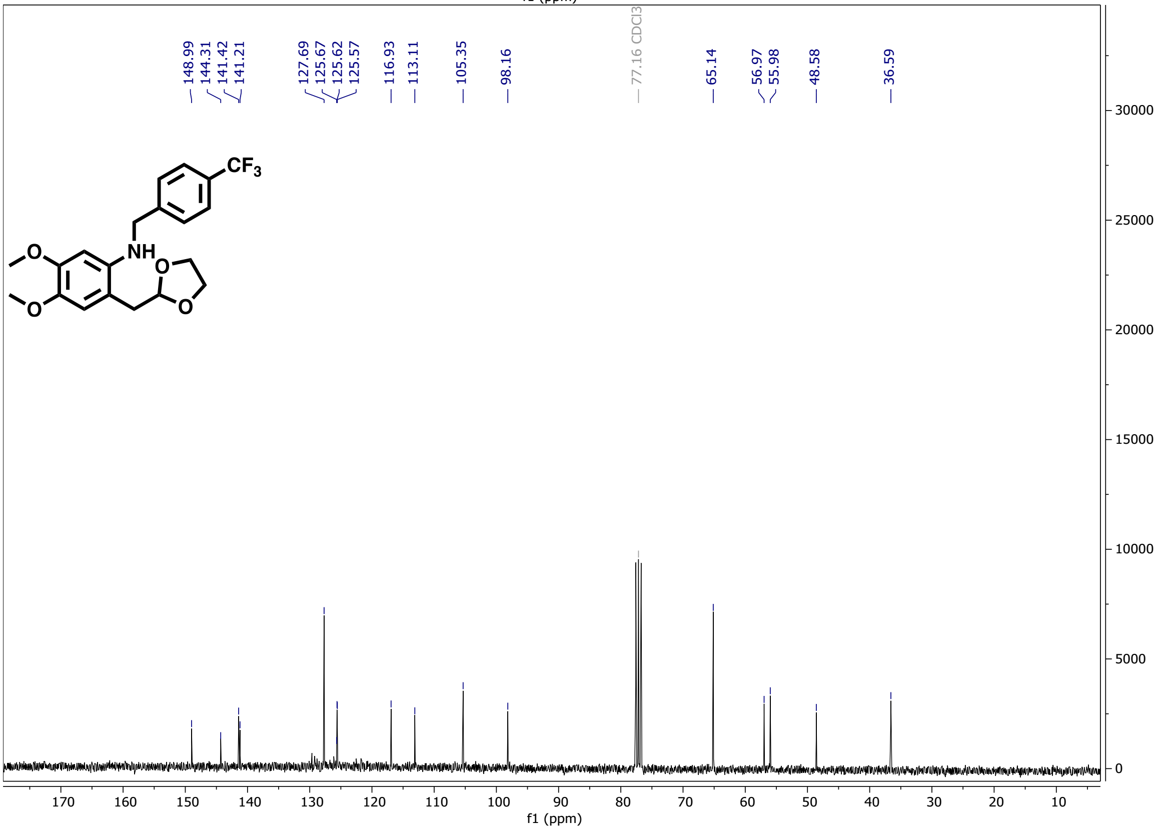

Supplementary Figure 19. ^{1}H and ^{13}C spectra of compound **C2-Gc15**

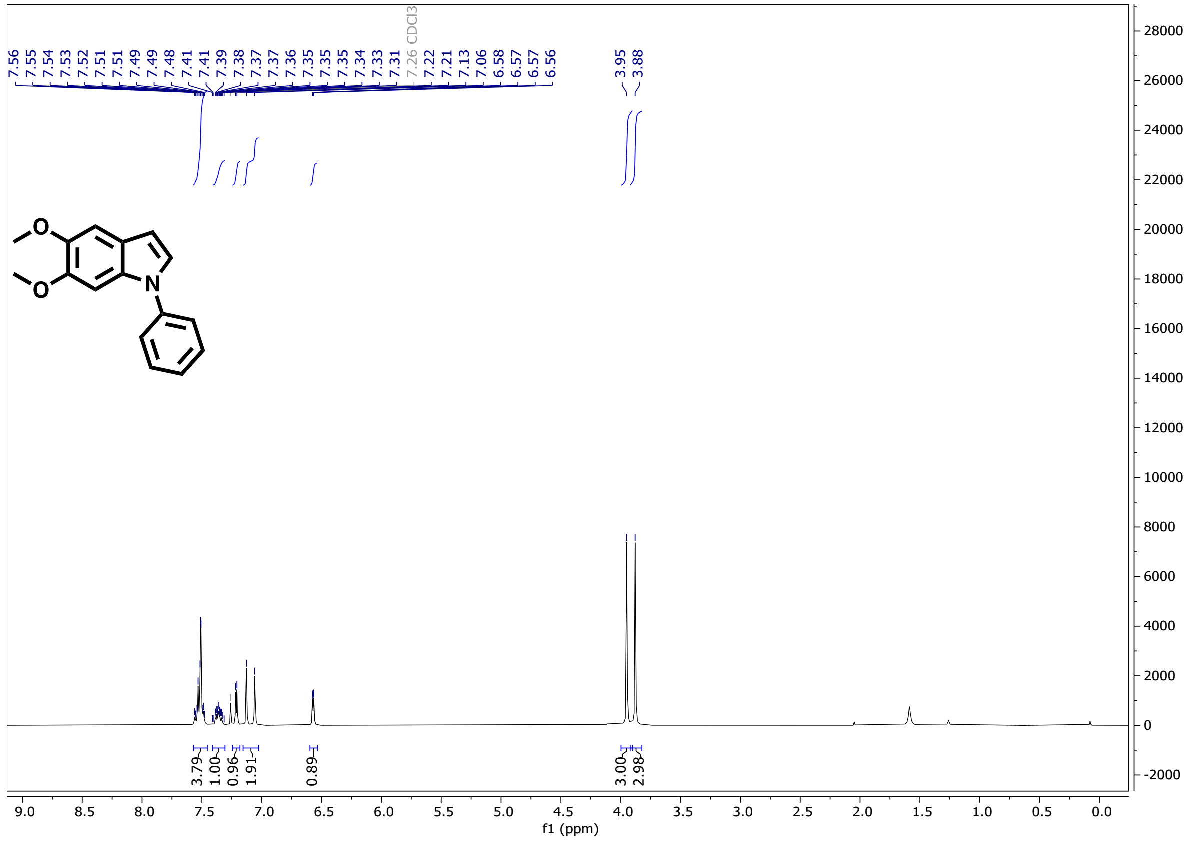

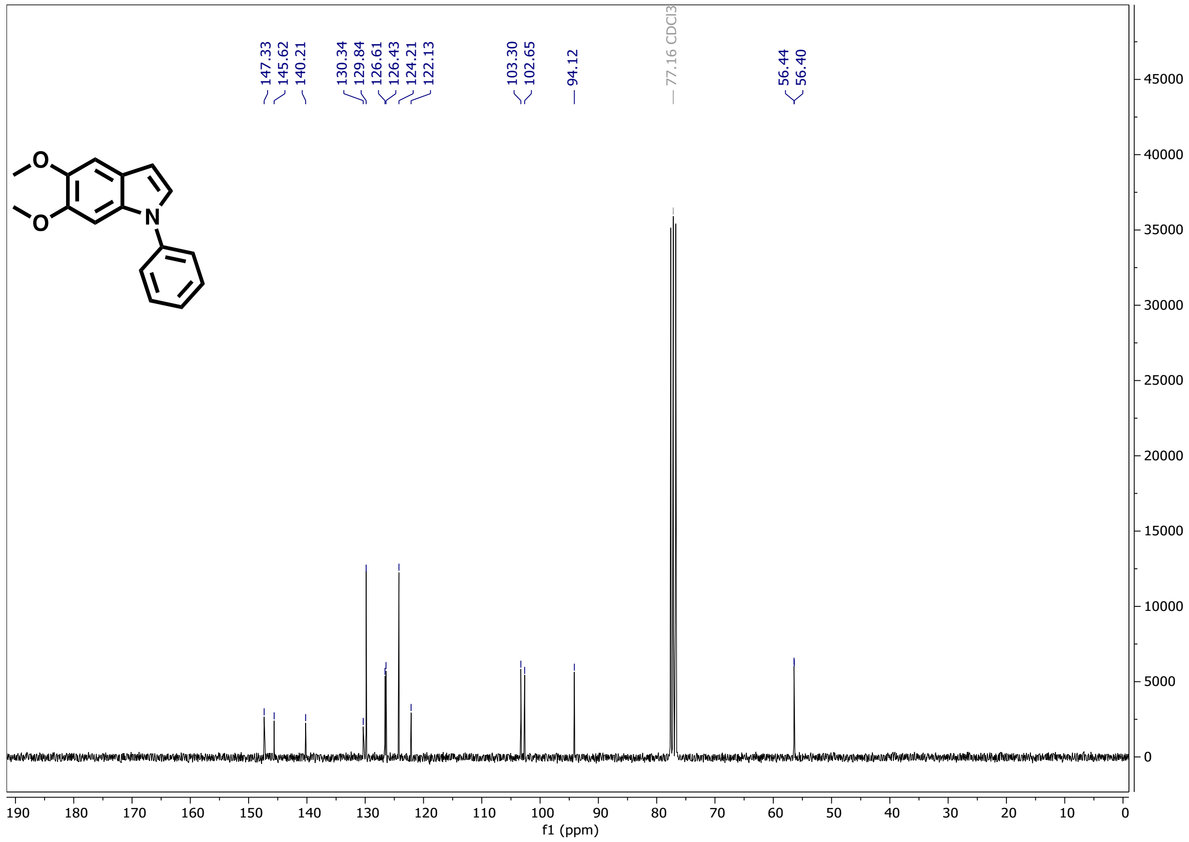

Supplementary Figure 20. ^{1}H and ^{13}C spectra of compound **C2-Gd1**

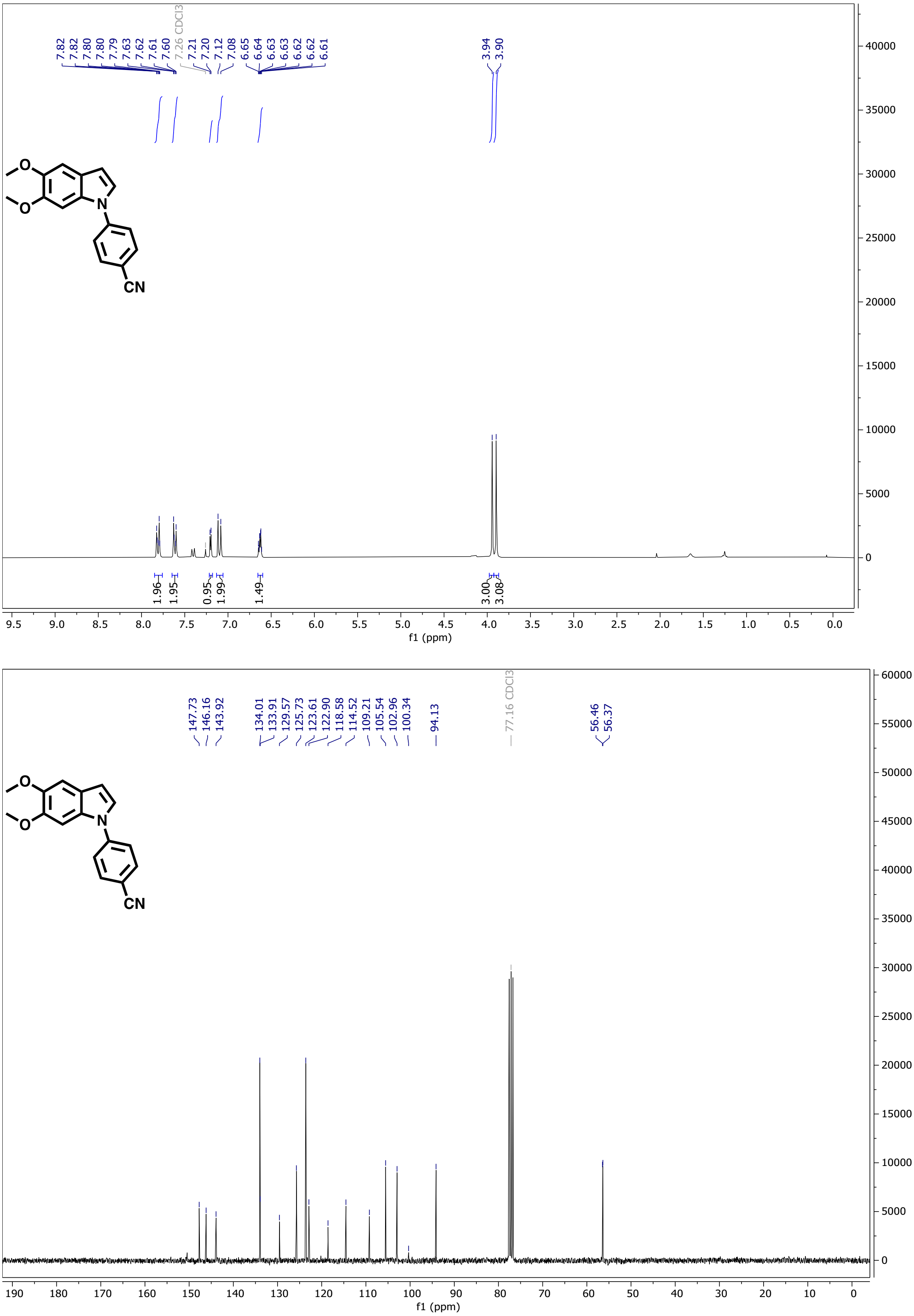

Supplementary Figure 21. ^{1}H and ^{13}C spectra of compound **C2-Gd2**

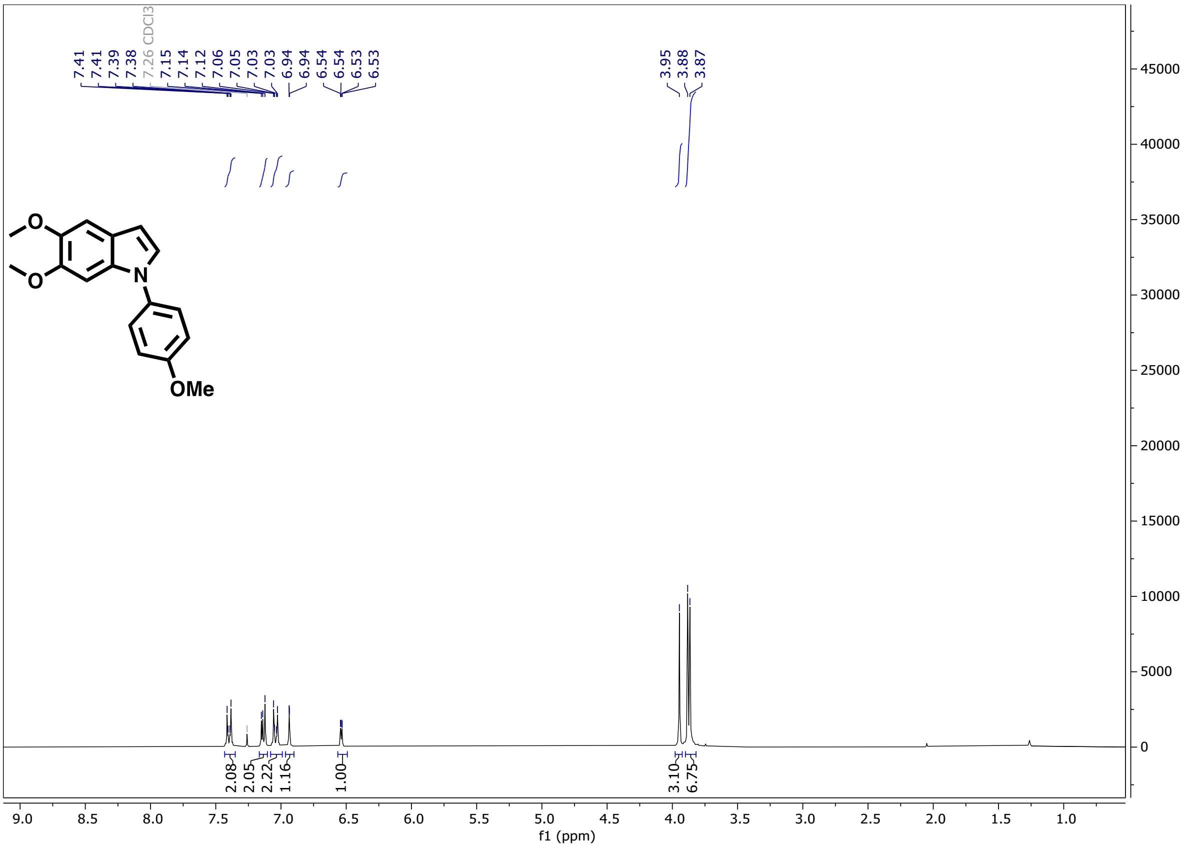

7.41
7.41
7.39
7.38
7.26 CDCl3
7.15
7.14
7.12
7.06
7.05
7.03
7.03
6.94
6.94
6.54
6.54
6.53
6.53
3.95
3.88
3.87
OMe
N
O
O
2.08
2.05
2.22
1.16
1.00
3.10
6.75
f1 (ppm)

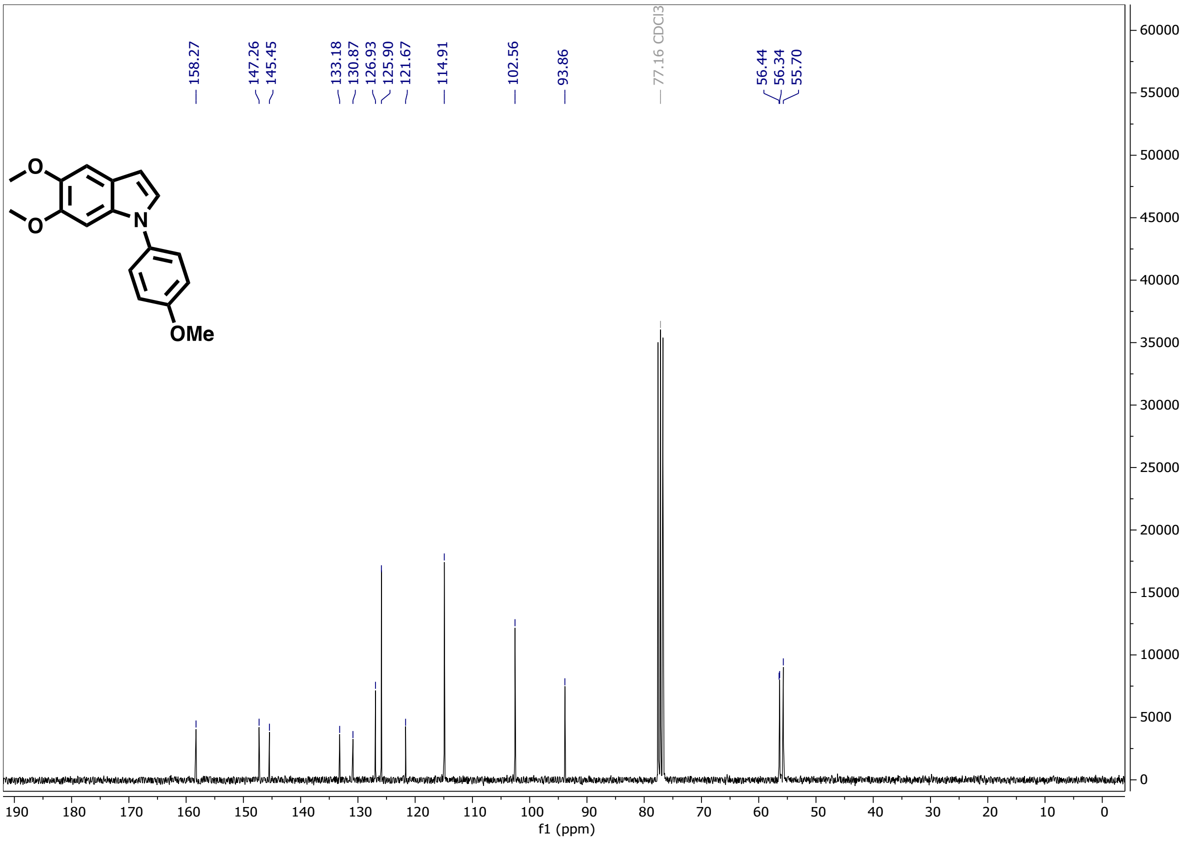

Supplementary Figure 22. ^{1}H and ^{13}C spectra of compound **C2-Gd3**

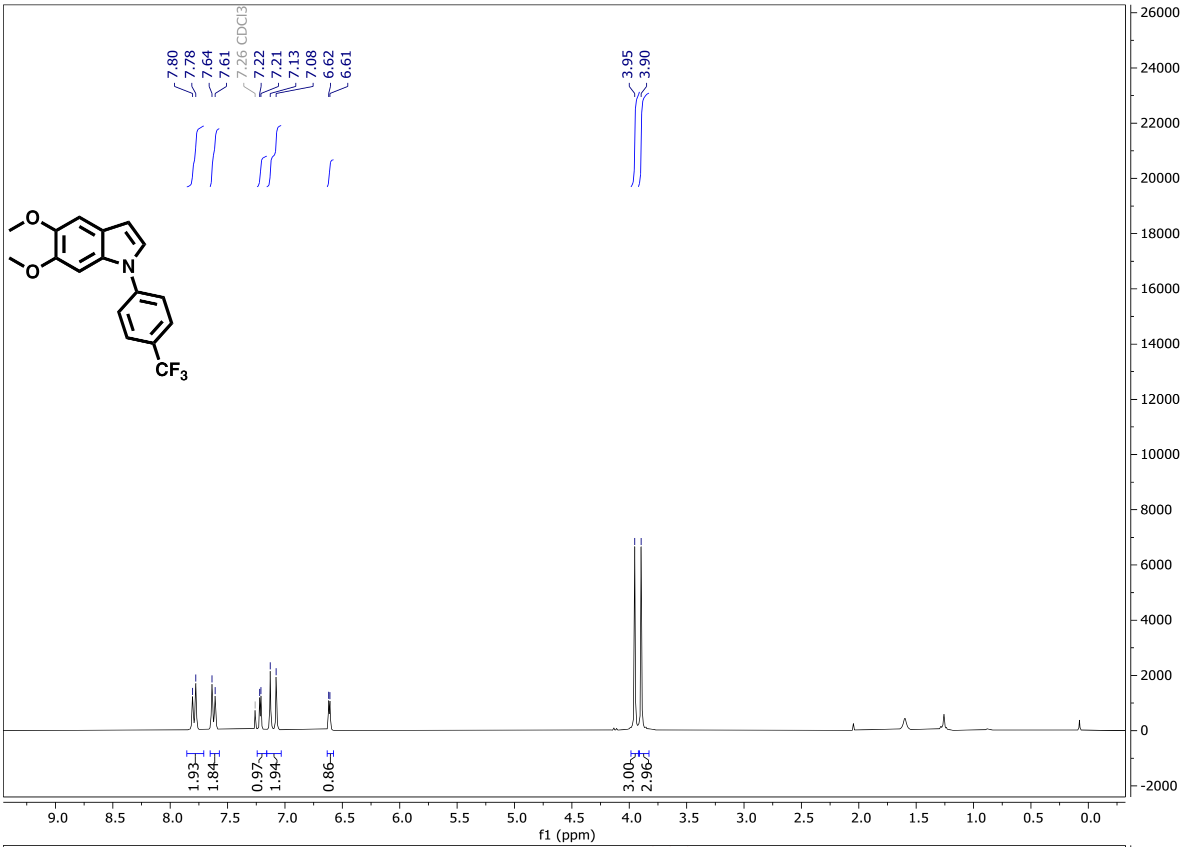

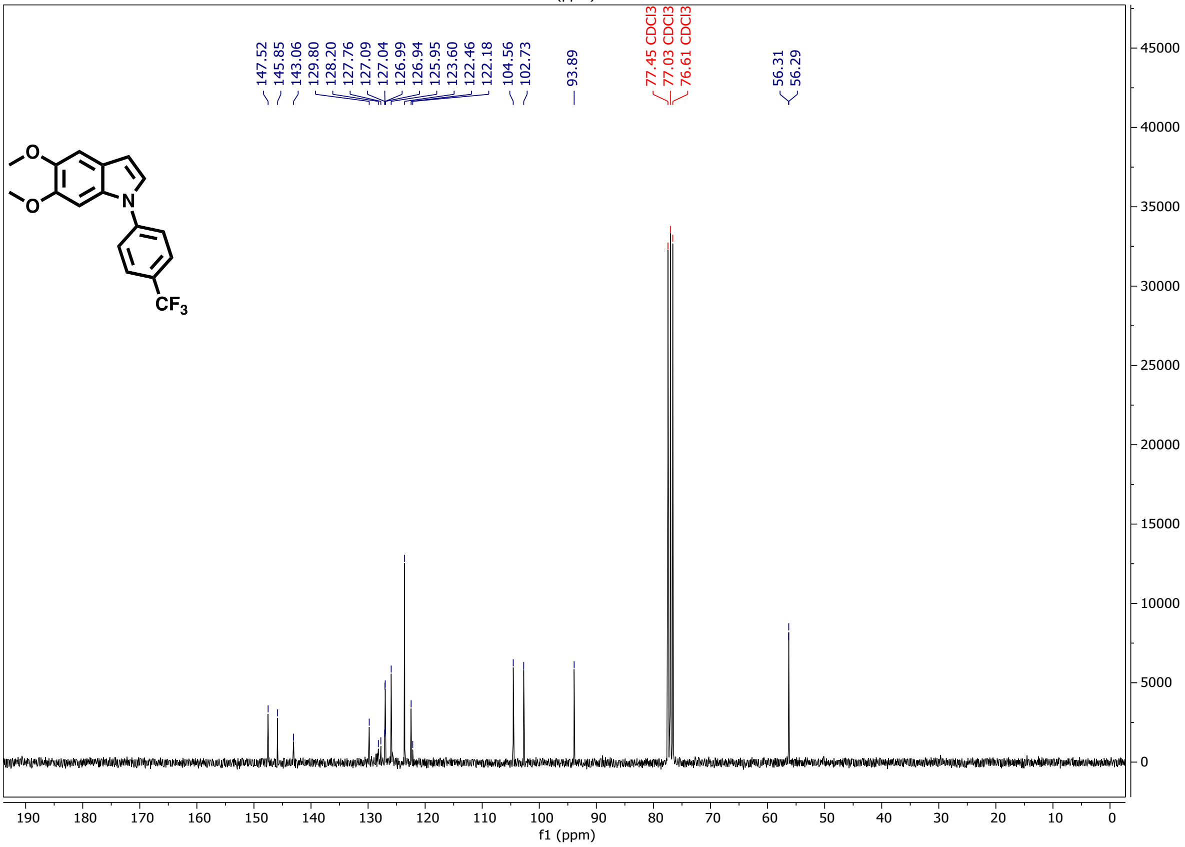

Supplementary Figure 23. ^{1}H and ^{13}C spectra of compound **C2-Gd5**

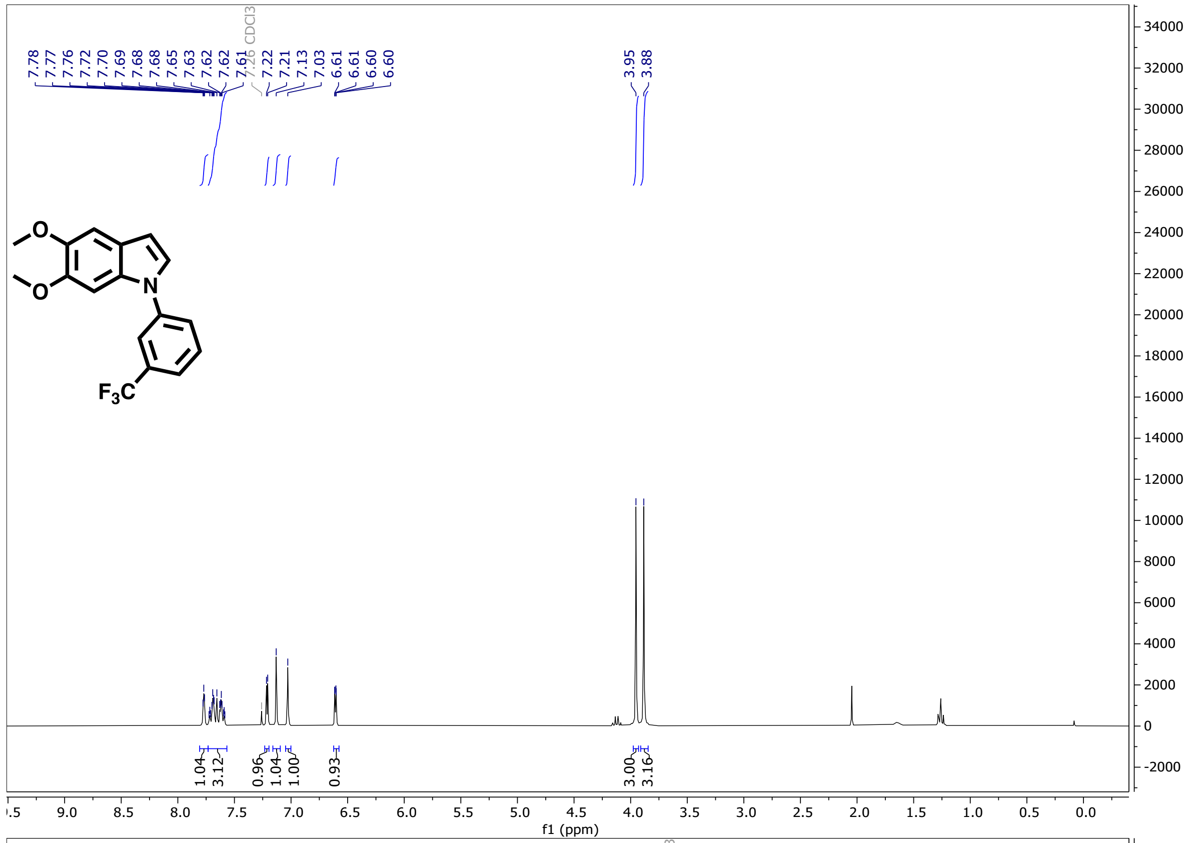

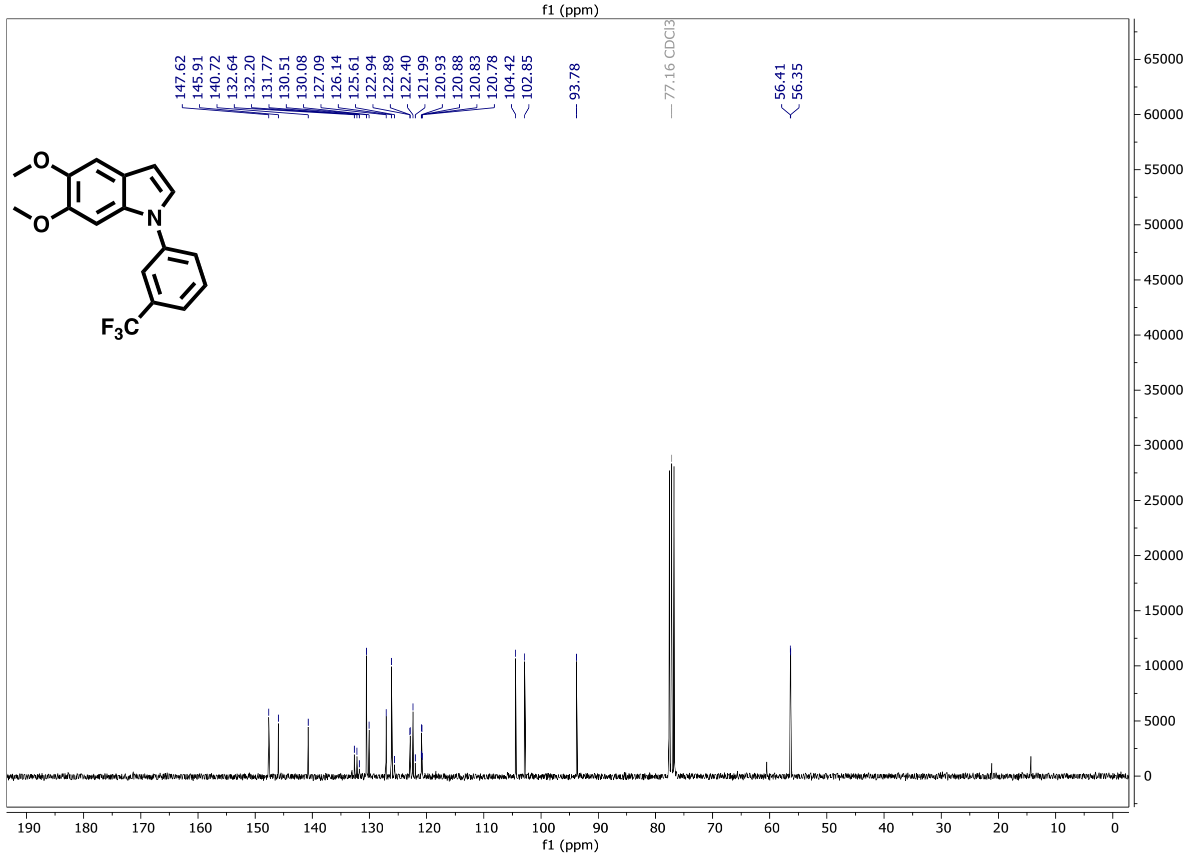

Supplementary Figure 24. ^{1}H and ^{13}C spectra of compound **C2-Gd6**

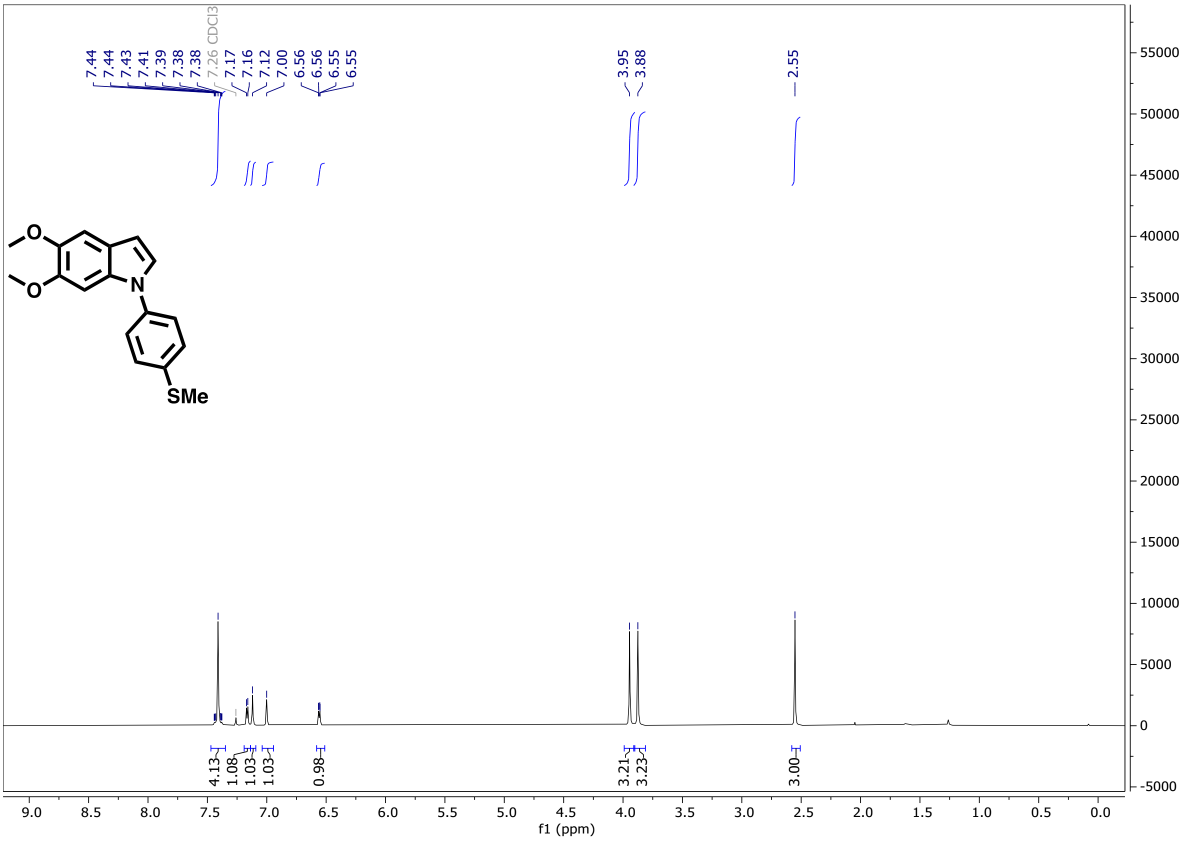

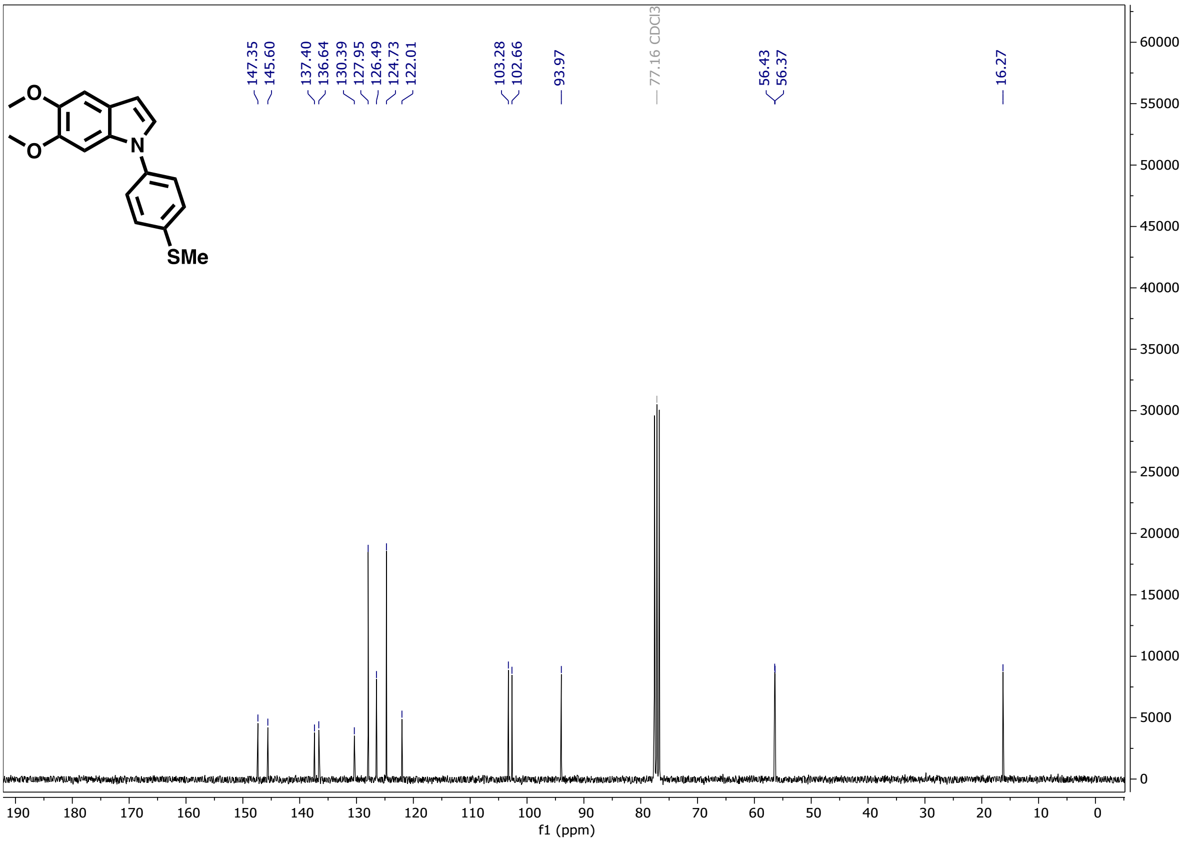

Supplementary Figure 25. ^{1}H and ^{13}C spectra of compound **C2-Gd7**

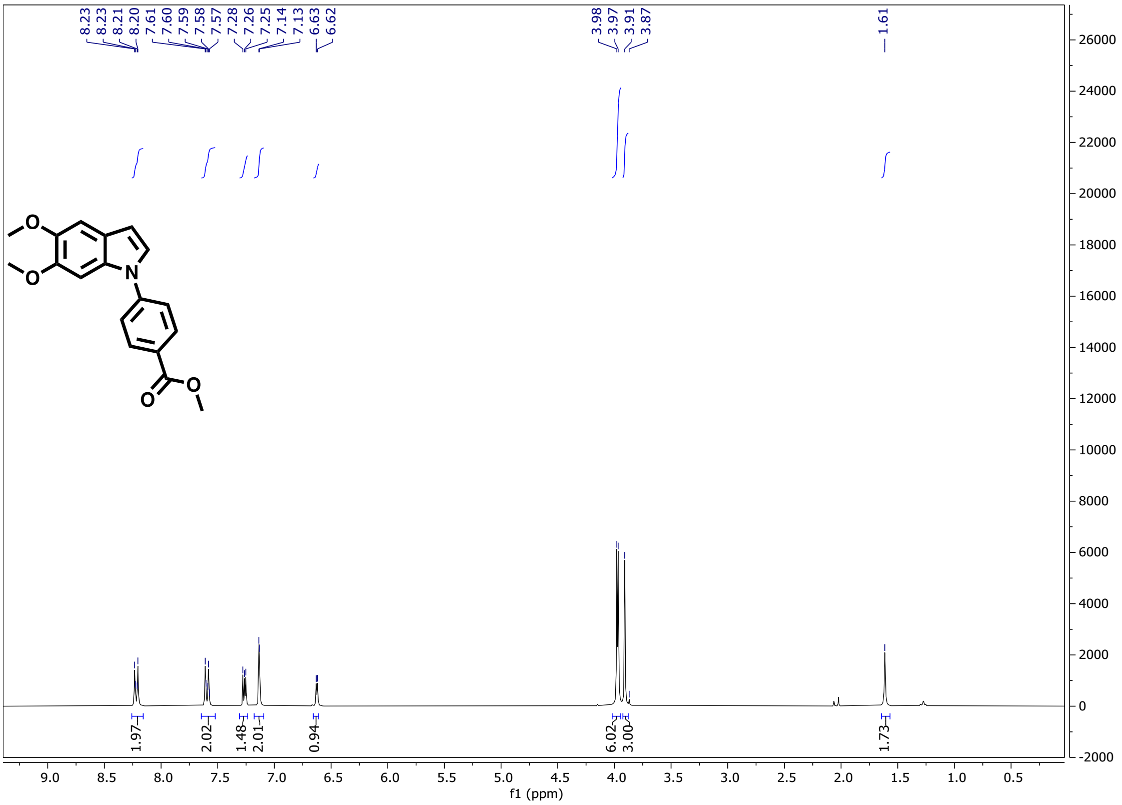

8.23 8.23 8.21 8.20 7.61 7.60 7.59 7.58 7.57 7.28 7.26 7.25 7.14 7.13 6.63 6.62
3.98 3.97 3.91 3.87
1.61
1.97 2.02 1.48 2.01 0.94 6.02 3.00 1.73
f1 (ppm)
O
O
N
O
O

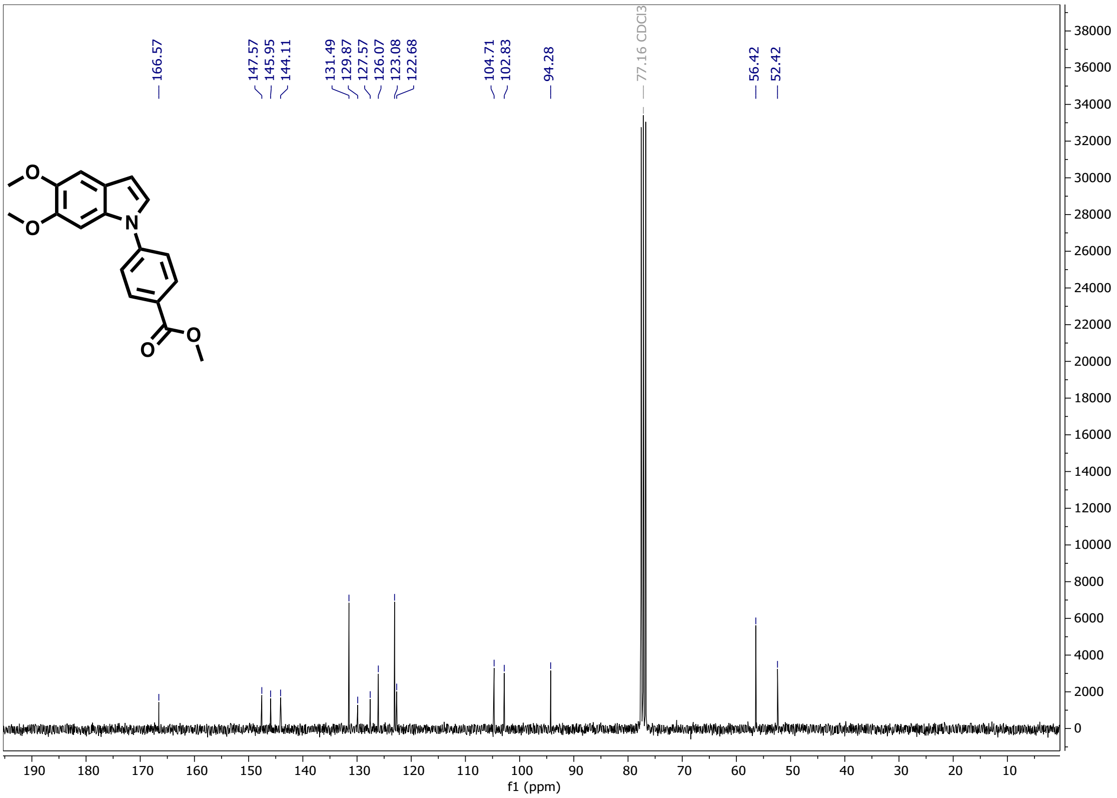

Supplementary Figure 26. ^{1}H and ^{13}C spectra of compound **C2-Gd8**

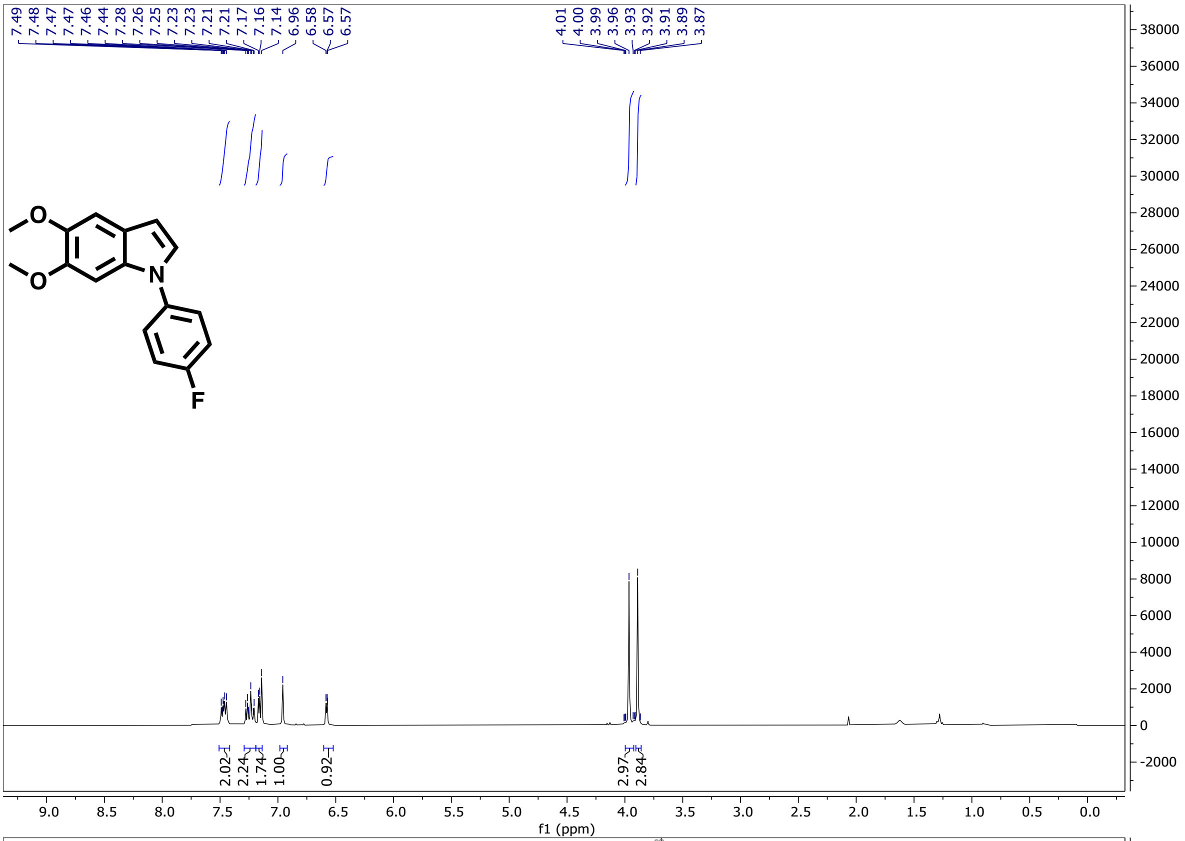

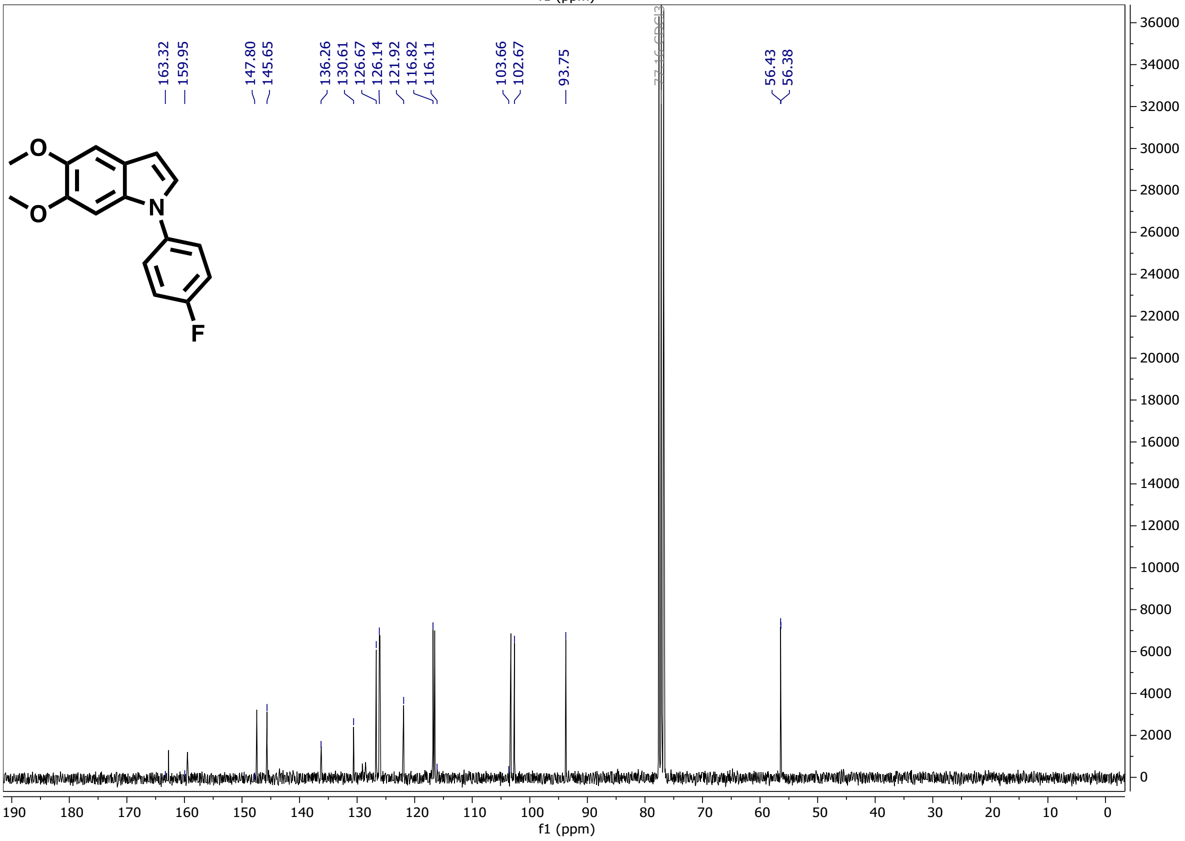

Supplementary Figure 27. ^{1}H and ^{13}C spectra of compound **C2-Gd9**

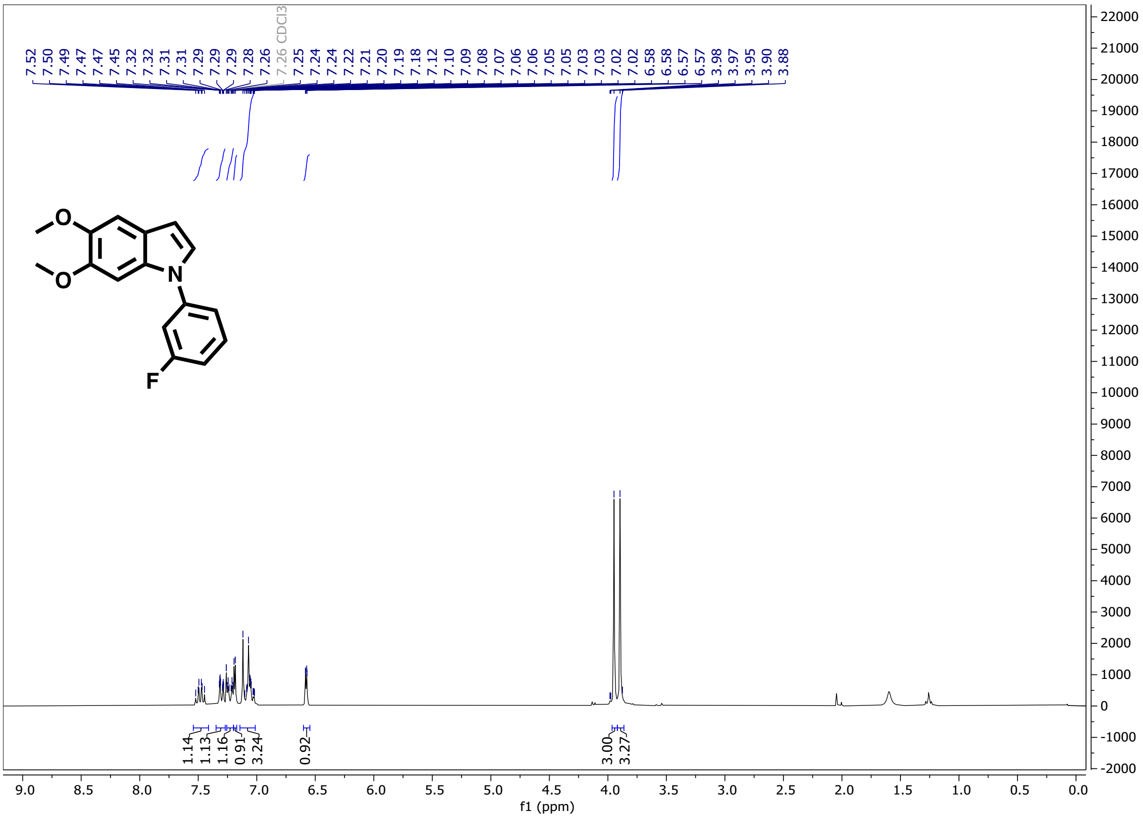

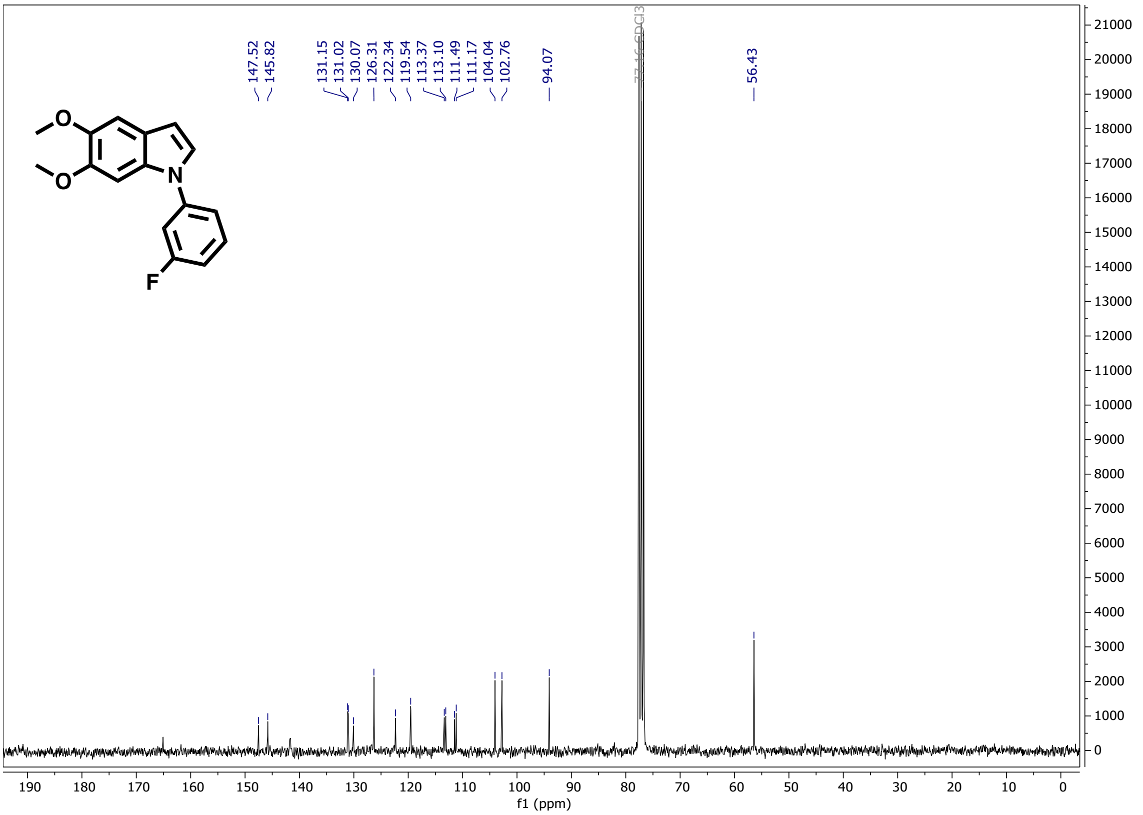

Supplementary Figure 28. ^{1}H and ^{13}C spectra of compound **C2-Gd10**

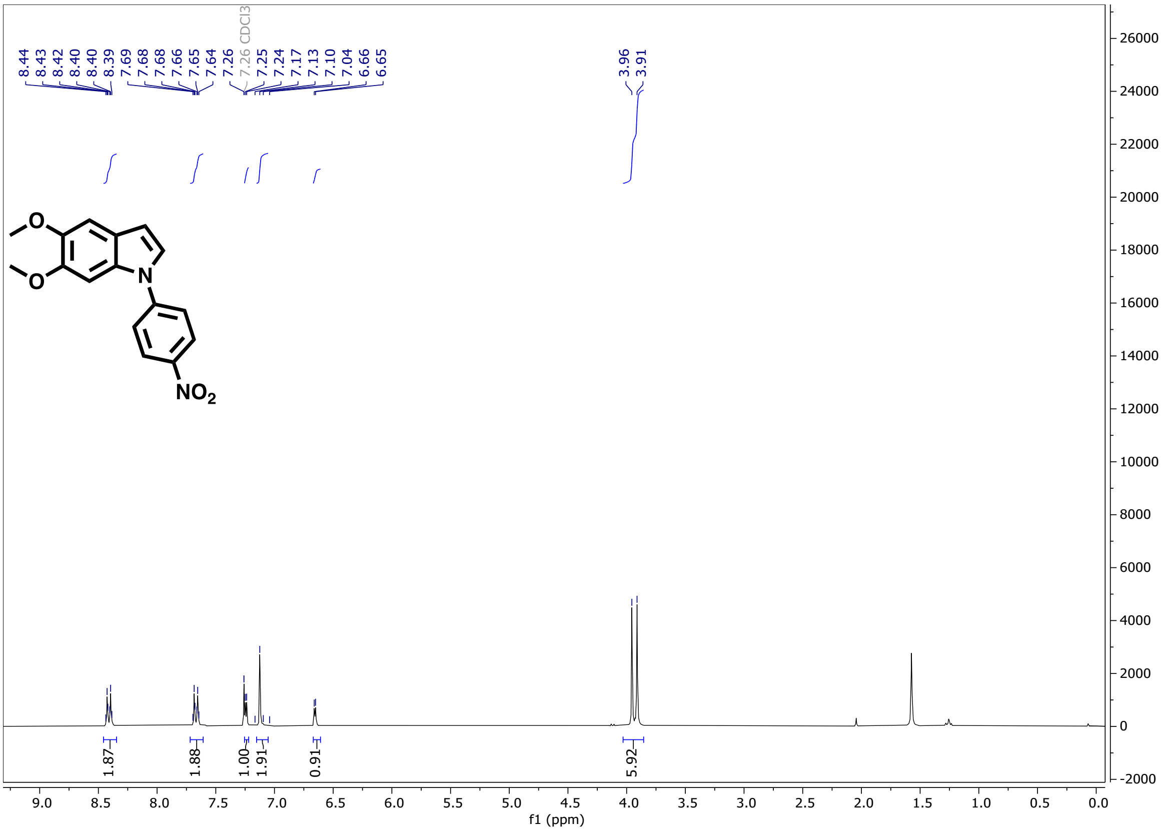

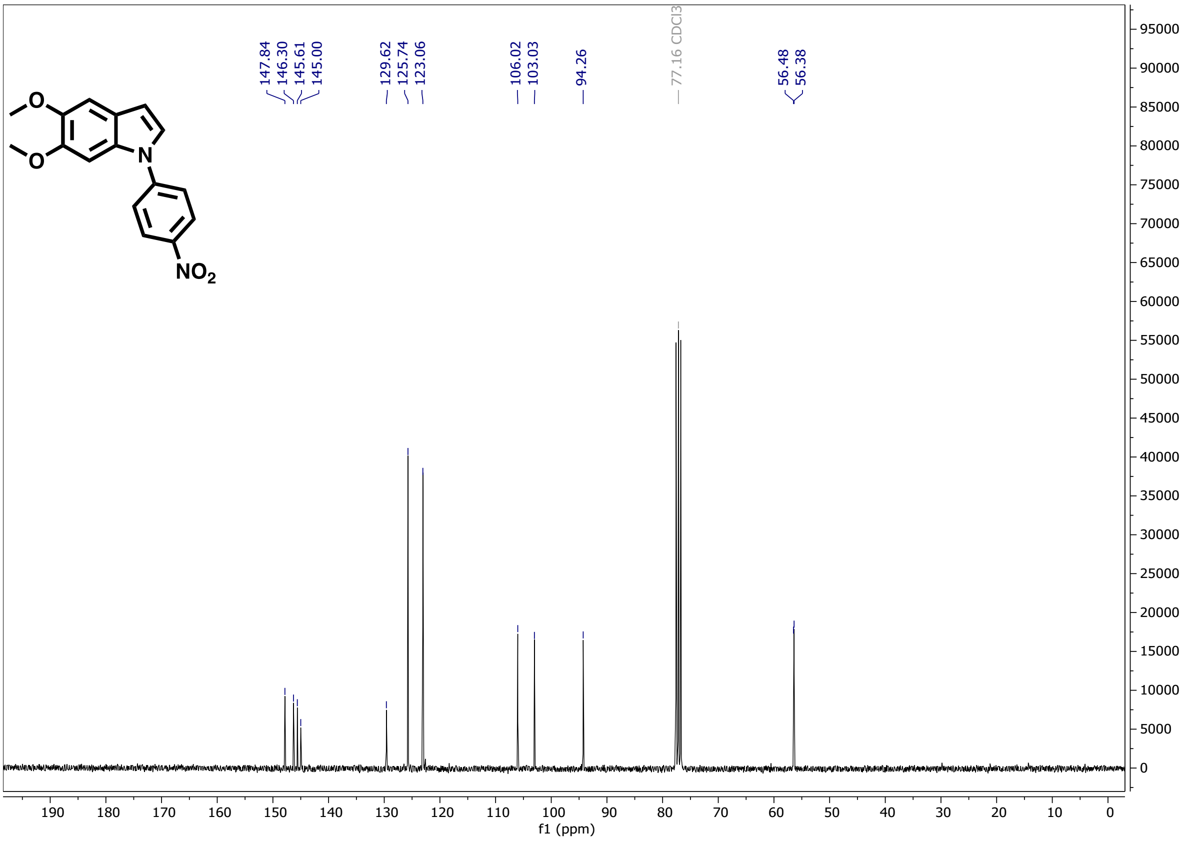

Supplementary Figure 29. ^{1}H and ^{13}C spectra of compound **C2-Gd11**

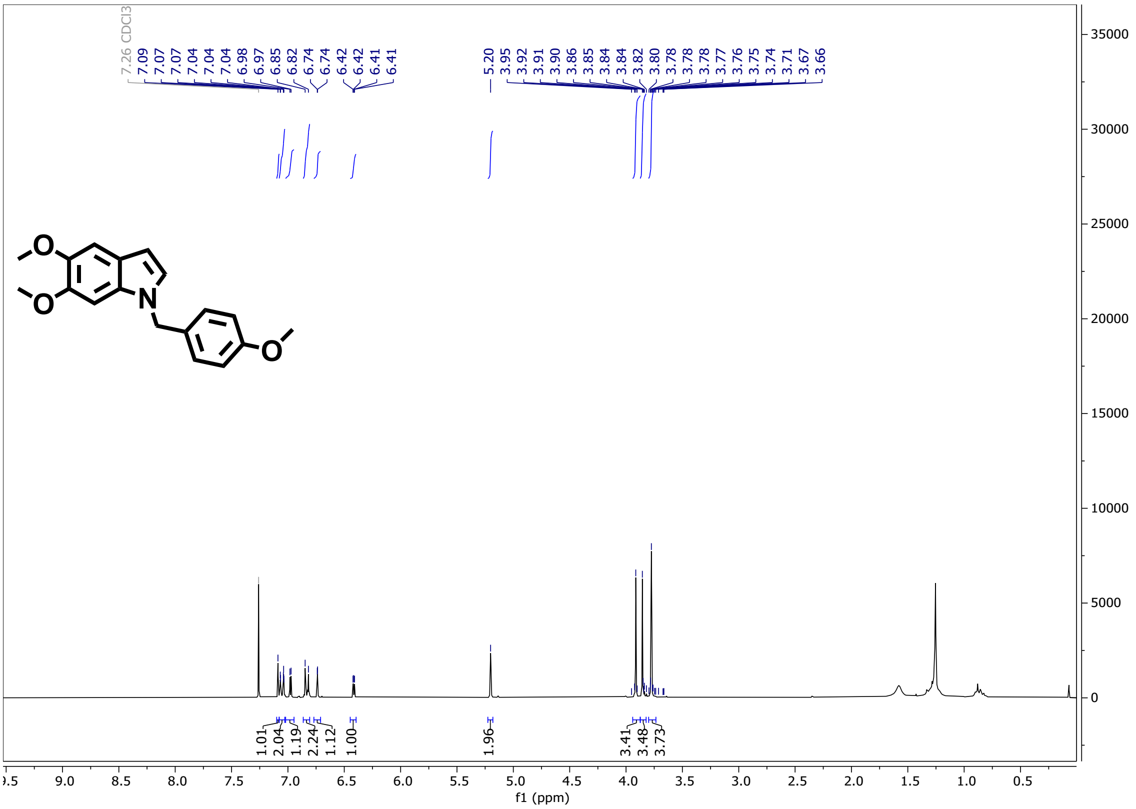

Supplementary Figure 30. ^{1}H and ^{13}C spectra of compound **C2-Gd13**

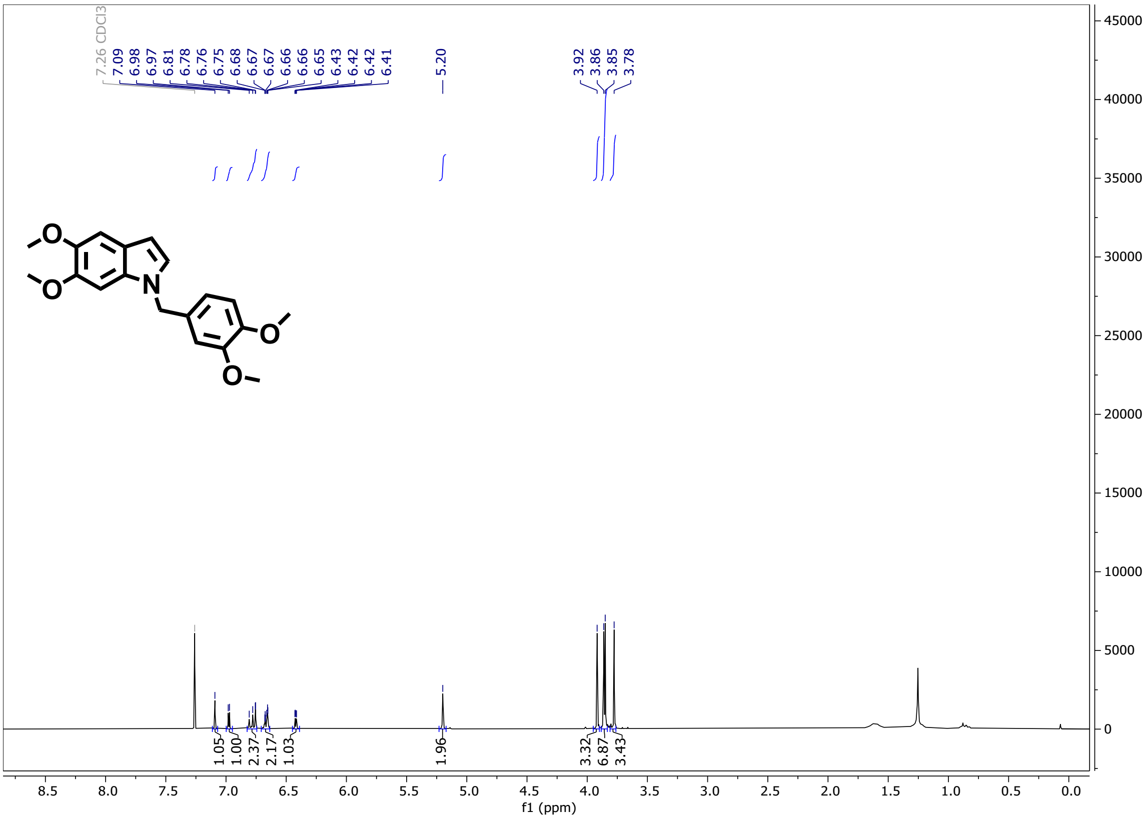

Supplementary Figure 31. ^{1}H and ^{13}C spectra of compound **C2-Gd14**

5. References

1. P. V. Balaji and S. Chandrasekaran, *Eur. J. Org. Chem*., **2016**, 2547-2554
2. A. M. Afanasenko, X. Wu, A. De Santi, W. A. M. Elgaher, A. M. Kany, R. Shafiei, M.-S. Schulze, T. F. Schulz, J. Haupenthal, A. K. H. Hirsch and K. Barta, *Angew. Chem., Int. Ed.,* **2024**, 63, e2023081
3. Haupenthal, J.; Baehr, C.; Zeuzem, S.; Piiper, *Int. J. Cancer* **2007**, 121, 206–210.
